# Multicenter OCT-Based Visual Field Representations via Segmentation-Free 3D CNNs: Forecasting, Longitudinal Variability, and Progression Detection

**DOI:** 10.1167/tvst.15.7.18

**Published:** 2026-07-13

**Authors:** Makoto Koyama, Hidenori Takahashi, Satoru Inoda, Chihiro Mayama, Yuta Ueno, Yoshikazu Ito, Tetsuro Oshika, Masaki Tanito

**Affiliations:** 1 Minamikoyasu Eye Clinic, Kimitsu, Japan; 2 Center for Cyber Medicine Research, Institute of Medicine, University of Tsukuba, Tsukuba, Japan; 3 Department of Ophthalmology, Jichi Medical University, Shimotsuke, Japan; 4 Department of Ophthalmology, Japan Community Health Care Organization Tokyo Shinjuku Medical Center, Tokyo, Japan; 5 Department of Ophthalmology, Faculty of Medicine, University of Tsukuba, Tsukuba, Japan; 6 Department of Ophthalmology, Shimane University Faculty of Medicine, Izumo, Japan

**Keywords:** visual field, optical coherence tomography, artificial intelligence, deep learning, perimetry

## Abstract

**Purpose:**

To evaluate segmentation-free three-dimensional convolutional neural networks (3DCNNs) that derive visual field (VF)-shaped representations from macular optical coherence tomography (OCT) volumes (OCT-based estimated VF; OCT-VF) and to assess their performance in forecasting future Humphrey field analyzer (HFA) measurements and characterizing longitudinal variability in clinical datasets.

**Methods:**

The 3DCNN models were trained on 129,007 heterogeneous real-world paired OCT–HFA datasets from 13,366 patients (24,313 eyes) across five institutions using 10-fold cross-validation. Forecasting accuracy for the last HFA visit was evaluated using HFA-only forecasts and hybrid models that combined HFA-anchored intercept with longitudinal slopes derived from both OCT-VF and HFA (AOS-AVG). Longitudinal variability was assessed as residual deviations from linear trends using jackknife resampling.

**Results:**

The AOS-AVG yielded the lowest forecasting errors across endpoints and significantly reduced mean absolute error compared with HFA-only forecasts (pointwise thresholds: 24-2, 2.45 vs. 2.68 dB; 10-2, 2.44 vs. 2.65 dB; both *P* < 0.001). OCT-VF showed significantly lower residual variability than HFA (MD: 24-2, 0.79 vs. 1.13 dB; 10-2, 0.87 vs. 1.07 dB; both *P* < 0.001). Eyes classified as progression-positive by OCT-VF but negative by HFA exhibited greater perimetric variability and steeper HFA MD decline than true-negative eyes.

**Conclusions:**

Hybrid forecasting approaches that incorporate OCT-VF–derived slope information can improve prediction of future visual-field outcomes. The lower variability of OCT-VF representations may also enable detection of progression-related signals that remain obscured by perimetric noise in routine longitudinal monitoring.

**Translational Relevance:**

OCT-VF may serve as a complementary tool for precision disease monitoring alongside current automated perimetry.

## Introduction

Visual field (VF) testing is crucial for diagnosing and monitoring various ocular conditions, particularly glaucoma, a leading cause of irreversible blindness worldwide.^1^^–^^3^ The Humphrey field analyzer (HFA; Carl Zeiss Meditec, Jena, Germany) remains the clinical standard for VF assessment, but HFA measurements require sustained attention and repeated responses over several minutes and are known to exhibit substantial test–retest variability, particularly in advanced disease.^3^^–^^5^ Repeated VF testing is also time-consuming and burdensome for patients and clinics. These characteristics have motivated the search for complementary biomarkers that can enhance longitudinal monitoring.

Optical coherence tomography (OCT) has transformed ophthalmic imaging by providing high-resolution and reproducible measurements of retinal structures that require only brief fixation for several seconds rather than prolonged response-based testing.^6^ OCT-based structural parameters have been reported to exhibit lower longitudinal variability than functional testing, particularly for disease monitoring.^7^ Conventional structural metrics, such as peripapillary retinal nerve fiber layer and macular ganglion cell complex (GCC) thickness, have demonstrated clinically meaningful associations with VF loss.^8^^–^^11^ However, their ability to fully characterize the complex and spatially heterogeneous nature of functional progression remains an area of active investigation. Recent advances in artificial intelligence (AI) have therefore explored using OCT data—ranging from single-layer thickness maps to complete volumetric scans—to generate VF-related outputs, including pointwise sensitivity estimates mapped to standard VF test grids (24-2 or 10-2), as well as global functional indexes, through machine learning and deep learning models.^12^^–^^27^ Conceptually, these approaches can be viewed as data-driven dimensionality reduction techniques that transform high-dimensional OCT structural information into lower-dimensional vector representations aligned with functional measurement domains. The target values used for training are derived from HFA, but once learned, the mapping itself constitutes an OCT-based representation that can be analyzed over time; it does not directly measure visual function.

A variety of OCT-based estimated visual field (OCT-VF) models have been proposed, ranging from two-dimensional (2D) approaches based on pre-segmented thickness maps or selected B-scans to three-dimensional (3D) models that use the full volume. Although 2D methods offer computational efficiency, they may be influenced by pre-processing choices. Segmentation-based approaches depend on segmentation quality and device-specific implementations,^12^^–^^15^ and B-scan–based approaches inherently provide only partial sampling of the imaged region.^16^^–^^22^ In contrast, 3D convolutional neural network (3DCNN) models^28^ can operate directly on complete OCT volumes, leveraging voxel-level information while preserving the full spatial context of the structural data without requiring explicit layer summarization.^23^^–^^25^ Related volumetric learning frameworks have also been explored for structural–functional association and glaucoma detection, as well as for predicting future OCT structure from prior volumetric scans.^27^^,^^29^ Our previous research showed that training on comprehensive datasets, including various ocular conditions, yielded better performance in estimating VF from 3D OCT images than glaucoma-specific training.^26^ Building on this foundation, the present study extends the approach to a large, multicenter cohort and focuses on the longitudinal behavior of OCT-derived VF representations.

Despite rapid progress in OCT-VF modeling, most prior studies have emphasized cross-sectional accuracy against HFA (for example, mean absolute error at a single time point) and have provided limited information about how OCT-VF behaves as a longitudinal signal.^12^^–^^26^ In particular, it remains unclear whether the reduced noise expected from an algorithmic, OCT-based representation translates into lower residual variability over time, earlier detection of significant progression trends, and how its trend-based progression signal compares with that of HFA and standard OCT metrics such as macular GCC thickness. Moreover, little is known about whether combining longitudinal OCT-VF and HFA information can improve the forecasting of future VF outcomes in routine clinical follow-up.

Therefore, in this study, we trained segmentation-free 3DCNN models that map macular OCT volumes to VF-shaped outputs in a heterogeneous, real-world multicenter dataset comprising 89,711 paired 24-2 tests and 39,296 paired 10-2 tests from more than 13,000 patients and evaluated these OCT-derived VF vectors as structure-derived functional biomarkers for longitudinal analysis. Specifically, our aims were to: (1) train and validate 3DCNN models for deriving VF-like representations from macular OCT images across a diverse clinical population; (2) assess whether hybrid approaches combining HFA-anchored intercept with slope information derived from OCT-VF, alone or in combination with HFA-derived slopes, can improve forecasting accuracy for future HFA measurements; (3) compare the longitudinal variability of OCT-VF with that of HFA measurements using residual analysis to provide a foundational basis for its predictive performance; (4) evaluate progression detection characteristics of OCT-VF relative to HFA as a reference standard; and (5) compare OCT-VF progression signals with those from conventional structural OCT metrics (macular GCC thickness) to determine whether volumetric deep learning analysis provides complementary information beyond single-layer thickness measurements. This research explores whether this alternative analysis of volumetric OCT data offers complementary information for disease monitoring that may enhance clinical decision-making when used alongside conventional perimetry. However, validation against clinical outcomes will be necessary to establish definitive clinical utility.

## Methods

### Study Design and Participants

The Institutional Review Boards of Shimane University Hospital (IRB No. KS20230719-3, approved on Aug 10, 2023) and Jichi Medical University (IRB No. Rindai24-130, approved on February 4, 2025) approved this retrospective multicenter study. Participating institutions included Shimane University Hospital, Shimane, Japan; Jichi Medical University, Tochigi, Japan; JCHO Tokyo Shinjuku Medical Center, Tokyo, Japan; Omiyahamada Eye Clinic, Saitama, Japan; and Minamikoyasu Eye Clinic, Chiba, Japan. This study adhered to the tenets of the Declaration of Helsinki. Because of the retrospective nature of the study, the requirement for informed consent was waived by the respective IRBs. An opt-out approach was used, and study information was publicly available on each institution's website and premises, allowing patients to decline participation. We retrospectively collected data from patients who underwent macular OCT imaging or HFA testing between August 5, 2004, and February 12, 2025.

### Inclusion Criteria

We retrospectively included all consecutive eyes with either (1) at least one macular OCT scan or (2) at least one VF test using the HFA with a 30-2, 24-2, or 10-2 test pattern using Swedish Interactive Threshold Algorithm (SITA) strategy. To reflect real-world clinical practice, no exclusions were made based on ocular diagnosis, and both SITA-Standard and SITA-Fast strategies were used for model training.

### Overview of Datasets and Study Design

We trained 3DCNN models using 129,007 paired OCT–HFA datasets from 13,366 patients (24,313 eyes) across five institutions within a patient-wise 10-fold cross-validation framework. Independent from the training process, a longitudinal test dataset and a single-center macular GCC subset were constructed for progression and comparative analyses, respectively.

### Training Data Acquisition and Preprocessing

OCT images were acquired using RS-3000, RS-3000 Advance, RS-3000 Advance2, or Mirante devices (Nidek, Gamagori, Japan) with a 9 × 9 mm volumetric scan protocol centered on the macular, performed under both dilated and undilated conditions. This scan area covers the entire macular region. It extends nasally to include part of the optic nerve head and adjacent peripapillary retinal nerve fiber layer, with the nasal edge typically reaching approximately the center of the optic disc. We applied quality control criteria by excluding OCT scans with signal strength index (SSI) <7 and excluding peripheral test points from 30-2 VF tests (trimmed to match the 24-2 pattern). Reliability thresholds for training HFA data were set at <33% for all three indexes: false-positive rate, false-negative rate, and fixation loss rate. This relatively lenient threshold was selected to maximize the training data volume and enhance model robustness against real-world artifacts, consistent with previous studies.^12^^,^^16^^,^^26^ We paired OCT scans with HFA tests performed within 90 days of the OCT acquisition date, selecting the HFA test with the smallest absolute time difference when multiple tests were available. For eyes with both 24-2 and 10-2 test patterns, we included all available data. For eyes with only one test pattern (e.g., only 24-2 but not 10-2) within the 90-day window, we used mask values to indicate missing data points. We included all eligible paired data from eyes with multiple OCT scans and VF tests in the model development dataset, using the raw pointwise thresholds and mean deviation (MD) values without any clipping.

### Model Architecture and Configuration

**Figure 1.**
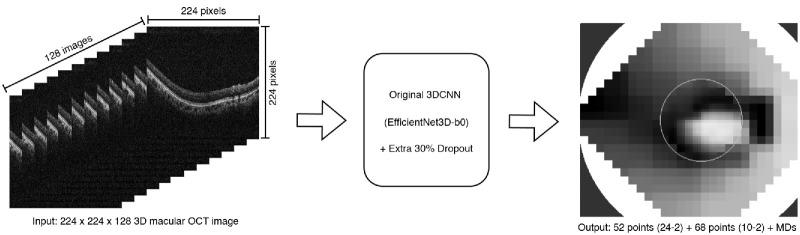
Schematic representation of the segmentation-free 3DCNN model. We based the model on the EfficientNet3D-b0 architecture with an additional 30% dropout layer, which directly connects to the output layer and 122 outputs: VF pointwise thresholds (52 points for 24-2 and 68 points for 10-2) and their respective MD values.

We adopted a segmentation-free 3DCNN model based on the EfficientNet3D-b0 architecture^30^ with a 30% dropout layer to reduce overfitting (Fig. 1). This architecture was selected for its optimal balance of model complexity and computational efficiency for 3D volumetric data. The model was trained from scratch using a comprehensive OCT dataset. Input OCT images were standardized to a resolution of 224 × 224 × 128, and z-score normalization was applied to both images and VF data. The model outputs estimated VF pointwise thresholds (52 points for 24-2 and 68 points for 10-2 patterns) and their respective MDs. Following previous reports,^13^^,^^26^ we used horizontal flipping for left eye data and vertical flipping as data augmentation techniques. Model training used the Adam optimizer with a mini-batch size of 4 and a variable learning rate schedule, minimizing mean squared error while accounting for missing data points through masked backpropagation.

### Model Training With 10-Fold Cross-Validation

We used a 10-fold cross-validation approach using stratified sampling to ensure balanced distribution across folds. Patients were stratified by institution and further divided into 16 subgroups based on test pattern (24-2 or 10-2), MD value (above or below the mean), and number of measurements per eye (above or below the mean). This stratified, patient-wise splitting ensured that data from the same patient did not appear in more than one set, thereby preventing data leakage while maintaining representative distributions of disease severity and measurement frequency across the training, validation, and test sets (8:1:1 ratio).

In 10-fold cross-validation, the entire dataset was divided into 10 equal parts (“folds”). The model training was repeated 10 times, and in each round, a different fold was set aside as the test set, another fold as the validation set, and the remaining eight folds as the training set. By rotating the role of each fold through all combinations, every sample in the dataset was used exactly once for testing, once for validation, and eight times for training. This process resulted in 10 trained models, each optimized on a different combination of training data while being validated and tested on mutually exclusive patient populations. This approach enables a more robust evaluation of model performance than simply using one stratification of test and training data. Importantly, this cross-validation process was used primarily to generate the trained models and evaluate cross-sectional estimation performance. The longitudinal variability analysis (described below) was conducted as a separate post-hoc analysis using these trained models.

### Longitudinal Test Dataset Creation

Following model training, we constructed a separate longitudinal dataset to evaluate temporal variability, forecasting performance, and progression-related characteristics of OCT-VF and HFA measurements. The model training dataset was intentionally designed to maximize generalizability and therefore included examinations performed with both SITA-Standard and SITA-Fast strategies, eyes with limited numbers of visits, and examinations with relatively permissive reliability criteria. In contrast, the longitudinal analyses required more consistent testing conditions and sufficient repeated observations to allow reliable estimation of temporal trends. In addition, stricter reliability criteria were applied for evaluation, including a lower threshold for false-positive rates. Therefore a dedicated longitudinal dataset was constructed using stricter criteria for test reliability and follow-up structure, as described below.

For the OCT-VF dataset, we generated estimates from all available macular OCT images (SSI ≥7) using our trained 3DCNN models. At the patient level, the longitudinal dataset represents a subset of the training dataset: all patients in the longitudinal dataset were also included in the training dataset. Crucially, to prevent data leakage, we assigned each patient deterministically to the specific 3DCNN model where that patient was part of the test fold during the 10-fold cross-validation process. In other words, every OCT-VF estimate was generated exclusively by a model that had never seen that patient's data during training.

For the HFA dataset, examinations not performed using SITA-Standard were excluded to maintain consistency of testing conditions and reduce potential variability, since SITA-Standard is generally considered to have lower test–retest variability than SITA-Fast.^5^ We excluded only the first HFA test for each eye to reduce learning effects. To improve the reliability of the HFA reference used in the longitudinal analysis, we applied specific test selection criteria based on an existing guideline and prior evidence. We excluded tests with false-positive rates ≥15% in accordance with the manufacturer's recommendations for perimetric reliability.^31^ However, tests were retained regardless of false-negative or fixation loss rates. Elevated false-negative rates are known to correlate with defect severity in glaucoma rather than reflecting poor patient attention alone.^32^ Likewise, fixation loss has been reported to be a less accurate indicator of perimetric reliability compared with other indexes.^33^ This strategy—applying a strict false-positive threshold while retaining tests with higher false-negative or fixation loss rates—was intended to reduce spurious variability in the HFA reference without systematically excluding eyes with advanced disease. Notably, this false-positive threshold is stricter than that used during model training (<33%), reflecting our intent to use a relatively more reliable subset of HFA measurements for longitudinal reference purposes. We used the pointwise thresholds and MD values without any clipping for both datasets, except that OCT-VF threshold values below 0 dB were set to 0 dB.

To minimize within-subject variability, when multiple measurements were taken on the same day, we used the last HFA measurement (as repeated testing typically occurs when the first test is unreliable) and calculated SSI-squared weighted averages for OCT-VF measurements. Specifically, for each test point, the weighted average was computed as:

$$\begin{array}{ccc} & & \mathrm{Weighted}\;\mathrm{average}\\ & & \;=\sum \left(\mathrm{OCT}-\mathrm{VF}\times \mathrm{SS}I^{2}\right)/\sum \left(\mathrm{SS}I^{2}\right) \end{array}$$

where the summation is over all OCT scans acquired on the same day. SSI-squared weighting (which gives more weight to higher-quality scans while still incorporating information from all available scans) was selected based on preliminary testing comparing linear SSI weighting and unweighted averaging. To align both datasets temporally, when the first and/or last measurement dates differed between OCT-VF and HFA, we iteratively trimmed excess measurements in chronological order from the ends of the longer series until the sequences no longer inverted in time. To ensure identical measurement counts per eye between modalities, measurements were paired using a greedy matching procedure. For each observation in the modality with fewer examinations, the temporally closest measurement from the other modality within ±90 days was selected without replacement based on the smallest absolute inter-test date difference. Observations without a corresponding measurement within 90 days were discarded. Eyes with fewer than six paired measurements were excluded. To characterize sampling density, the distribution of HFA examination counts per eye was examined.

### Forecasting the Last HFA: Task Definition and Models

We evaluated out-of-sample forecasting of the last HFA test for each eye using longitudinal data available strictly prior to the last-visit timepoint. For both pointwise thresholds (24-2 and 10-2 test patterns) and mean deviation (MD), we fitted simple linear trends to the available history and extrapolated to the last HFA measurement. All models were fit separately for each eye and (for pointwise analyses) each test location.

The HFA regression forecast (HFA-RF) used only HFA measurements obtained prior to the last HFA. For each eye and location, we regressed the observed HFA value on time using all preceding HFA visits and predicted the last-visit value by linear extrapolation to the last HFA timestamp. For MD, the same procedure was applied to the eye-level MD series.

The OCT-VF regression forecast (OCT-RF) used only OCT-VF values strictly earlier than the last HFA. For each eye and location, we regressed the OCT-VF value on time using all OCT-VF tests with timestamps preceding the last HFA and extrapolated to the last HFA timestamp. For MD, we fit the same linear model to the eye-level OCT-VF MD history. No HFA measurements entered the OCT-RF fit.

The HFA-anchored OCT-VF slope forecast (AOS) combines an HFA-anchored intercept with an OCT-VF–derived slope estimated prior to the last HFA. The intercept anchor was defined as the simple average of all HFA measurements preceding the last HFA, thereby preserving the eye- and location-specific functional level while discarding HFA slope information. The slope was estimated from OCT-VF tests preceding the last HFA, as in OCT-RF. The final forecast was obtained by linear extrapolation to the last HFA timestamp using this anchored intercept and OCT-VF slope. For MD, the same procedure was applied at the eye level.

The averaged-slope HFA-anchored forecast (AOS-AVG) extends the AOS framework by incorporating slope information from both modalities. The intercept anchor was defined identically to AOS as the simple average of all HFA measurements preceding the last HFA. The slope was computed as the arithmetic mean of the OCT-VF–derived slope and the HFA-derived slope, each estimated using measurements strictly prior to the last HFA. The final forecast was obtained by linear extrapolation to the last HFA timestamp using this anchored intercept and averaged slope. For MD, the same procedure was applied at the eye level.

For each eye and test location, let *t_last_* denote the timestamp of the last HFA measurement. The four forecasting models are defined as:

$$\begin{array}{c} \mathrm{HFA}-\mathrm{RF}:\;\hat{y}={\bar{y}}_{HFA}+\beta_{HFA}\;\left(t_{last}-{\bar{t}}_{HFA}\right) \end{array}$$

$$\begin{array}{c} \mathrm{OCT}-\mathrm{RF}:\;\hat{y}={\bar{y}}_{OCT}+\beta_{OCT}\;\left(t_{last}-{\bar{t}}_{OCT}\right) \end{array}$$

$$\begin{array}{c} \mathrm{AOS}:\;\hat{y}={\bar{y}}_{HFA}+\beta_{OCT}\;\left(t_{last}-{\bar{t}}_{HFA}\right) \end{array}$$

$$\begin{array}{c} \mathrm{AOS}-\mathrm{AVG}:\;\hat{y}={\bar{y}}_{HFA}+\frac{\beta_{OCT}+\beta_{HFA}}{2}\;\left(t_{last}-{\bar{t}}_{HFA}\right) \end{array}$$

where ${\bar{y}}_{HFA}$ and ${\bar{y}}_{OCT}$ denote the mean of all prior HFA and OCT-VF values, respectively; β_*HFA*_ and β_*OCT*_ are slopes estimated from prior HFA and OCT-VF measurements; and ${\bar{t}}_{HFA}$ and ${\bar{t}}_{OCT}$ denote the mean timestamps of prior HFA and OCT-VF measurements, respectively.

Forecast accuracy was summarized as mean absolute error (MAE) between forecasts and the observed last-visit HFA values, computed per eye and location for pointwise endpoints and per eye for MD. Confidence intervals and paired model comparisons versus HFA-RF were obtained by patient-level bootstrap (B = 10,000) with paired resampling of all modalities within patient. For multiplicity, we applied the Holm step-down procedure within each endpoint (24-2 pointwise, 10-2 pointwise, 24-2 MD, 10-2 MD) across the three pre-specified comparisons (OCT-RF, AOS, and AOS-AVG vs HFA-RF) and reported Holm-adjusted *P* values. To harmonize inference across the four endpoints, statistical significance was declared at adjusted *P* < 0.0125, reflecting an additional Bonferroni correction over the four endpoint families. To evaluate performance across disease severity, forecast errors (absolute, signed, and squared) were stratified by observed last-HFA values and visualized using boxplots, with overlaid cubic smoothing curves to illustrate severity-dependent trends.

### Supporting Analyses and Implementation

To contextualize the forecasting results, we conducted several supporting analyses. Longitudinal variability was quantified as residual deviations from eye-specific linear trends using jackknife resampling. Mean deviation (MD) progression detection (slope ≤−0.5 dB/year, *P* < 0.025) was evaluated and compared across modalities. In addition, a single-center subset with concurrent macular GCC measurements was analyzed to compare volumetric 3DCNN-derived representations with conventional structural metrics. Finally, for model evaluation, best-available estimate (BAE) visual fields were generated from longitudinal HFA regression models to provide stable, noise-filtered perimetric references. Detailed statistical protocols and error-decomposition procedures are provided in the Supplementary Methods.

**Figure 2.**
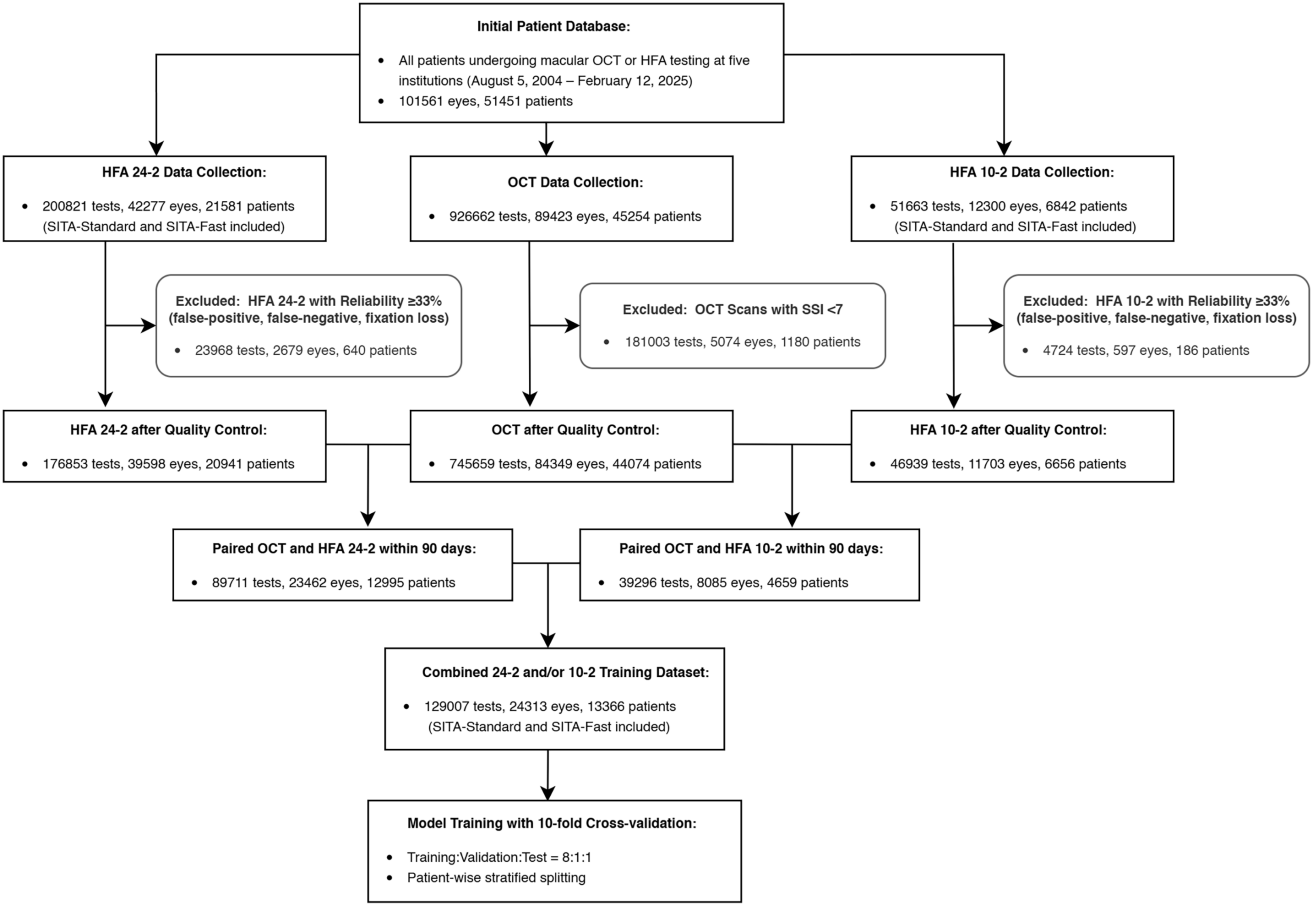
Study flowchart illustrating patient selection criteria and dataset construction for model training. Starting with 51,451 patients, exclusion criteria were applied, resulting in 13,366 patients included in the training dataset.

**Figure 3.**
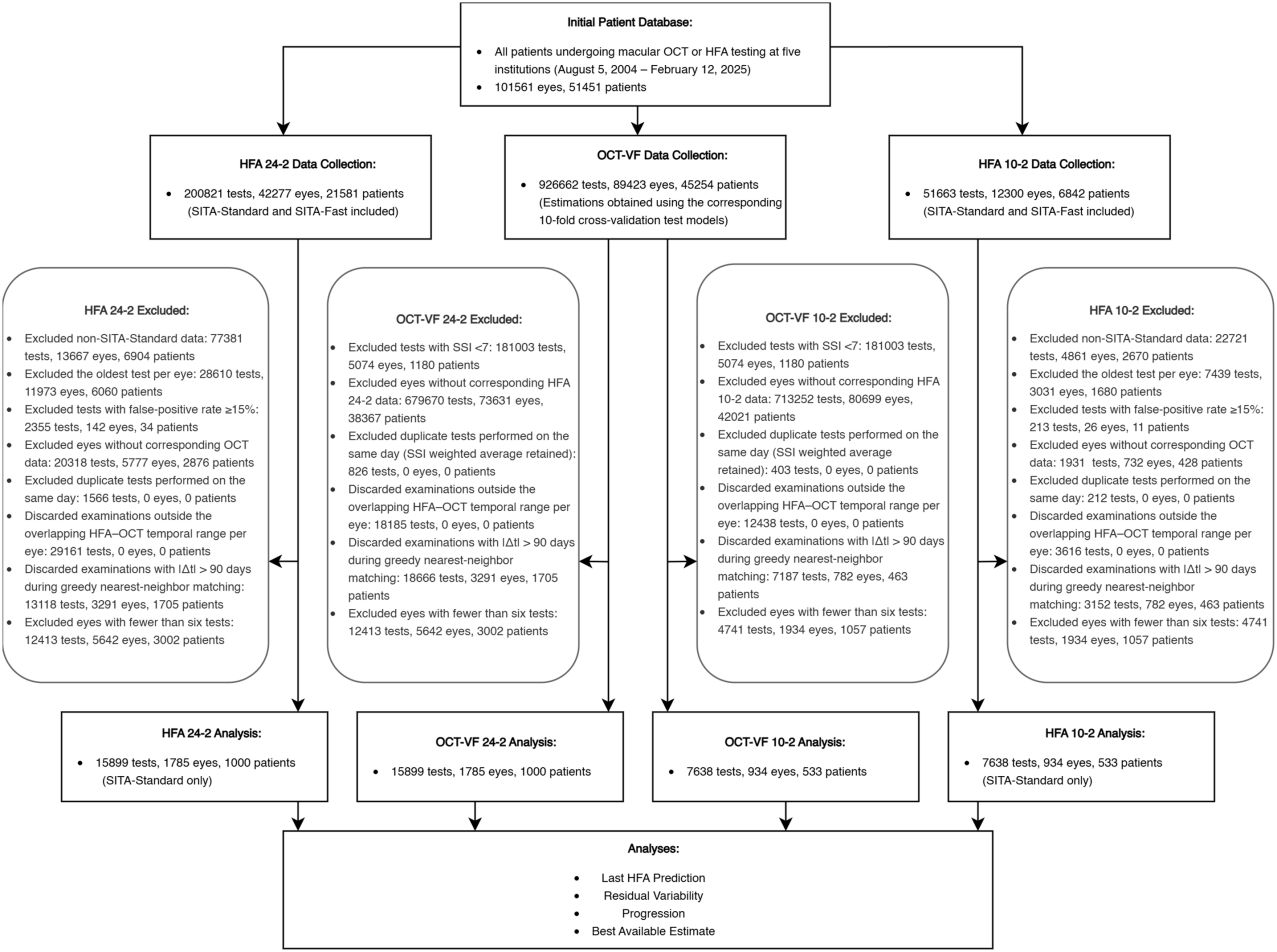
Flowchart illustrating patient and data selection for the longitudinal test dataset. Exclusion criteria and the number of excluded records at each step are shown. The resulting test dataset was used for model evaluation.

All statistical tests were two-sided. We performed all analyses using Python 3.12.9 with scikit-learn (1.6.1), statsmodels (0.14.4), and PyTorch (2.6.0). The study workflow and patient selection process are illustrated in Figure 2 (training dataset), Figure 3 (longitudinal test dataset), and Supplementary Figure S1 (GCC analysis dataset).

## Results

### Training and Test Dataset Characteristics

Table 1 summarizes the characteristics of the dataset used to train the 3DCNN models. For the longitudinal analysis comparing OCT-VF and HFA variability, we created corresponding time-series datasets for each measurement type from the same patient population. Table 2 summarizes the characteristics of these longitudinal test datasets used for comparative analysis. The HFA dataset had mean observation periods of 2068 ± 1037 days (24-2) and 1599 ± 574 days (10-2), whereas the OCT-VF dataset had periods of 2068 ± 1046 days (24-2) and 1596 ± 576 days (10-2). There was no significant difference in observation period for 24-2 (*P* = 0.369, Wilcoxon signed-rank test), whereas a small but statistically significant difference was observed for 10-2 (*P* < 0.001). To minimize any potential influence of small residual differences in observation period on the comparison of residual variability, the observation period was included as one of the covariates in the generalized estimating equations (GEE)^34^ analysis.

**Table 1.** Training Dataset Characteristics

Patient Characteristics	Value
Total number of patients	13,366
Number of patients in HFA 24-2	12,995
Number of patients in HFA 10-2	4659
Total number of eyes	24,313
Number of eyes in HFA 24-2	23,462
Number of eyes in HFA 10-2	8085
Total number of sets of paired data	129,007
Number of sets of paired data in HFA 24-2	89,711
Number of sets of paired data in HFA 10-2	39,296
Number of HFA 24-2 tests per eye (mean ± SD)	9.69 ± 7.61
Number of HFA 10-2 tests per eye (mean ± SD)	8.09 ± 5.14
Proportion of SITA-Standard strategy in HFA 24-2 (%)	46.5
Proportion of SITA-Standard strategy in HFA 10-2 (%)	47.2
Number of OCT tests per eye (mean ± SD)	5.31 ± 7.27
Mean deviation of the HFA 24-2 (dB, mean ± SD)	−5.93 ± 7.65
Mean deviation of the HFA 10-2 (dB, mean ± SD)	−8.03 ± 8.69
Age (years, mean ± SD)	65.0 ± 14.2
SSI (mean ± SD)	8.38 ± 1.03
Focus (D, mean ± SD)	−2.60 ± 3.56

**Table 2.** OCT-VF and HFA Longitudinal Test Dataset Characteristics

Characteristics	OCT-VF 24-2	HFA 24-2	OCT-VF 10-2	HFA 10-2
Number of patients	1000	1000	533	533
Number of eyes	1785	1785	934	934
Age (years)	67.3 ± 14.0	67.3 ± 14.0	66.7 ± 14.2	66.8 ± 14.2
First MD (dB)	−9.3 ± 8.3^*^	−8.9 ± 8.7	−10.7 ± 9.0^*^	−10.4 ± 9.2
Mean MD (dB)	−10.3 ± 8.5^*^	−9.7 ± 8.9	−11.5 ± 9.3^*^	−11.3 ± 9.5
Last MD (dB)	−11.1 ± 8.8^*^	−10.6 ± 9.3	−12.3 ± 9.7^*^	−12.1 ± 10.0
Number of tests	8.91 ± 2.71	8.91 ± 2.71	8.18 ± 1.92	8.18 ± 1.92
Inter-modality interval (days)	8.9 ± 23.3	9.0 ± 23.4	2.7 ± 13.6	2.8 ± 13.7
Follow-up duration (days)	2068 ± 1046	2068 ± 1037	1596 ± 576	1599 ± 574

Differences in mean observation periods between OCT-VF and HFA datasets reflect that the temporal alignment criteria were applied at the study level. All inter-modality intervals were constrained to ≤90 days. Inter-modality intervals were defined as the absolute time difference to the nearest examination of the opposite modality. Because nearest-neighbor relationships are directional, OCT→HFA and HFA→OCT values are not necessarily identical. The values are presented as the means ± standard deviations.

* MD values for OCT-VF are estimates derived from the OCT-VF models.

### Forecasting the Last Measurement

As shown in Supplementary Figure S2, although the number of examinations per eye was matched between modalities, the distribution decreased at higher visit counts. To evaluate forecasting performance under a general longitudinal scenario, we compared within-modality and cross-modality prediction errors at the last available measurement for each eye using only data strictly prior to that timepoint (Table 3). Across both test patterns (24-2 and 10-2) and both endpoints (pointwise and MD), prediction errors followed a consistent ordering, with the lowest errors for OCT-VF → OCT-VF forecasts, followed by HFA → HFA, HFA → OCT-VF, and the highest errors for OCT-VF → HFA. For example, in the 24-2 pointwise analysis, MAE was 1.24 ± 1.04 dB for OCT-VF → OCT-VF, 2.68 ± 1.74 dB for HFA → HFA, 3.80 ± 2.13 dB for HFA → OCT-VF, and 4.02 ± 2.38 dB for OCT-VF → HFA. Similar patterns were observed for 10-2 and for MD endpoints. Thus, within-modality forecasts yielded lower errors than cross-modality forecasts. In particular, forecasting HFA measurements from OCT-VF resulted in substantially larger errors than forecasting HFA from prior HFA measurements. Conversely, predictions targeting OCT-VF showed lower MAE than those targeting HFA across endpoints.

**Table 3.** Comparison of Within-Modality and Cross-modality Forecasting Errors

Test Pattern	Modality^*^	Pointwise MAE (dB)	MD MAE (dB)
24-2			
	HFA → HFA	2.68 ± 1.74	1.42 ± 1.86
	OCT-VF → OCT-VF	1.24 ± 1.04	0.94 ± 1.22
	OCT-VF → HFA	4.02 ± 2.38	2.65 ± 2.61
	HFA → OCT-VF	3.80 ± 2.13	2.55 ± 2.45
10-2			
	HFA → HFA	2.65 ± 1.60	1.33 ± 1.57
	OCT-VF → OCT-VF	1.34 ± 0.99	1.03 ± 1.18
	OCT-VF → HFA	4.04 ± 2.36	2.52 ± 2.48
	HFA → OCT-VF	3.92 ± 2.29	2.49 ± 2.38

Values are presented as mean ± SD.

* OCT-VF → OCT-VF: Last OCT-VF predicted from prior OCT-VF measurements. HFA → HFA: Last HFA predicted from prior HFA measurements. OCT-VF → HFA: Last HFA predicted from prior OCT-VF measurements. HFA → OCT-VF: Last OCT-VF predicted from prior HFA measurements.

**Figure 4.**
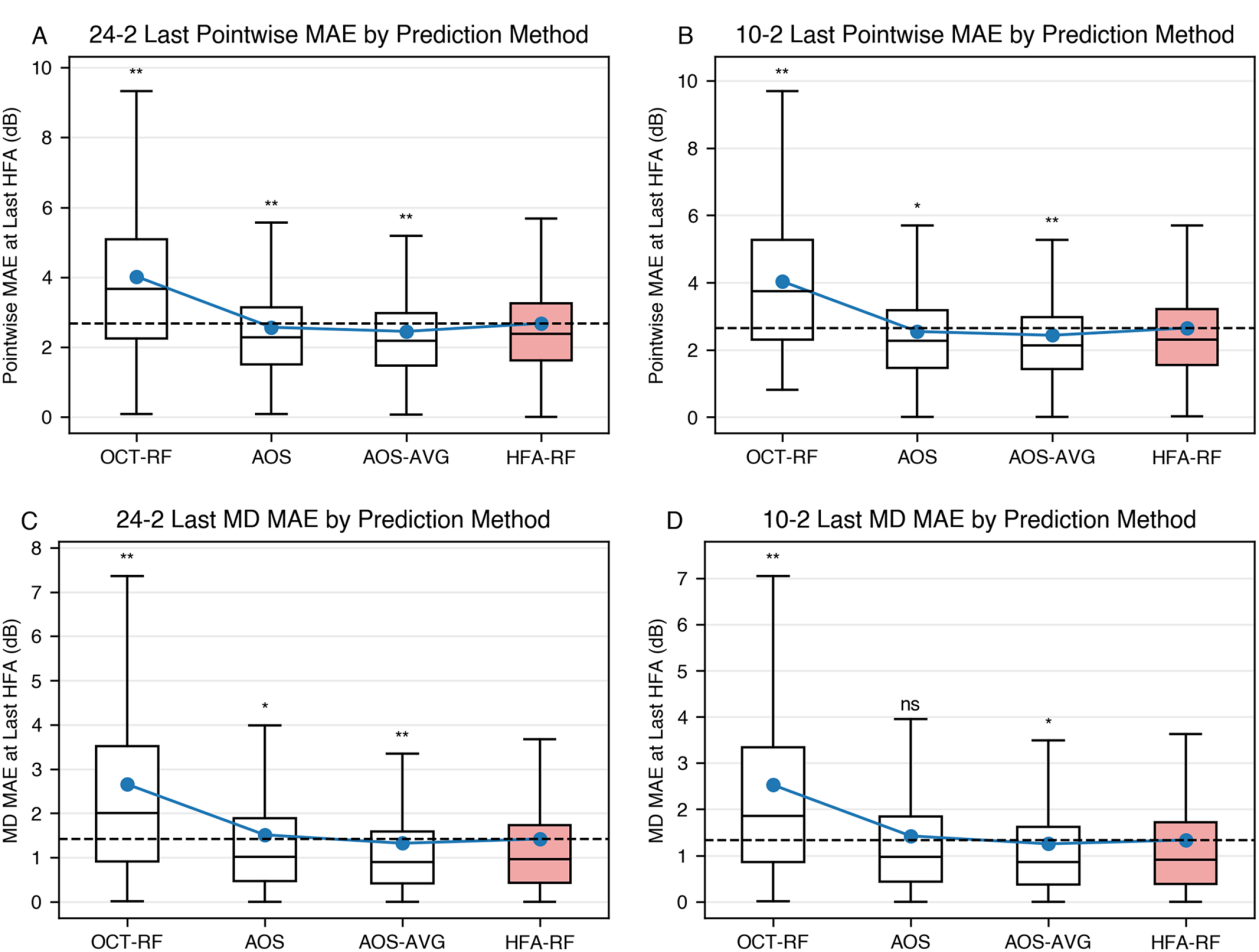
Forecasting the last HFA using OCT-VF and/or HFA histories. (**A**) 24-2 pointwise MAE for the last HFA, (**B**) 10-2 pointwise MAE, (**C**) 24-2 MD MAE, and (**D**) 10-2 MD MAE. Methods compared: OCT-RF (linear trend fitted using all OCT-VF measurements obtained prior to the last HFA and extrapolated to the last HFA timepoint); AOS (HFA-anchored intercept defined by the mean of all prior HFA measurements combined with the OCT-VF–derived slope estimated from measurements prior to the last HFA); AOS-AVG (mean of all HFA tests prior to the last HFA used as the intercept anchor, with the progression rate defined as the arithmetic mean of OCT-VF–derived and HFA–derived slopes estimated from observations prior to the last HFA, used for extrapolation); HFA-RF (linear trend fitted using all prior HFA measurements extrapolated to the last HFA). The AOS approach aims to combine the stable progression rate (slope) derived from OCT-VF with the eye-specific functional intercept provided by HFA. Boxes show the median and interquartile range; whiskers indicate the non-outlier range. Filled circles denote mean MAE; the dashed horizontal line marks the mean MAE of HFA-RF (reference). *Asterisks* above boxes indicate paired comparisons versus HFA-RF, with *P* values from a nonparametric patient-level bootstrap (B = 10,000). Multiplicity was controlled within each endpoint (**A–D**) using the Holm step-down procedure across three pairwise comparisons, comparing OCT-RF, AOS, and AOS-AVG against HFA-RF. Significance codes (display thresholds): **Holm-adjusted *P* < 0.001 (strong evidence); *Holm-adjusted *P* < 0.0125 (Bonferroni-derived threshold used for consistency across analyses); ns not significant at *P* ≥ 0.0125 after Holm adjustment. Lower values indicate better predictions.

Given the higher errors observed in cross-modality forecasting, we next evaluated whether incorporating OCT-VF–derived slope information within an HFA-anchored framework could improve prediction accuracy beyond HFA-only regression for forecasting HFA measurements (Fig. 4; Supplementary Table S1). Across both test patterns and endpoints, the averaged-slope model (AOS-AVG) consistently yielded the lowest MAE, followed by AOS and HFA-RF, whereas OCT-RF showed the highest errors. For pointwise thresholds, AOS-AVG demonstrated significantly lower MAE than HFA-RF in both 24-2 (2.45 vs. 2.68 dB, *P* < 0.001) and 10-2 (2.44 vs. 2.65 dB, *P* < 0.001). AOS also showed modest but statistically significant improvements over HFA-RF in pointwise analysis. For MD, AOS-AVG again showed lower MAE than HFA-RF (24-2: 1.32 vs. 1.42 dB, *P* < 0.001; 10-2: 1.25 vs. 1.33 dB, *P* = 0.002). In contrast, AOS yielded slightly higher MD errors than HFA-RF in 24-2 (1.51 vs. 1.42 dB, *P* = 0.006), whereas no significant difference was observed in 10-2 (*P* = 0.053). OCT-RF, which relies solely on OCT-VF data, showed substantially higher errors across all endpoints.

Analysis of forecast error by disease severity (Supplementary Figs. S3–S5) revealed distinct patterns across methods. For pointwise thresholds, AOS and AOS-AVG generally showed lower absolute and squared errors than both HFA-RF and OCT-RF across disease severity levels, with minor exceptions at the highest and lowest sensitivity ranges. In signed error analysis, HFA-RF, AOS, and AOS-AVG showed largely overlapping trends across severity levels for both pointwise thresholds and MD, indicating minimal systematic bias in the slope-based forecasts. AOS and AOS-AVG closely tracked the error pattern of HFA-RF across most sensitivity ranges, although slightly larger errors were observed at the most advanced disease levels. Under both signed and unsigned error definitions, OCT-RF demonstrated larger variability and systematic deviation at extreme severity ranges.

Spatial analysis of pointwise MAE (Supplementary Fig. S6) showed that AOS-AVG yielded the lowest forecast errors across test locations in both 24-2 and 10-2 patterns, with values comparable to or lower than AOS and uniformly lower than HFA-RF, whereas OCT-RF exhibited the largest errors. Consistent with this observation, severity-stratified analysis by examination count (Supplementary Fig. S7) showed that OCT-RF generally exhibited larger prediction errors across visit strata.

Decomposition of squared errors (Supplementary Figs. S8–S9, Supplementary Table S2) showed that the “Remainder” component (residual component) accounted for the largest share of error (38.4% for 24-2; 40.3% for 10-2) and increased with severity. For the 24-2 pattern, spatial analysis indicated that “Location-fixed” bias was most prominent in the temporal region of the field (corresponding to the optic disc area), whereas “Eye-offset” error was higher in the peripheral field.

### Residual Variability Analysis Using GEEs

Using jackknife resampling, we constructed regression lines for both OCT-VF and HFA measurements separately, and calculated the mean absolute values of the residuals from these lines. Supplementary Table S3 presents the mean absolute residuals and their standard deviations for OCT-VF and HFA datasets. Statistical analysis using GEE showed significantly lower residual variability for OCT-VF compared to HFA across all four analyses: pointwise thresholds (1.09 vs. 2.39 dB for 24-2, 1.19 vs. 2.37 dB for 10-2) and MDs (0.79 vs. 1.13 dB for 24-2, 0.87 vs. 1.07 dB for 10-2), with all *P* < 0.001. This pattern was consistently observed across age groups (Supplementary Fig. S10). Variability stratified by disease severity (Supplementary Fig. S11) showed that pointwise thresholds generally exhibited lower variability with OCT-VF across the dynamic range, with convergence observed near the 0 dB floor, whereas MD variability showed lower values for OCT-VF in mild-to-severe cases but converged and reversed in advanced cases for both 24-2 and 10-2 patterns. Heat maps of residual variability (Supplementary Fig. S12) showed that OCT-VF had lower variability across all test points compared to HFA.

### MD Progression Detection and Rate Analysis

To estimate the false-positive rate under the null hypothesis of no temporal structure, we performed within-eye permutation testing on the longitudinal dataset (Supplementary Table S4). The false-positive rates for detecting MD progression (slope ≤−0.5 dB/year, *P* < 0.025) were 0.00727 for HFA 24-2, 0.00560 for OCT-VF 24-2, 0.00750 for HFA 10-2, and 0.00589 for OCT-VF 10-2, all below the nominal 2.5% threshold.

Using HFA as the reference standard (Supplementary Table S5), OCT-VF progression detection showed sensitivities of 0.461 (24-2) and 0.396 (10-2), with specificities of 0.917 (24-2) and 0.901 (10-2). Cohen's kappa values were 0.393 (24-2) and 0.312 (10-2). Detailed confusion matrices are provided in Supplementary Table S6.

**Figure 5.**
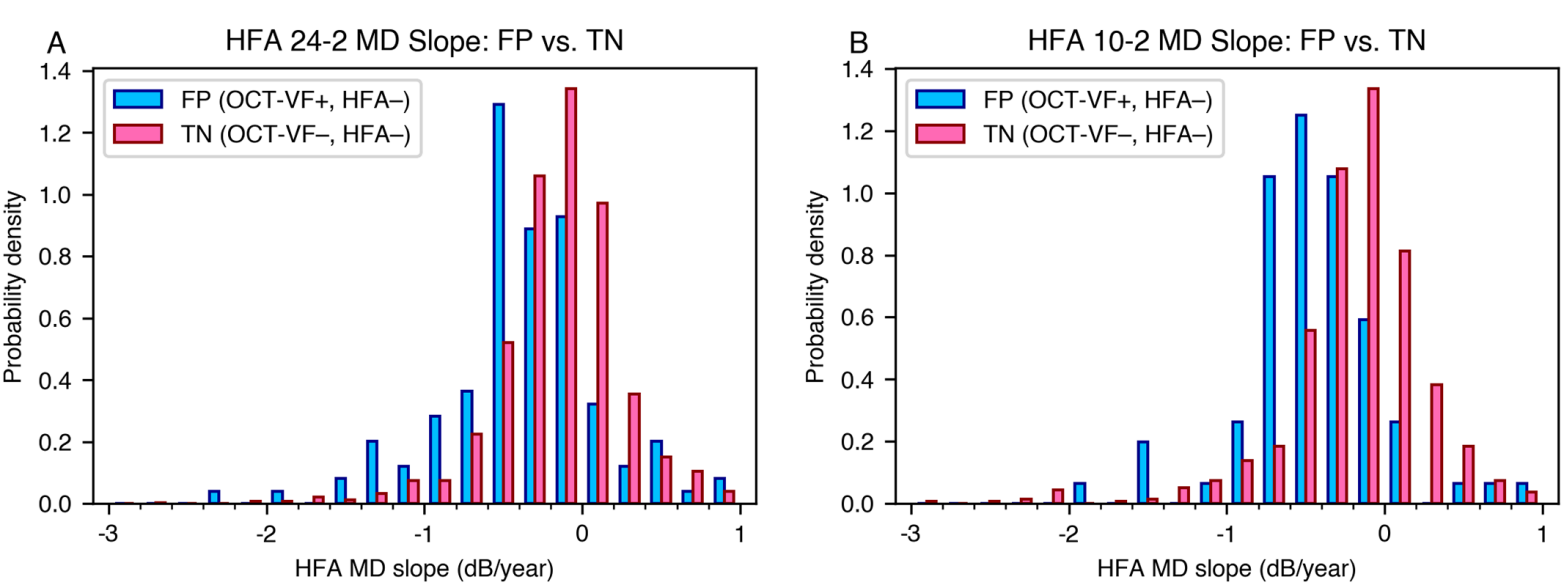
HFA MD slopes for FP and TN eyes. FP and TN eyes were defined using the same progression criteria (MD slope ≤ −0.5 dB/year and *P* < 0.025) with HFA as the reference standard; OCT-VF progression was evaluated using identical thresholds. FP corresponded to OCT-VF+, HFA–, and TN to OCT-VF–, HFA– (see Supplementary Table S6 for full confusion matrices). Panels **A** (24-2) and **B** (10-2) display probability density histograms of HFA MD slopes for FP eyes (*n* = 125 for 24-2, *n* = 77 for 10-2) and TN eyes (*n* = 1380 for 24-2, *n* = 698 for 10-2). FP eyes showed significantly more negative MD slopes, indicating faster functional deterioration that did not satisfy HFA's progression threshold despite being detected by OCT-VF. All between-group differences were statistically significant (all *P* < 0.001; see Supplementary Table S8).

To further characterize eyes in which OCT-VF detected progression despite non-significant findings on HFA, we compared false-positive (FP) and true-negative (TN) eyes using HFA as the reference standard. FP eyes were defined as eyes showing OCT-VF–detected progression but not meeting HFA significance criteria (OCT-VF+/HFA–), and TN eyes as OCT-VF– / HFA–, based on identical progression criteria (MD slope ≤–0.5 dB/year and *P* < 0.025). Across both test patterns, FP eyes exhibited significantly greater HFA MD residual variability, reflected by larger absolute residuals, than TN eyes (24-2: 1.51 vs. 1.08 dB; 10-2: 1.30 vs. 1.05 dB; both *P* < 0.001; Supplementary Table S7). Density plots of MD residuals for FP and TN eyes are provided in Supplementary Figure S13. FP eyes also demonstrated significantly more negative HFA MD slopes than TN eyes (24-2: −0.412 vs. −0.147 dB/year; 10-2: −0.437 vs. −0.183 dB/year; both *P* < 0.001; Supplementary Table S8). Corresponding slope distributions are illustrated in Figure 5. Among eyes identified as progressing by both modalities, no statistically significant difference was observed in the time required to detect MD progression between OCT-VF and HFA for either test pattern (Supplementary Fig. S14; Supplementary Table S9).

MD progression slopes showed correlations between OCT-VF and HFA (Supplementary Fig. S15). Passing-Bablok regression^35^ slopes ranged from 0.663 to 1.031 across test patterns and analysis subsets. When the axes were reversed and ordinary least-squares regression was applied (Supplementary Fig. S16), slopes ranged from 0.567 to 0.851. Bland-Altman analysis (Supplementary Fig. S17) showed minimal systematic bias for all eyes, with mean differences in MD slopes (OCT-VF minus HFA) of 0.01 dB/year for 24-2 and 0.03 dB/year for 10-2. Analysis of slope error versus MD (Supplementary Fig. S18) demonstrated no systematic relationship between severity and slope differences, with Passing-Bablok regression slopes near zero across all severity levels (all 95% confidence intervals [CIs] included zero), indicating that OCT-VF showed no systematic bias across the dynamic range of visual field damage.

Comparison of mean MD slopes between modalities for all eyes (Supplementary Table S10) showed no statistically significant differences between OCT-VF and HFA. For the 24-2 pattern, mean slopes were −0.316 dB/year (OCT-VF) and −0.325 dB/year (HFA), with a mean difference (Δ) of 0.010 dB/year (95% CI: −0.023 to 0.043; *P* = 0.557). For the 10-2 pattern, mean slopes were −0.359 and −0.393 dB/year, respectively (Δ = 0.034 dB/year; 95% CI: −0.017 to 0.083; *P* = 0.185). These findings indicate no systematic difference in estimated progression rates between modalities.

### Comparison With Macular GCC Progression

We performed a subanalysis on a single-center dataset of eyes where longitudinal macular GCC thickness measurements were available to compare the progression detection performance of OCT-VF against standard structural metrics (Supplementary Fig. S1). This dataset included 1078 eyes (24-2) and 402 eyes (10-2) (Supplementary Table S11). Within-eye permutation testing showed false-positive rates below the nominal 2.5% threshold for progression detection based on both MD and GCC slopes (Supplementary Table S12).

Given that our 9 × 9 mm macular OCT scan area extends nasally to approximately the optic disc center and thereby captures the retinal ganglion cell axon pathways corresponding to central VF locations, we prioritized the analysis of the 10-2 test pattern. Using HFA as the reference standard, OCT-VF demonstrated better agreement in progression detection compared to GCC across all threshold definitions (Supplementary Tables S13–S14). For 10-2 with the −0.5 µm/year GCC threshold, which substantially exceeds the reported rate of normal age-related GCC thinning (approximately −0.17 µm/year),^36^ OCT-VF showed sensitivity of 0.753, specificity of 0.875, and Cohen's κ of 0.560, whereas GCC showed sensitivity of 0.370, specificity of 0.690, and Cohen's κ of 0.046. The difference in Cohen's κ between OCT-VF and GCC was 0.512 (95% CI, 0.372 to 0.647; *P* < 0.001), indicating significantly better agreement for OCT-VF. Similar patterns were observed for 24-2 and across different GCC thresholds.

Venn diagram analysis (Supplementary Figs. S19–S21) revealed that the choice of GCC threshold influenced progression detection characteristics. At the <0.0 µm/year threshold, GCC demonstrated elevated false-positive rates, whereas the <−1.0 µm/year threshold resulted in reduced sensitivity. For 10-2 at the intermediate −0.5 µm/year threshold, GCC performance was inferior to OCT-VF for both true positives (27 versus 55 eyes) and false positives (102 versus 41 eyes), demonstrating OCT-VF's better specificity while maintaining higher sensitivity. A similar pattern was observed in the 24-2 analysis.

Finally, we evaluated the longitudinal agreement between the rates of change in HFA MD and those derived from OCT-VF and GCC (Supplementary Table S15). OCT-VF MD slopes showed significantly stronger correlations with HFA MD slopes than GCC thickness slopes. For the 10-2 pattern, Pearson's *r* was 0.850 for OCT-VF compared with 0.290 for GCC (Δ*r* = 0.549; 95% CI, 0.351 to 0.772; *P* < 0.001). Similar results were observed for the 24-2 pattern and when using Spearman's rank correlation. Disease severity–stratified analyses further showed that OCT-VF maintained stronger associations with HFA MD slopes than GCC across severity levels (Supplementary Table S16). For the 10-2 pattern, Pearson's *r* for OCT-VF ranged from 0.732 to 0.866 across severity strata, whereas GCC correlations ranged from 0.189 to 0.540. Scatterplots with Passing–Bablok regression (Supplementary Figs. S22, S23) provided visual confirmation that OCT-VF progression rates aligned more closely with HFA MD slopes, whereas GCC slopes exhibited weaker correspondence with functional decline.

### Agreement Between BAE and OCT-VF

**Figure 6.**
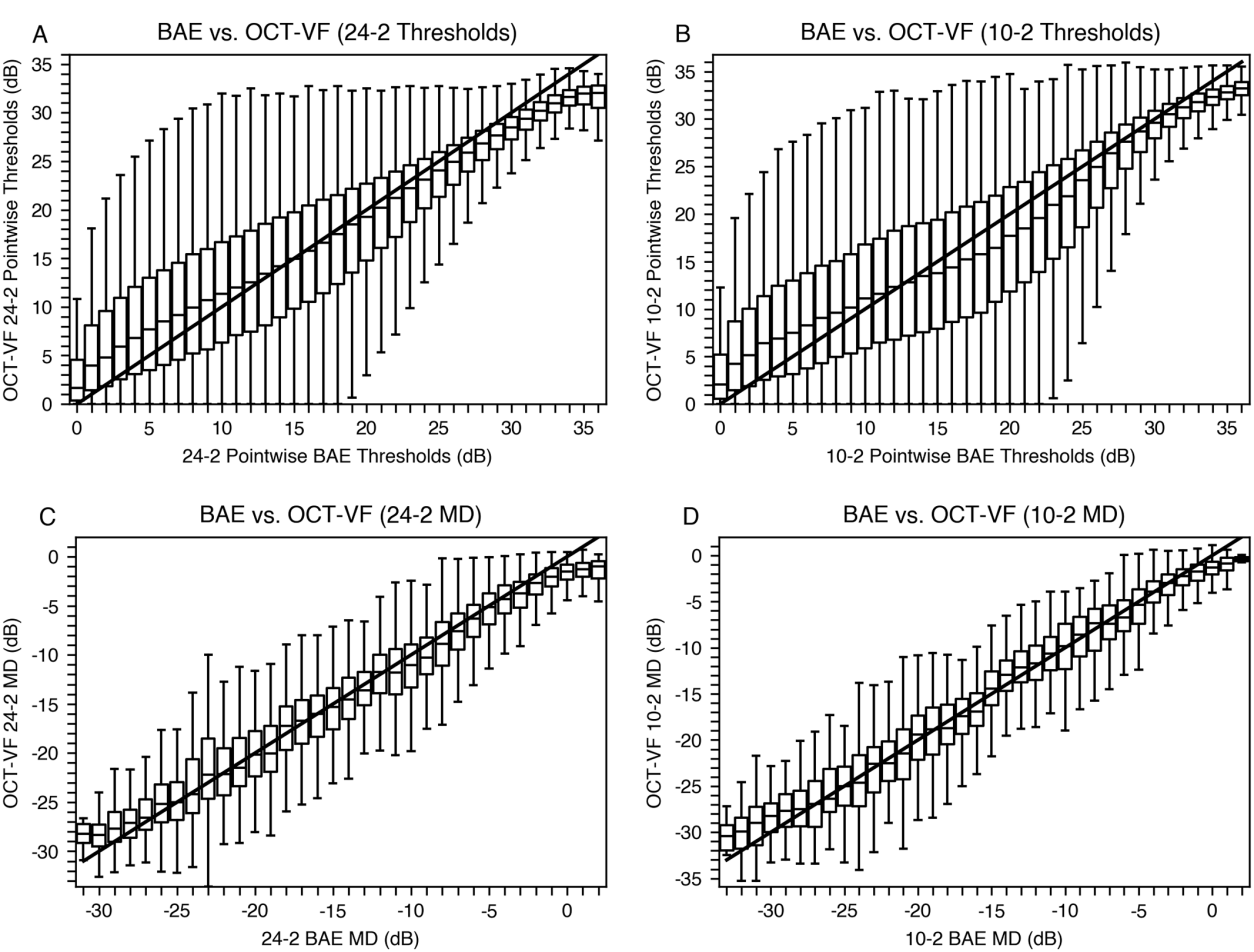
Correlations between BAE and OCT-VF parameters. (**A**) 24-2 pointwise thresholds, (**B**) 10-2 pointwise thresholds, (**C**) 24-2 MD, and (**D**) 10-2 MD. Each panel displays boxplots of OCT-VF estimates for each corresponding BAE value, along with the identity line (x = y). BAE values were derived from linear regression over time for each eye. For pointwise thresholds, a separate regression line was fitted for each test location, and the predicted HFA value at each OCT scan date was used as the BAE threshold. For MD, a regression line was fitted using all longitudinal HFA measurements for each eye, and the predicted MD at each OCT scan date was used as the BAE MD. BAE values represent smoothed estimates derived from the longitudinal HFA series and are used as a reference for comparison rather than as independent measurements. OCT-VF values were generated for all available OCT scans in the longitudinal dataset, and each prediction was paired with the corresponding BAE value obtained at that same OCT date.

Supplementary Table S17 shows the agreement between OCT-VF and the BAE for each parameter. The mean absolute error (MAE) between OCT-VF and BAE was 3.33 dB for 24-2 pointwise thresholds, 3.47 dB for 10-2 pointwise thresholds, 2.27 dB for 24-2 MD, and 2.25 dB for 10-2 MD. Figure 6 shows the relationship between OCT-VF and the corresponding BAE values for both pointwise thresholds and MDs. Analysis of the absolute error relative to BAE (Supplementary Fig. S24) revealed that absolute errors showed increasing trends in mild to moderate cases, followed by a reduction in the most advanced range, resulting in an inverted-U–shaped pattern. Furthermore, analysis of the error (OCT-VF minus BAE) relative to BAE (Supplementary Fig. S25) showed a shrinkage toward the central tendency; OCT-VF tended to overestimate more severe (lower) thresholds and underestimate better (higher) thresholds. The Bland-Altman analysis showed absolute mean differences between OCT-VF and BAE values of 0.6 dB or less across all analyses (Supplementary Fig. S26). The model's estimation accuracy remained relatively stable across various OCT focus values; regression slopes were close to zero and 95% confidence intervals included zero for most analyses, indicating minimal impact of refractive errors on performance (Supplementary Fig. S27). The spatial distribution of MAE indicated lower error rates in nasal VF locations in severe cases (Supplementary Figs. S28, S29). When the same trained model was evaluated in the GCC analysis dataset, the observed MAE values between OCT-VF and BAE were numerically smaller (MAE: 2.05 dB for 24-2 thresholds, 2.79 dB for 10-2 thresholds, 1.39 dB for 24-2 MD, and 1.78 dB for 10-2 MD) than those in the full longitudinal cohort (Supplementary Tables S17, S18).

### Analysis of Visual Field Measurements at the Lower Threshold Limit

Supplementary Figure S30 shows that OCT-VF produced fewer measurements clustered at 0 dB compared to BAE, with OCT-VF estimates typically remaining slightly above the threshold floor. To further explore this discrepancy, longitudinal analysis (Supplementary Fig. S31) showed that HFA measurements were more likely to remain at 0 dB over time. In contrast, OCT-VF estimates tended to fluctuate slightly above the threshold floor. When recovery from 0 dB did occur, HFA values exhibited large and variable increases (24-2: 10.5 ± 7.1 dB, 10-2: 10.4 ± 7.4 dB), while OCT-VF changes were small and stable (24-2: 1.7 ± 1.6 dB, 10-2: 2.0 ± 1.8 dB).

## Discussion

Our forecasting analyses provide several insights into the longitudinal behavior of OCT-derived visual field representations. As expected, direct cross-modality forecasting—such as predicting future HFA values using OCT-VF measurements alone—resulted in substantially higher errors than within-modality predictions, indicating that OCT-derived representations and functional perimetric measurements retain modality-specific variability and are not directly interchangeable at the individual level. However, integrating both modalities within a hybrid framework significantly improved prediction accuracy. In particular, the averaged-slope model (AOS-AVG), which combines an HFA-anchored intercept with the mean of OCT-VF–derived and HFA-derived longitudinal slopes, consistently yielded the lowest forecasting errors. One possible explanation is that averaging slope estimates derived from two different modalities may mitigate modality-specific noise, thereby producing a more stable and accurate estimate of longitudinal change. These findings suggest that OCT-derived information may provide complementary longitudinal signals beyond those captured by perimetry alone, and that integrating slope estimates from both modalities can further stabilize forecasting of future visual-field outcomes.

The performance of the AOS framework provides further insight into the mechanism underlying this hybrid forecasting approach. By combining an HFA-anchored intercept with an OCT-VF–derived slope, this framework separates the roles of functional level anchoring and trend estimation. HFA measurements remain essential for anchoring the individual functional level, whereas OCT-VF provides slope information that reflects structure-derived functional change with lower longitudinal variability. This interpretation is consistent with our error decomposition analysis, which identified systematic components such as eye-offset errors and location-fixed errors that can be mitigated through intercept anchoring strategies. Interestingly, although the basic AOS model showed no advantage (and even slightly higher error) for MD forecasting, the AOS-AVG model demonstrated significant improvements. This indicates that even for spatially averaged indices such as MD, which already exhibit inherently reduced variance, incorporating slopes derived from OCT-based functional estimates provides a stabilizing effect against perimetric noise.

Furthermore, the forecasting experiments revealed that prediction difficulty differed depending on the prediction target (OCT-VF vs. HFA). Predictions targeting OCT-VF generally showed smaller errors than those targeting HFA. This difference likely reflects the larger test–retest variability of standard automated perimetry compared with OCT-VF–derived representations. Because OCT-VF represents a structure-derived functional estimate with less variability, forecasts may be more stable when OCT-VF is used as the prediction target. In contrast, the larger variability of perimetric measurements increases uncertainty in model estimation, which may amplify forecasting errors when HFA is used as the prediction target.

More fundamentally, direct cross-modality prediction—targeting HFA using only OCT-VF estimates—is inherently challenging because each modality contains modality-specific sources of variability. On the HFA side, measurements are influenced by patient-specific response behaviors (response bias), which were identified as "eye-offset" errors (individual intercept bias) in our decomposition analysis. Conversely, OCT-VF estimates are algorithmically generated and may be subject to case-specific estimation biases or a model-driven tendency to regress toward central tendencies. These findings suggest that although HFA remains the clinical reference standard, its measurement noise represents an inherent limitation for forecasting, one that may be partially mitigated by incorporating the relatively stable signal provided by OCT-VF.

Our analysis revealed important considerations when estimating visual field measurements near the lower threshold limit. OCT-VF produced fewer measurements at 0 dB compared to the BAE derived from HFA, and longitudinal analysis showed that OCT-VF estimates tended to fluctuate slightly above the floor with smaller variance than HFA. This tendency to underestimate severe damage likely stems from mean squared error-based training, which inherently encourages predictions toward central tendencies rather than extreme floor values. Consistent with this floor behavior, analysis of severity-stratified forecasting errors showed a subtle tendency toward larger signed errors for AOS than HFA-RF at test locations with observed thresholds of 0 dB. This pattern may reflect the tendency of OCT-VF estimates to remain slightly above the absolute floor when the functional sensitivity is already at 0 dB. An additional contributing factor may be the increased measurement variability known to occur at low sensitivity levels in standard automated perimetry.^4^^,^^37^ This increased variability in the lower dynamic range elevates the likelihood of perimetric measurements being recorded at the threshold floor, a phenomenon that algorithmically derived estimates such as OCT-VF may inherently smooth over.

The better forecasting performance of the hybrid approach can be attributed, at least in part, to the lower measurement variability of OCT-VF. Jackknife resampling and GEE analysis demonstrated significantly lower residual variability for OCT-VF across both pointwise thresholds and MD values, with this advantage persisting across regions and age groups. A potential concern with neural network-based representations is that models trained on paired OCT–HFA data may regress predictions toward central tendencies rather than faithfully tracking longitudinal change. Because OCT-VF predictions were generated independently for each visit without explicit temporal smoothing, such regression effects would be expected to manifest as attenuation of estimated slopes. Our analysis directly addressed this possibility: comparison of mean MD slopes between OCT-VF and HFA showed no statistically significant differences across test patterns or analysis subsets, with bootstrap confidence intervals for Δ MD slope consistently spanning zero. Furthermore, no significant relationship was observed between HFA MD and Δ MD slope, as Passing–Bablok confidence intervals consistently included zero. Together, these results provide no evidence that OCT-VF progression estimates systematically attenuate longitudinal change or introduce severity-dependent bias. Although Passing–Bablok regression comparing MD progression slopes between HFA and OCT-VF yielded slopes below unity, this pattern is expected under asymmetric measurement variability. Greater dispersion in HFA MD slopes increases variance along the predictor axis, which can mechanically reduce regression slopes without implying biological or algorithmic flattening. Importantly, reversing the axes and applying ordinary least-squares regression yielded similarly sub-unity slopes, confirming that slope magnitude is driven by the variance structure of the two measurements rather than by modality-specific attenuation.

In the present study, slope analyses focused on global MD trends. Direct pointwise slope comparisons were not performed because location-specific regression estimates exhibited substantially greater variability than global indices, limiting the stability and interpretability of slope-based statistical comparisons. Consequently, although no systematic bias was observed at the MD level, localized differences in pointwise slope behavior cannot be entirely excluded. Nevertheless, the forecasting analyses provide indirect context for interpreting this limitation. Although signed pointwise prediction errors were broadly similar between HFA-RF and AOS across disease severity levels, absolute prediction errors tended to be smaller for AOS across most sensitivity ranges. This behavior is compatible with OCT-VF–derived slopes capturing longitudinal variation relevant to pointwise forecasting without introducing large systematic distortions.

In progression detection analyses, eyes classified as OCT-VF positive but HFA negative showed significantly greater perimetric variability and more negative HFA MD slopes than true-negative eyes. These findings suggest that at least some discordant classifications may represent eyes with underlying declining tendencies that remain undetected by conventional perimetric progression criteria due to measurement variability. Increased variability reduces the statistical power of perimetric progression tests, making it more difficult for true declining trends to exceed significance thresholds. Although structural–functional dissociation remains a possible explanation, the observed pattern is also compatible with OCT-VF capturing progression-related signals that are obscured by variability in perimetric measurements. Consequently, some cases classified as OCT-VF false positives relative to HFA may instead reflect limitations in the sensitivity of perimetric progression detection. This interpretation may also explain why eyes classified as progressing by both modalities did not show earlier detection by OCT-VF, as such eyes may represent cases with sufficiently strong perimetric progression signals to be detected by conventional tests.

The comparison with conventional structural metrics suggested that OCT-VF may offer advantages for monitoring concurrent disease progression. In our subset analysis, OCT-VF progression rates showed stronger alignment with functional decline (HFA MD slopes) than macular GCC thickness slopes across disease severities, whereas GCC thinning demonstrated only modest concordance with central visual field change. Several methodological features may help explain this difference. First, conventional structural metrics reduce the macular volume to a limited set of thickness summaries across retinal layers, whereas the 3DCNN leverages the full volumetric information from the entire macular cube, potentially capturing spatial patterns that are not expressed in aggregated thickness values. Second, because GCC thickness is susceptible to floor effects, OCT-VF appears to preserve useful structure–function signal further into advanced disease. Third, OCT-VF is trained directly to predict functional patterns from paired datasets rather than relying solely on anatomical correlation. These results suggest that volumetric deep learning extracts progression signals that are less apparent in thickness-based metrics, while complementing—rather than replacing—conventional structural OCT measures in longitudinal monitoring.

Direct comparison of MAE or RMSE values across published OCT–VF studies requires caution because both the reference standard and the underlying case mix differ substantially between cohorts. In our study, we used regression-based BAE rather than single-timepoint HFA measurements as the reference standard, motivated by the increase in perimetric variability at low sensitivities.^4^^,^^37^ Regression-based estimates leverage routinely collected longitudinal data to provide more stable reference values across the full dynamic range of disease severity, without requiring repeat perimetry within short intervals or discarding severely damaged test locations.

These methodological choices, together with cohort composition, complicate cross-study interpretation of global MAE. For example, when we applied the same trained model to two different cohorts, the GCC analysis subset—enriched with milder disease—yielded lower MAE than the more heterogeneous longitudinal dataset, despite identical architecture and weights. Populations enriched with mild disease naturally produce lower error values, whereas regression-based references reduce apparent error by attenuating short-term noise and smoothing low sensitivities. Thus, lower MAE does not necessarily imply a more accurate model, and higher MAE does not necessarily indicate inferior performance. Consistent with this, we observed an inverted-U pattern in error relative to BAE—peaking in moderate stages and decreasing again in the most advanced range—contrasting with studies that used single raw HFA values as ground truth.^16^^,^^19^^,^^20^^,^^22^^,^^23^^,^^25^ Our model also differs from most prior work in being trained on a heterogeneous, multi-condition population rather than glaucoma-specific cohorts.^12^^–^^25^ When considered together—reference standard, severity distribution, and training population—a simple numerical comparison of global MAE across studies risks attributing differences driven by methodology to model superiority or inferiority. Severity-stratified reporting and standardized evaluation frameworks will be essential for meaningful cross-study comparisons going forward.

This study has several limitations. First, discordance between OCT-VF and HFA may arise from model inaccuracies, inherent perimetric noise, or true structural–functional dissociation. Second, analyses were restricted to a single OCT platform with a 9 × 9 mm macular scan protocol; generalizability to other devices or acquisition settings requires further validation. Third, residual learning effects and imperfect temporal alignment between modalities may have added noise to the longitudinal analyses. Finally, the strictly matched longitudinal conditions used in this study—designed to ensure a rigorous comparison by equalizing measurement counts and matching follow-up periods—may not fully reflect routine clinical practice, in which OCT is often acquired more frequently than perimetry.

Future research should address these limitations through external validation, prospective longitudinal studies, and broader functional outcomes. Validation across diverse populations, OCT platforms, and clinical settings will be essential to establish generalizability. Prospective studies with standardized follow-up may clarify the temporal relationship between structural and functional change and determine whether improvements in forecasting behavior translate to clinically meaningful benefits. In addition, evaluation of multimodal monitoring strategies and case-specific clinical decision frameworks, together with systematic testing across different OCT hardware, will be important for defining where OCT-VF offers the greatest added value and how it can be safely implemented. Notably, in routine follow-up, structural imaging is typically performed more frequently than perimetry owing to its shorter acquisition time and minimal reliance on patient engagement. Furthermore, whereas perimetric monitoring commonly requires exclusion of initial tests to mitigate learning effects, OCT-derived representations can be utilized from the first visit. These practical differences suggest that the complementary value of OCT-VF—including progression detection characteristics and forecasting stability under higher sampling density—may differ from that observed under the strictly matched analytical conditions of the present study. Prospective head-to-head comparisons preserving real-world sampling patterns will be necessary to quantify these potential effects.

## Conclusions

A hybrid forecasting framework combining OCT-VF–derived slope information with an HFA-based functional intercept improved longitudinal visual-field prediction, with the averaged-slope model (AOS-AVG) achieving the lowest forecasting errors across endpoints. OCT-VF–derived slopes provided complementary progression information without introducing systematic bias at the global MD level and exhibited lower longitudinal variability than perimetric measurements. In addition, OCT-VF showed the potential to highlight progression-related signals in some eyes where high perimetric variability limited conventional progression detection. These findings suggest that structure-derived functional representations may complement standard perimetry for longitudinal monitoring. Further prospective studies are needed to determine how such hybrid approaches influence clinical decision-making and patient outcomes.

## Supplementary Material

Supplement 1
