# Supplementary material for: Multicenter OCT-Based Visual Field Representations via Segmentation-Free 3D CNNs: Forecasting, Longitudinal Variability, and Progression Detection
Source: Transl Vis Sci Technol. 2026 Jul 13;15(7):18. doi: 10.1167/tvst.15.7.18 (PMC13374838; doi:10.1167/tvst.15.7.18)
Supplement: Supplement 1 [file tvst-15-7-18_s001.pdf]

Supplementary Methods

Error decomposition of the last HFA forecasts

We decomposed the forecast error for the last HFA into eye-level, location-level, and remainder components using squared errors. For eye i and test location p, let $T_{i,p}^{\mathrm{OCT}}$ and $T_{i,p}^{\mathrm{HFA}}$ denote the OCT-RF forecast and the observed last HFA threshold, respectively. Let $T_{i,p}^{\mathrm{AOS}}$ be the AOS forecast at the same timepoint. Mean deviation (MD) for each modality at the last HFA timepoint is denoted M_i^{OCT}, M_i^{HFA}, and M_i^{AOS}. The total squared error is

$$e_{\mathrm{tot},i,p} = (T_{i,p}^{\mathrm{OCT}} - T_{i,p}^{\mathrm{HFA}})^2.$$

To remove eye-level offsets, we compared thresholds after centering each modality by its own MD:

$$e_{\mathrm{MDoff},i,p} = [(T_{i,p}^{\mathrm{OCT}} - M_i^{\mathrm{OCT}}) - (T_{i,p}^{\mathrm{HFA}} - M_i^{\mathrm{HFA}})]^2.$$

To further remove location-fixed bias, we anchored the intercept with HFA and used the OCT-derived slope (AOS) and again compared centered thresholds:

$$e_{\mathrm{rem},i,p} = [(T_{i,p}^{\mathrm{AOS}} - M_i^{\mathrm{AOS}}) - (T_{i,p}^{\mathrm{HFA}} - M_i^{\mathrm{HFA}})]^2.$$

For each 1-dB bin b of the observed last HFA threshold, we averaged these quantities over all valid (i, p) in the bin using identical eye–location sets. The component mean squared errors were then defined as

$$\bar{E}_{\mathrm{Eye\text{-}offset}}(b) = \bar{e_{\mathrm{tot}}}(b) - \bar{e_{\mathrm{MDoff}}}(b),$$

$$\bar{E}_{\mathrm{Location\text{-}fixed}}(b) = \bar{e_{\mathrm{MDoff}}}(b) - \bar{e_{\mathrm{rem}}}(b),$$

$$\bar{E}_{\mathrm{Remainder}}(b) = \bar{e_{\mathrm{rem}}}(b),$$

with $\bar{E}_{\mathrm{Total}}(b) = \bar{e_{\mathrm{tot}}}(b)$. We display the absolute component magnitudes as stacked bars so that changes in error level with disease severity are visible. 95% confidence intervals were obtained by patient-level bootstrap resampling (B = 10,000) using paired resamples across modalities. For each grid location, we computed the location-wise means of the component squared errors (dB^2) across eyes and displayed them as heatmaps for 24-2 and 10-2.

Statistical Analysis for Longitudinal Variability

We assessed residual variability using jackknife resampling, applied separately to the OCT-VF and HFA test datasets. This statistical technique works as follows: for each eye's time series in each dataset, we systematically removed one data point at a time, fitted a linear regression line to the remaining data points, and then calculated the absolute difference between the predicted value from this regression line and the actual measured value at the excluded time point (absolute residual). This process was repeated for every data point in each eye's time series. The jackknife technique was selected for its robustness to outliers and suitability for dependent longitudinal measurements, as it provides an approximately unbiased estimate of how well each individual measurement conforms to the overall temporal trend while reducing bias arising from differences in sampling density between eyes. We compared OCT-VF and HFA datasets using generalized estimating equations (GEE), a framework for longitudinal data analysis that accounts for within-subject correlation (Liang & Zeger, *Biometrika*, 1986), adjusting for follow-up duration, the number of longitudinal examinations per eye, age, clustering by eye and patient, and measurement point location for pointwise threshold analyses only. To account for multiple comparisons across the four variability metrics (24-2 pointwise thresholds, 24-2 MD, 10-2 pointwise thresholds, and 10-2 MD), we applied Bonferroni correction with an adjusted significance threshold of $P < 0.0125$ ($\alpha = 0.05/4$).

To examine how residual variability varied across disease severity, we stratified measurements by visual field severity using the mean of the OCT-VF and time-matched HFA values for each measurement. We also visualized spatial patterns of residual variability across test locations for both modalities. Additionally, we examined the relationship between residual variability and patient age by stratifying measurements into 5-year age groups.

MD Progression Detection and Rate Comparison

In addition to forecasting analysis, we evaluated progression detection using the full longitudinal dataset. We performed linear regression analysis on MD values over time for both 24-2 and 10-2 test patterns, applied separately to the OCT-VF and HFA datasets. We defined significant progression as cases with a regression slope ≤–0.5 dB/year and a statistically significant P value ($P <$ 0.025 after Bonferroni correction for multiple comparisons across the two test patterns). This slope

threshold is consistent with prior work that classified visual field loss faster than −0.5 dB/year as clinically meaningful rapid progression (Zhang et al., *Am J Ophthalmol*, 2019). To estimate the false-positive rate under the null hypothesis of no temporal structure, we performed within-eye permutation testing on the full longitudinal dataset by randomly shuffling the measurement order within each eye 1,000 times and reapplying the same progression criteria.

To assess agreement in progression detection between OCT-VF and HFA, we used HFA as the reference standard and constructed 2×2 confusion matrices for both test patterns. Eyes were classified as progressing or non-progressing based on whether they met the progression criteria in each modality. From these confusion matrices, we calculated sensitivity, specificity, accuracy, and Cohen's kappa coefficient to quantify the diagnostic performance of OCT-VF relative to HFA.

To further investigate modality discordance, we compared false-positive (FP) and true-negative (TN) eyes. Using HFA as the reference standard, FP eyes were defined as OCT-VF–positive but HFA–negative for progression (OCT-VF+ / HFA–), whereas TN eyes were negative in both modalities (OCT-VF– / HFA–). For these two groups, we compared HFA–derived MD slopes and longitudinal residual variability (estimated using jackknife resampling) using the Mann–Whitney U test to determine whether FP eyes exhibited more negative slopes or greater HFA variability than TN eyes.

Among eyes showing significant progression in both modalities (concurrent progressors), we compared the time required to reach statistical significance for each modality. Differences in detection time between OCT-VF and HFA were evaluated using patient-level paired bootstrap resampling (10,000 iterations).

To compare the rates of progression (MD slopes) between OCT-VF and HFA, we used Passing–Bablok regression, a nonparametric method that accounts for measurement error in both modalities (Passing & Bablok, *J Clin Chem Clin Biochem*, 1983). Additionally, we assessed the difference in mean MD slopes between methods using patient-wise bootstrap resampling (10,000 iterations). We also constructed scatterplots with reversed axis assignments and applied ordinary least-squares regression to visualize slope behavior under conventional regression assumptions. To evaluate systematic bias in progression rates between modalities, Bland-Altman analysis was performed. Furthermore, to assess whether OCT-VF showed systematic bias across the dynamic range of visual

field damage, we analyzed slope error versus MD and applied Passing-Bablok regression to examine the relationship between severity and slope differences.

GCC Dataset Analysis

We evaluated macular ganglion cell complex (GCC) progression in a single-center dataset. Because automated GCC segmentation data were not systematically archived across all participating institutions, we retrospectively curated GCC thickness measurements at Minamikoyasu Eye Clinic. The GCC analysis dataset was constructed using the same longitudinal pairing and trimming procedures as the main longitudinal test dataset, except that no restriction was applied to HFA test strategy. This was because all HFA examinations at Minamikoyasu Eye Clinic were performed using SITA-Fast, making a SITA-Standard–only constraint inapplicable. The OCT-based visual field (OCT-VF) and GCC analyses were computed from the same OCT acquisitions per visit; therefore, the OCT-VF and GCC test counts were identical within this dataset. For visits where multiple OCT scans were acquired on the same day, GCC thickness was derived from a single scan selected via visual inspection by a board-certified ophthalmologist specializing in glaucoma (M.K.) to minimize segmentation errors, whereas OCT-VF values were computed as SSI-squared weighted averages as described above. GCC thickness was calculated as the average of the superior and inferior hemisphere measurements. The same author retrospectively reviewed the medical records and excluded eyes with any non-glaucomatous ocular pathology or with evident segmentation failure on visual inspection. Commonly excluded conditions included age-related macular degeneration, diabetic retinopathy, and epiretinal membrane.

For each eye, we estimated MD slopes from HFA and from OCT-VF, and GCC thickness slopes from OCT, using simple linear regression against test date. Statistical significance of a negative trend was assessed with two-sided tests. Following our familywise control across the two test patterns (24-2 and 10-2), significance for MD slopes was defined as slope ≤–0.5 dB/year with $P < 0.025$ for both HFA and OCT-VF. Because units differ for GCC, we evaluated three predefined thresholds—GCC slope <0.0, <–0.5, and <–1.0 μm/year—each with $P < 0.025$. Device-specific adjustment factors for age-related thinning and signal strength index (SSI) effects have not been established for the Nidek RS-3000 platform and were therefore not applied to GCC slope estimation. Instead, we sought to

mitigate potential bias from age-related change and image-quality variation through two approaches: (1) quality control (SSI ≥7) to minimize SSI-related variability and (2) evaluation of progression using predefined slope thresholds (<−0.5 and <−1.0 μm/year) that substantially exceed reported rates of normal age-related GCC thinning (approximately −0.17 μm/year; Zhang et al., *Transl Vis Sci Technol*, 2016). False-positive rates for these progression criteria were estimated by within-eye permutation testing, in which measurement order was randomly shuffled 1,000 times per eye, and the same regression-based rules were reapplied.

Agreement analyses were performed at the eye level. Using HFA as the reference standard, we derived confusion matrices and assessed the diagnostic performance of OCT-VF and, separately, of GCC at each threshold (sensitivity, specificity, accuracy, and Cohen's κ). To compare OCT-VF with GCC directly while preserving paired sampling, we reported Δ Cohen's κ = κ(OCT-VF) – κ(GCC) with 95% confidence intervals from patient-level paired bootstrap resampling (B = 10,000); bootstrap *P* values were provided for Δ. Overlap of progression calls across HFA, OCT-VF, and GCC was summarized with Venn diagrams using the foregoing definitions. To visualize association, we plotted scatterplots of HFA MD slope versus GCC thickness slope with Passing–Bablok fits; Pearson's *r* was reported for descriptive correlation. In addition, in the GCC analysis dataset including all eyes, we compared Pearson's and Spearman's correlations between GCC slope and HFA MD slope versus between OCT-VF MD slope and HFA MD slope using patient-level cluster bootstrap resampling to obtain Δ = *r* (GCC, HFA) – *r* (OCT-VF, HFA) (and analogously for ρ), along with 95% confidence intervals and *P* values. To assess whether correlations varied with disease severity, we further stratified eyes by mean HFA MD into three groups (MD ≥ −6 dB, −12 dB ≤ MD < −6 dB, and MD < −12 dB) and repeated the correlation comparisons within each stratum using the same bootstrap approach.

Best Available Estimate (BAE) Visual Field Creation and Model Evaluation

Following the creation of matched longitudinal datasets, we generated best available estimate (BAE) visual fields for model evaluation based on the general rationale of Lazaridis et al. (*Am J Ophthalmol*, 2022). Instead of medians, which would require multiple HFA measurements within short time windows and severely restrict sample size, we fit a simple linear regression for each eye (separately

for every test point and for MD) across that eye's longitudinal HFA measurements and evaluated the fitted line at each OCT acquisition date to obtain the theoretical HFA value at the OCT acquisition time. These regression-based values constituted the BAE. This regression-based approach filters short-term measurement noise while preserving sample size and temporal trends. Both BAE and OCT-VF were used at all available OCT time points for each eye (i.e., multiple observations per eye), rather than a single central measurement.

For model evaluation, each OCT-VF estimate was paired with the BAE value computed at the same OCT date, both in the primary longitudinal test dataset and in the GCC analysis dataset, and agreement was assessed using mean absolute error (MAE) and mean error (ME). We used Bland-Altman plots to evaluate the agreement between OCT-VF and BAE parameters. Additionally, we assessed model performance across disease severity by stratifying according to BAE-derived MD and pointwise thresholds and visualizing OCT-VF estimates, absolute errors, and signed errors within each severity stratum. We performed analogous stratified analyses by refractive error and test location.

To characterize model behavior, we performed two additional analyses. First, we compared the frequency distribution of measurements between OCT-VF and BAE for both pointwise thresholds and MD values. Second, to specifically assess behavior at the lower threshold limit, we analyzed subsequent longitudinal measurements for test locations that recorded an initial measurement of 0 dB to evaluate recovery patterns. For each modality (OCT-VF and HFA), we calculated the proportion of measurements that remained at 0 dB versus those that recovered to non-zero values, and computed the mean and standard deviation of non-zero recovery values. This analysis evaluated whether OCT-VF and HFA exhibited similar floor behavior and variability in severely damaged locations over time.

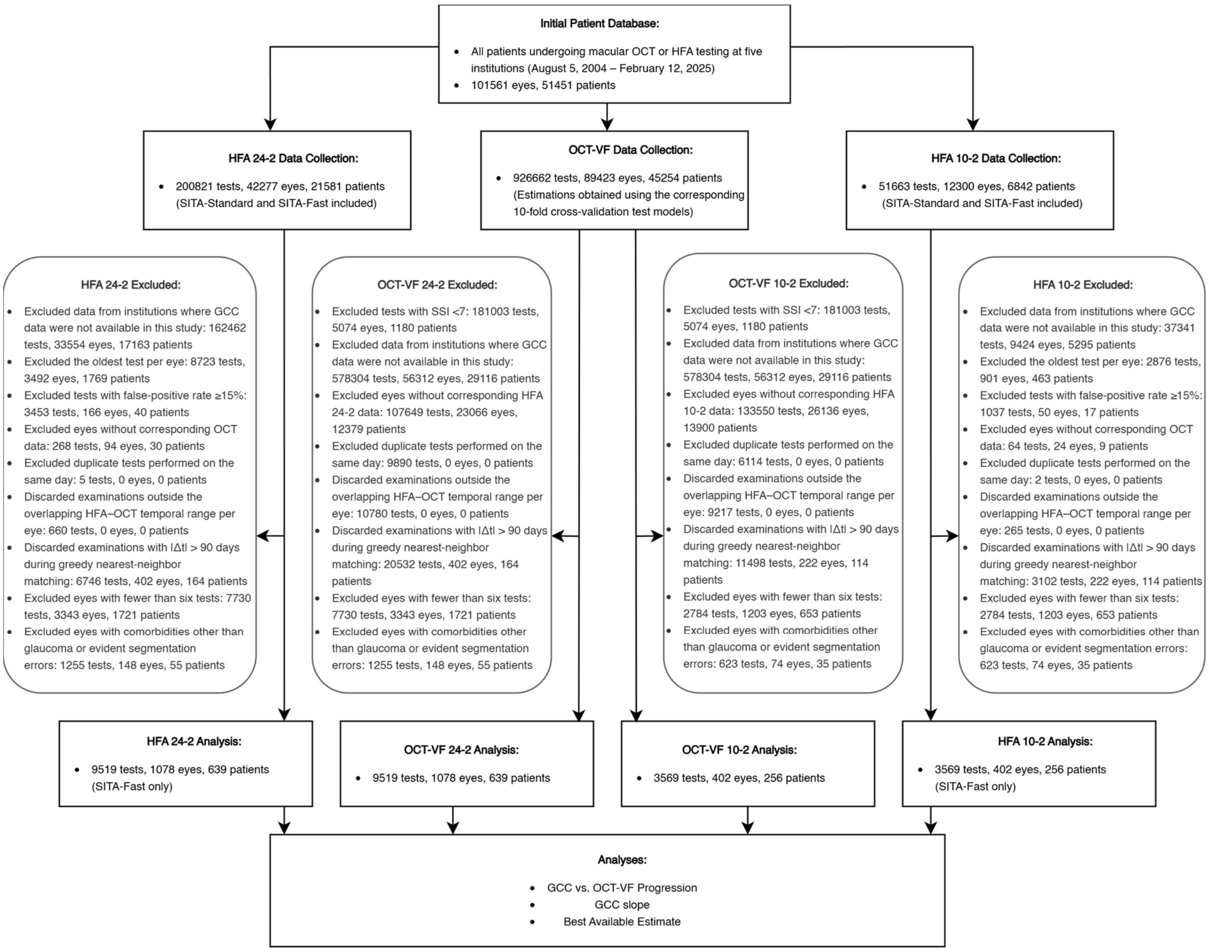

Figure S1. Flowchart for the macular GCC analysis dataset. GCC data were retrospectively curated and integrated during dataset construction. Data from institutions without available GCC measurements were excluded during dataset construction. For each test pattern (24-2 and 10-2), we subsequently excluded eyes with comorbidities other than glaucoma or with evident segmentation errors. The resulting analysis datasets comprised 1078 eyes from 639 patients for the 24-2 pattern and 402 eyes from 256 patients for the 10-2 pattern.

HFA = Humphrey Field Analyzer; OCT = optical coherence tomography; OCT-VF = OCT-based estimated visual field; SITA = Swedish Interactive Threshold Algorithm; GCC = ganglion cell complex.

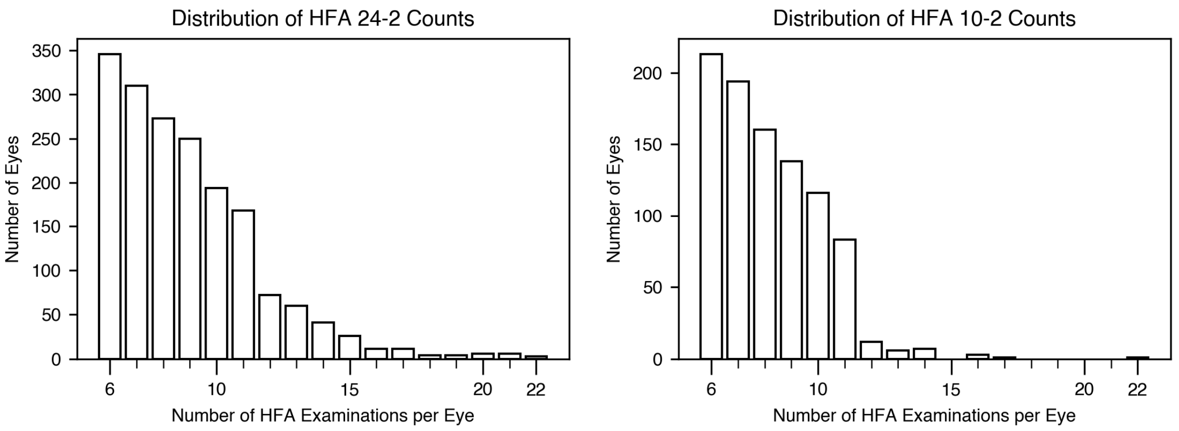

Figure S2. Distribution of HFA examination counts per eye in the longitudinal test dataset. The 24-2 dataset included 1785 eyes from 1000 patients, and the 10-2 dataset included 934 eyes from 533 patients. The number of examinations per eye was identical for OCT-VF after temporal matching between modalities.

HFA = Humphrey Field Analyzer; OCT = optical coherence tomography; OCT-VF = OCT-based estimated visual field.

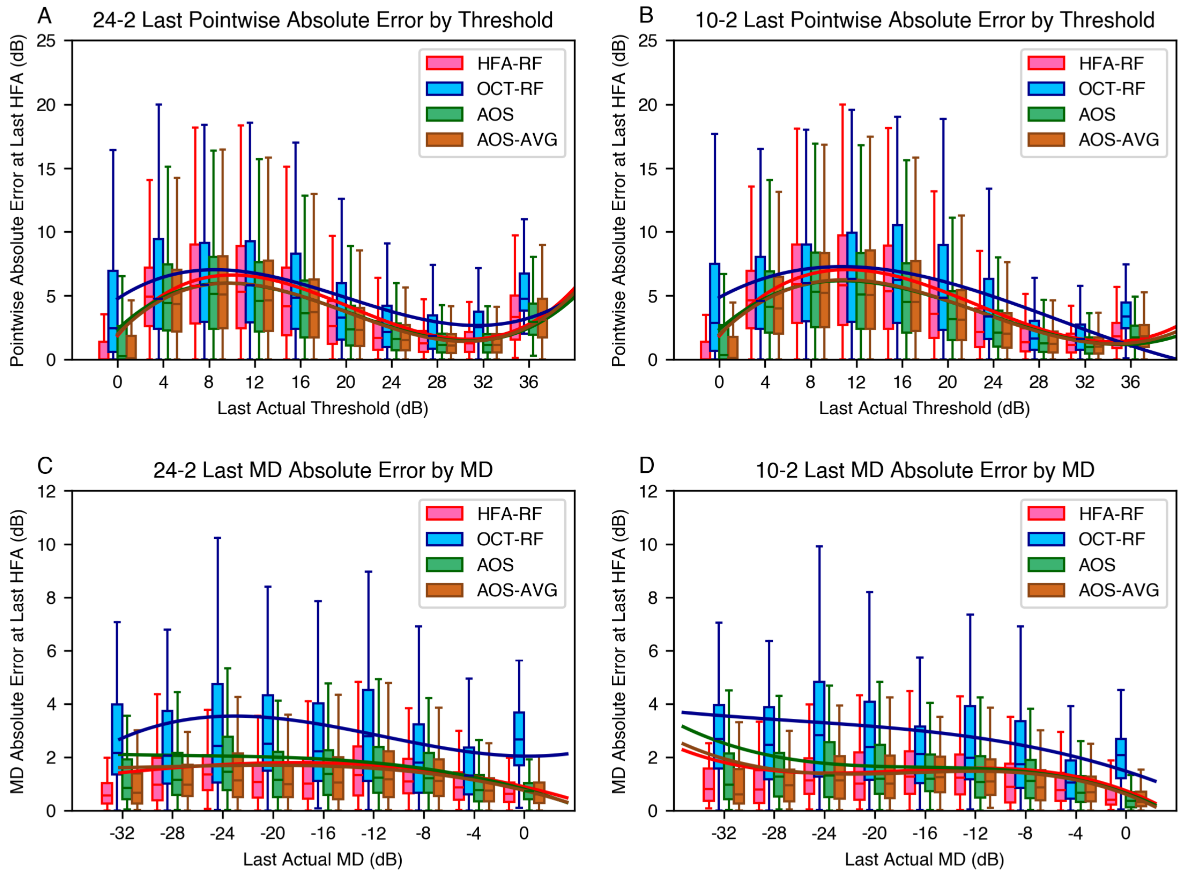

Figure S3. Absolute error of last HFA forecasts by disease severity. (A, B) Pointwise thresholds and (C, D) MD for 24-2 (A, C) and 10-2 (B, D). Boxplots show the distribution of absolute error (|forecast − last observed HFA|; dB) at each severity on the x-axis (A, B: last actual threshold; C, D: last actual MD). Whiskers indicate the non-outlier range, together with a cubic regression curve. Methods: HFA-RF (red), linear trend fitted using all HFA measurements obtained prior to the last HFA and extrapolated to the last HFA timepoint; OCT-RF (blue), linear trend fitted using all OCT-VF measurements prior to the last HFA and extrapolated to the last HFA timepoint; AOS (green), HFA-anchored intercept defined by the mean of prior HFA measurements combined with the OCT-VF–derived slope estimated prior to the last HFA; AOS-AVG (brown), HFA-anchored intercept defined by the mean of prior HFA measurements combined with the average of OCT-VF–derived and HFA–derived slopes estimated prior to the last HFA. Across panels, OCT-RF generally exhibited larger absolute errors. Lower values indicate better forecasts.

HFA = Humphrey Field Analyzer; MD = mean deviation; OCT = optical coherence tomography; OCT-VF = OCT-based estimated visual field; OCT-RF = OCT-VF regression forecast; AOS = HFA-anchored OCT-VF slope forecast; AOS-AVG = HFA-anchored OCT-VF/HFA averaged-slope forecast; HFA-RF = HFA regression forecast.

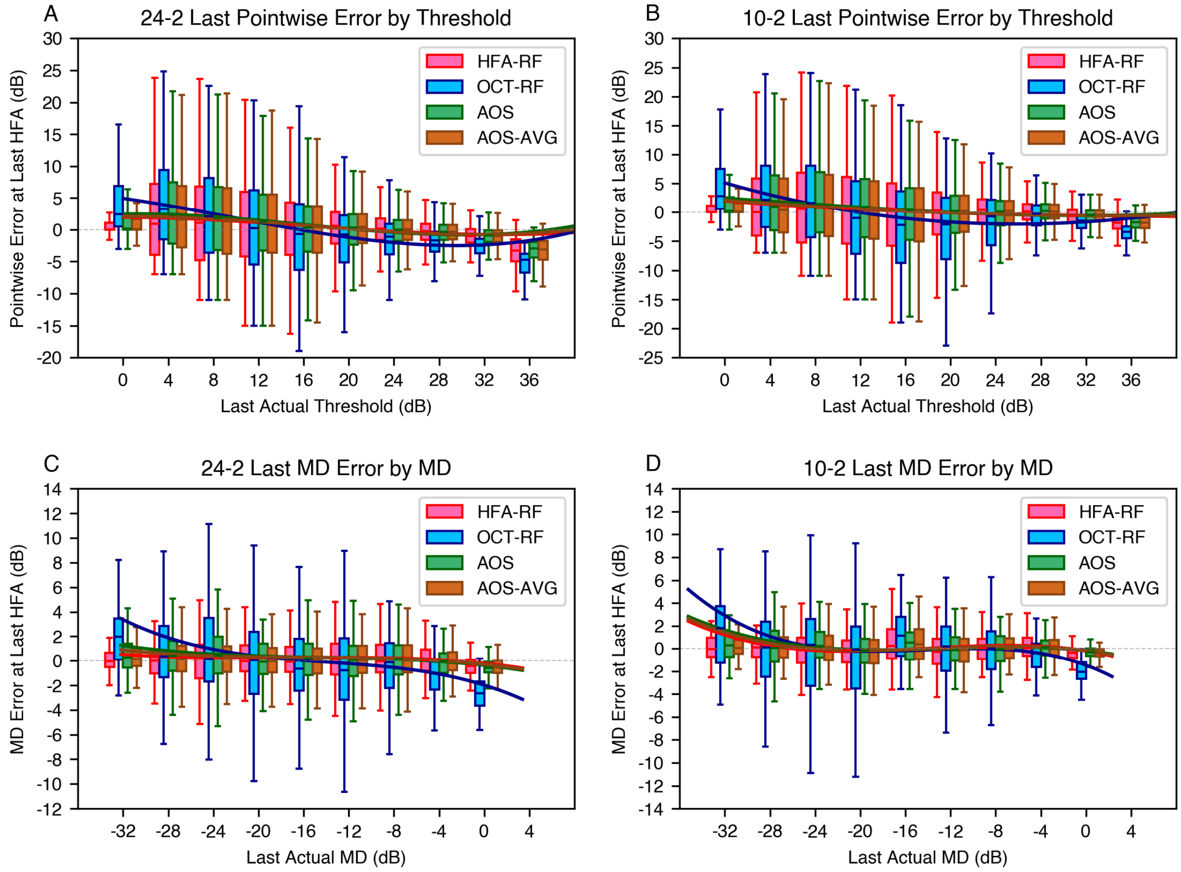

Figure S4. Error of last HFA forecasts by disease severity. (A, B) Pointwise thresholds and (C, D) MD for 24-2 (A, C) and 10-2 (B, D). Boxplots show the distribution of error (forecast – observed last HFA; dB) across each severity on the x-axis (A, B: actual last threshold; C, D: actual last MD). Whiskers indicate the non-outlier range, together with a cubic regression curve. Methods: HFA-RF (red), linear trend using all prior HFA measurements extrapolated to the last HFA; OCT-RF (blue), linear trend using all OCT-VF measurements obtained prior to the last HFA extrapolated to the last HFA; AOS (green), HFA-anchored intercept defined by the mean of all prior HFA measurements combined with the OCT-VF-derived slope estimated from measurements preceding the last HFA; AOS-AVG (brown), HFA-anchored intercept defined by the mean of prior HFA measurements combined with the average of OCT-VF–derived and HFA–derived slopes estimated prior to the last HFA. Across all panels, OCT-RF showed relatively larger errors at the extremes of disease severity, consistent with shrinkage toward the center. Values closer to zero indicate better forecasts.

HFA = Humphrey Field Analyzer; MD = mean deviation; OCT = optical coherence tomography; OCT-VF = OCT-based estimated visual field; OCT-RF = OCT-VF regression forecast; AOS = HFA-anchored OCT-VF slope forecast; AOS-AVG = HFA-anchored OCT-VF/HFA averaged-slope forecast; HFA-RF = HFA regression forecast.

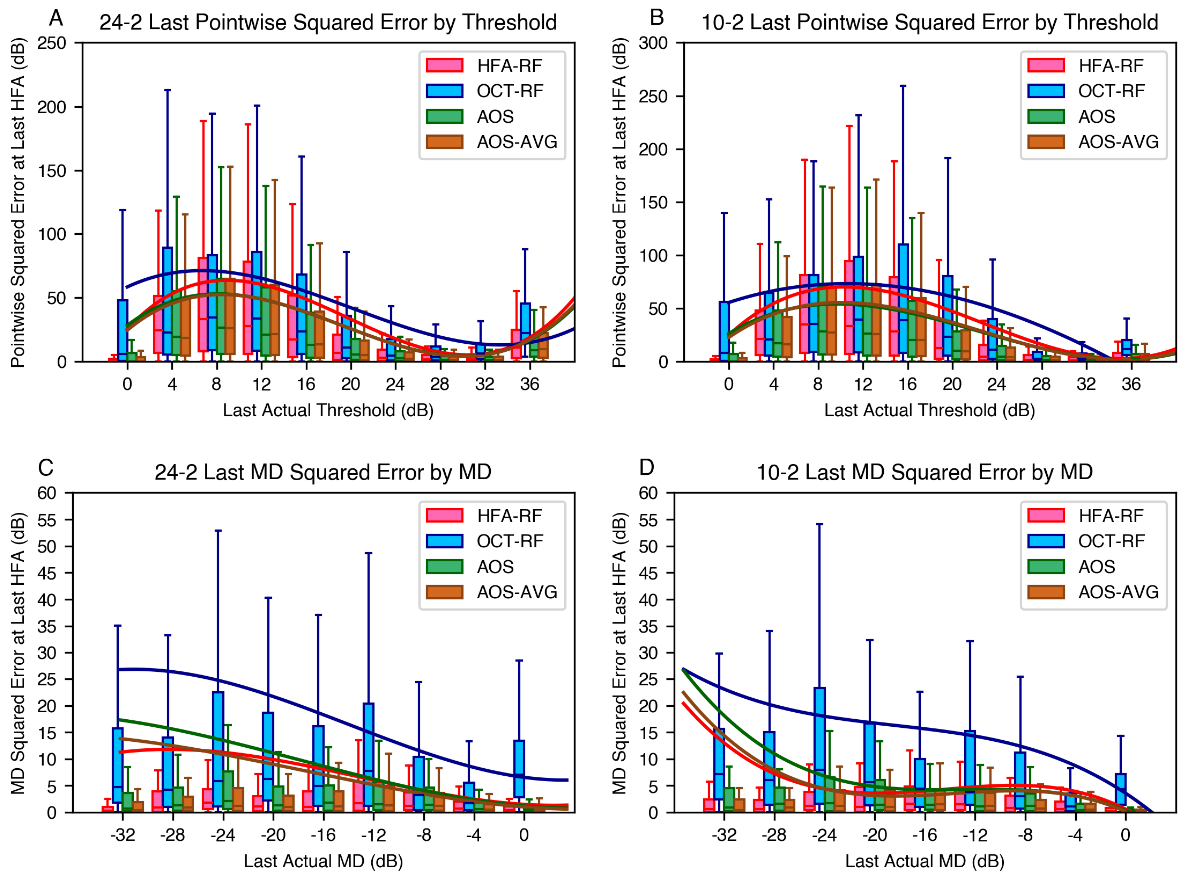

Figure S5. Squared error of last HFA forecasts by disease severity. (A, B) Pointwise thresholds and (C, D) MD for 24-2 (A, C) and 10-2 (B, D). Boxplots show the distribution of squared error (forecast – observed last HFA; dB^2) across each severity on the x-axis (A, B: actual last threshold; C, D: actual last MD). Whiskers indicate the non-outlier range, together with a cubic regression curve. Methods: HFA-RF (red), linear trend using all prior HFA measurements extrapolated to the last HFA; OCT-RF (blue), linear trend using all OCT-VF measurements obtained prior to the last HFA extrapolated to the last HFA; AOS (green), HFA-anchored intercept defined by the mean of all prior HFA measurements combined with the OCT-VF-derived slope estimated from measurements preceding the last HFA; AOS-AVG (brown), HFA-anchored intercept defined by the mean of prior HFA measurements combined with the average of OCT-VF–derived and HFA–derived slopes estimated prior to the last HFA. OCT-RF shows larger errors overall. Lower values indicate better forecasts.

HFA = Humphrey Field Analyzer; MD = mean deviation; OCT = optical coherence tomography; OCT-VF = OCT-based estimated visual field; OCT-RF = OCT-VF regression forecast; AOS = HFA-anchored OCT-VF slope forecast; AOS-AVG = HFA-anchored OCT-VF/HFA averaged-slope forecast; HFA-RF = HFA regression forecast.

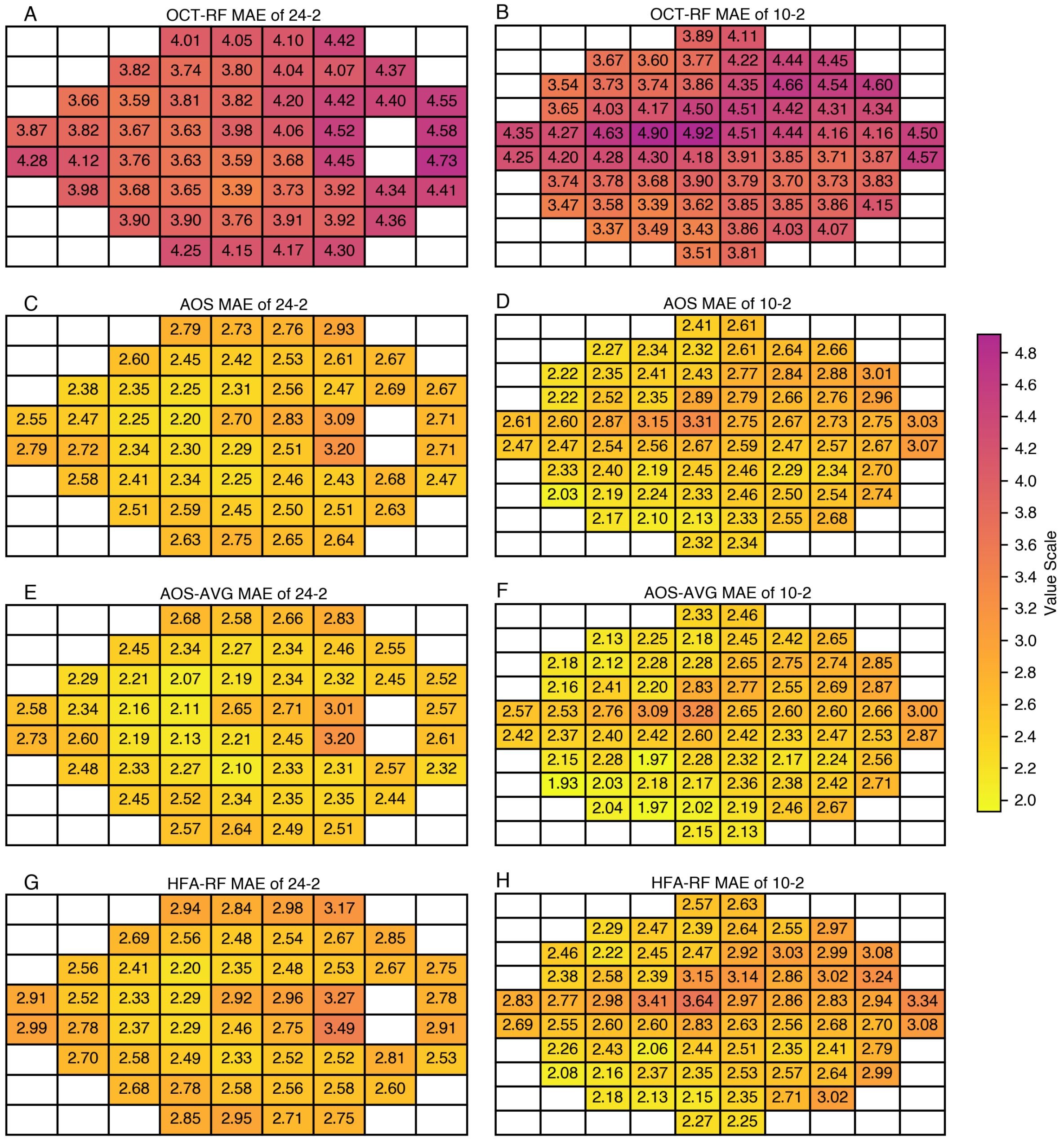

Figure S6. Pointwise MAE heatmaps for forecasting the last HFA. Panels show the mean absolute error (MAE; |forecast – last observed HFA|, dB) at each test location for three forecasting methods: OCT-RF (A, B), AOS (C, D), AOS-AVG (E, F), and HFA-RF (G, H). OCT-RF: linear regression of all OCT-VF tests prior to the last HFA, extrapolated to the last visit. AOS: HFA-anchored intercept using prior HFA measurements combined with the OCT-VF slope estimated from OCT-VF tests temporally preceding the last HFA. AOS-AVG: HFA-anchored intercept defined by the mean of prior HFA measurements combined with the average of OCT-VF–derived and HFA–derived slopes estimated prior to the last HFA. HFA-RF: linear regression of prior HFA examinations extrapolated to the last visit. The left column depicts 24-2 (A, C, E, G) and the right column 10-2 (B, D, F, H). Warmer colors indicate larger MAE. Across test locations, AOS-AVG consistently showed the lowest MAE, with values equal to or lower than AOS and uniformly lower than HFA-RF, whereas OCT-RF generally exhibited larger errors.

MAE = mean absolute error; OCT-VF = OCT-based estimated visual field; OCT-RF = OCT-VF regression forecast; AOS = HFA-anchored OCT-VF slope forecast; AOS-AVG = HFA-anchored OCT-VF/HFA averaged-slope forecast; HFA-RF = HFA regression forecast.

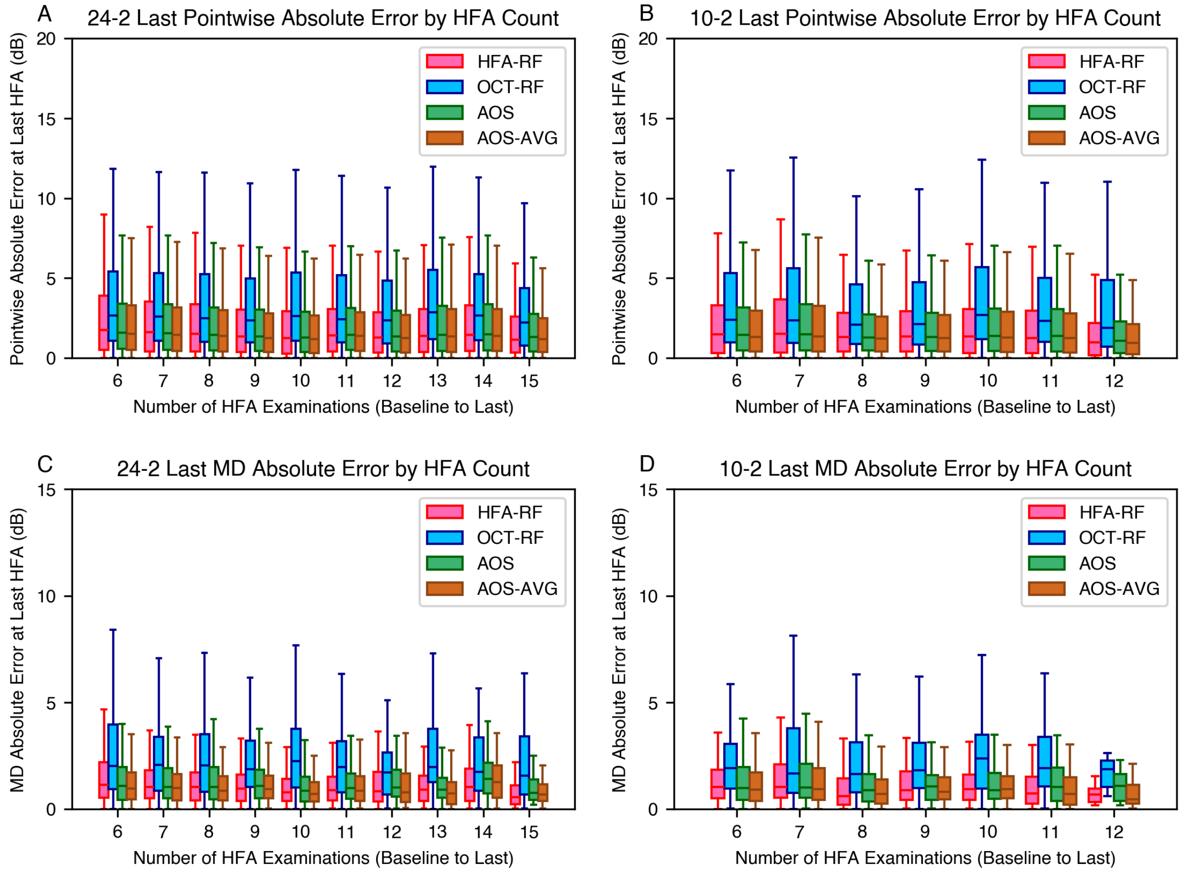

Figure S7. Prediction absolute error at the last HFA examination stratified by the number of prior HFA tests. Methods: HFA-RF (red), linear trend using all prior HFA measurements extrapolated to the last HFA; OCT-RF (blue), linear trend using all OCT-VF measurements obtained prior to the last HFA extrapolated to the last HFA; AOS (green), HFA-anchored intercept defined by the mean of all prior HFA measurements combined with the OCT-VF-derived slope estimated from measurements preceding the last HFA; AOS-AVG (brown), HFA-anchored intercept defined by the mean of prior HFA measurements combined with the average of OCT-VF–derived and HFA–derived slopes estimated prior to the last HFA. The x-axis range was restricted to examination counts with sufficient sample sizes (24-2 $\leq$ 15, 10-2 $\leq$ 12) to avoid unstable variance estimates at extreme counts. Variability at higher examination counts is strongly influenced by reduced sample sizes (see Fig. S2).
HFA = Humphrey Field Analyzer; MD = mean deviation; OCT = optical coherence tomography; OCT-VF = OCT-based estimated visual field; OCT-RF = OCT-VF regression forecast; AOS = HFA-anchored OCT-VF slope forecast; AOS-AVG = HFA-anchored OCT-VF/HFA averaged-slope forecast; HFA-RF = HFA regression forecast.

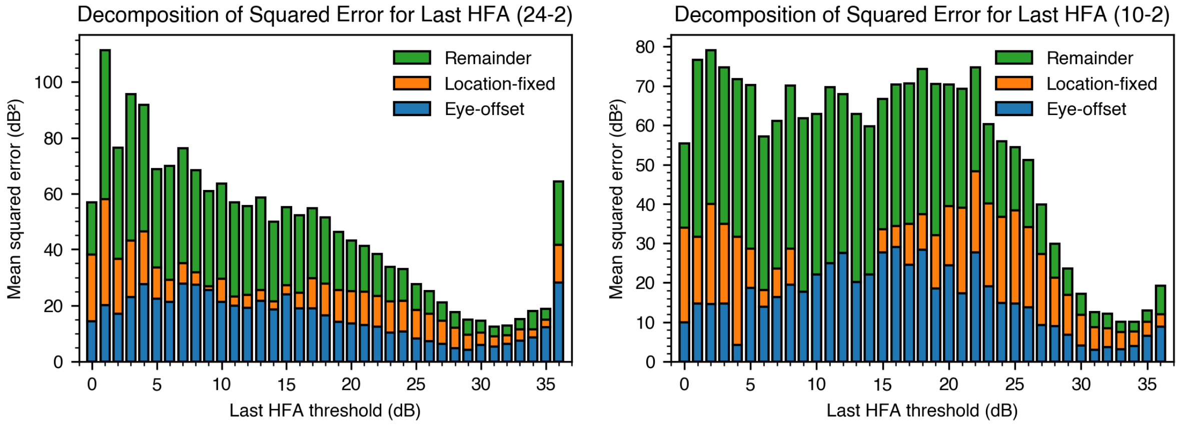

Figure S8. Decomposition of squared error for the last HFA by threshold. For each 1-dB bin of the last observed HFA threshold, the stacked bars display the MSE (dB^2) of OCT-RF forecasts, partitioned into Eye-offset, Location-fixed, and Remainder components. Eye-offset represents error attributable to eye-level intercept bias and is computed as Total MSE – MD-offset MSE; Location-fixed represents location-specific systematic bias and is computed as MD-offset MSE − Remainder MSE; Remainder is the remainder obtained after AOS anchoring. Here, MD-offset MSE is the squared error formed after centering each modality by its own MD at the last HFA timepoint—i.e., comparing (threshold – MD) for OCT-RF and for HFA so that eye-level offsets are removed before error is computed. Errors increase with disease severity (lower thresholds), primarily due to the Remainder component, with additional contributions from Eye-offset and Location-fixed. Lower bars indicate smaller errors.

HFA = Humphrey Field Analyzer; OCT = optical coherence tomography; OCT-VF = OCT-based estimated visual field; OCT-RF = OCT-VF regression forecast; MD = mean deviation; AOS = HFA-anchored OCT-VF slope forecast; HFA-RF = HFA regression forecast; MSE = mean squared error.

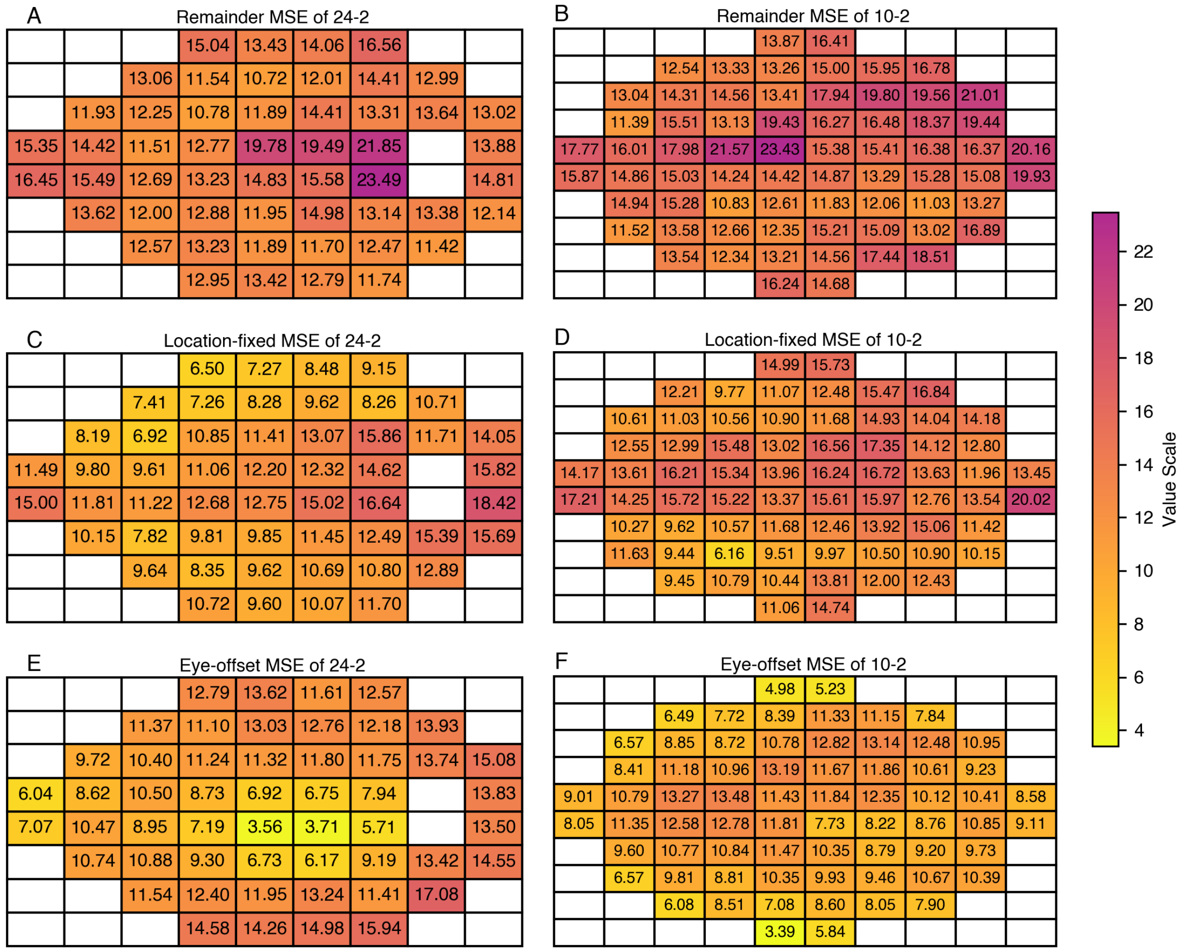

Figure S9. Spatial decomposition of squared error for the last HFA forecast. Heatmaps display the distribution of (A, B) Remainder MSE, (C, D) Location-fixed MSE, and (E, F) Eye-offset MSE for 24-2 and 10-2 test patterns. (A, B) Remainder MSE: This component represents residual error after accounting for eye- and location-specific biases. High errors correspond to the regions most susceptible to glaucomatous damage (e.g., the nasal step in 24-2 and the superior arcuate region in 10-2), where threshold variability (standard deviation) is inherently high. (C, D) Location-fixed MSE: This represents systematic bias at specific test points. In 24-2, errors are notably elevated in the temporal field (corresponding to the optic disc). This is attributable to limited structural information, as the optic disc lies at the periphery or outside the 9 × 9 mm macular OCT scan area. (E, F) Eye-offset MSE: This reflects the error in predicting the eye-level Mean Deviation (MD). In 24-2, errors are higher in the periphery, likely because peripheral defects (e.g., nasal steps) occur outside the OCT scan area, making it difficult to estimate the global damage level. In 10-2, errors concentrate in the superior central region; this aligns with the area of highest population variance.

MSE = mean squared error; HFA = Humphrey Field Analyzer; OCT = optical coherence tomography; MD = mean deviation.

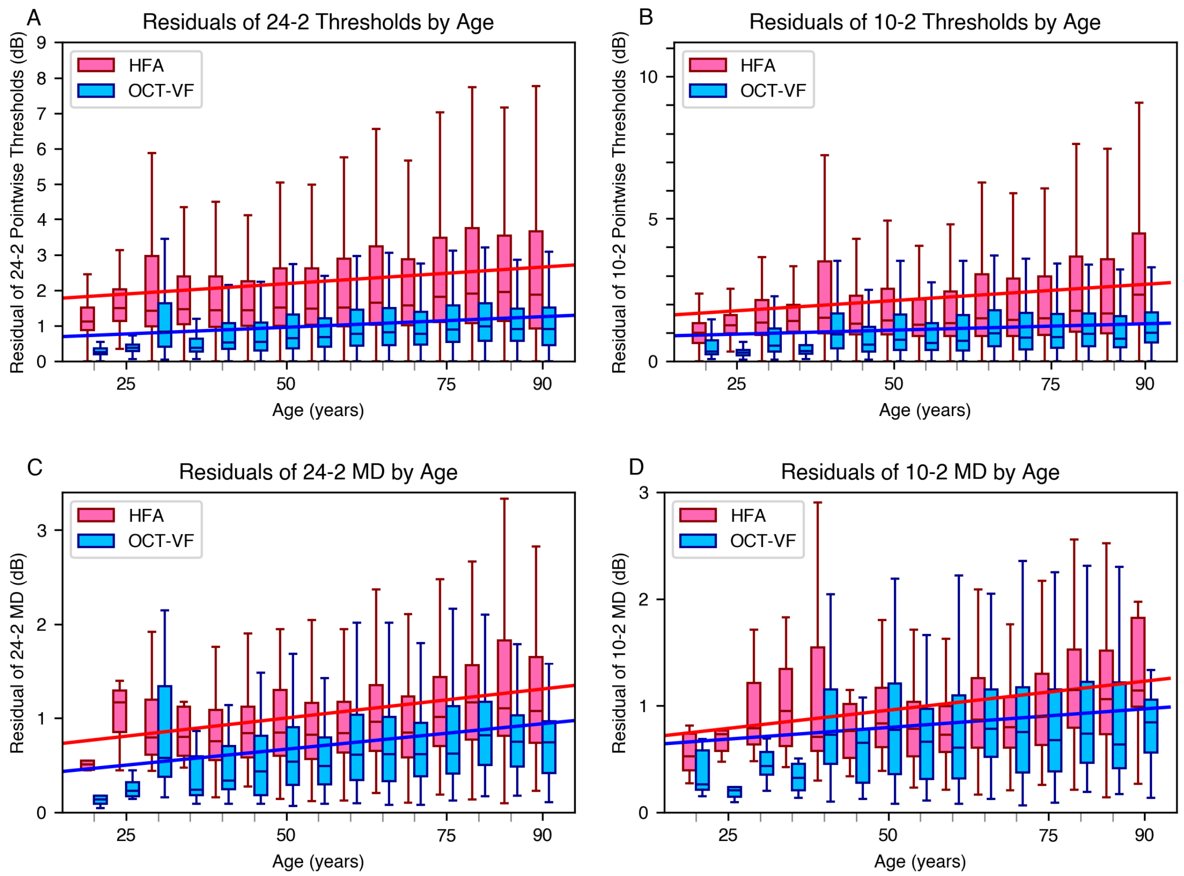

Figure S10. Comparison of residual variability between HFA measurements and OCT-VF across age groups. (A) 24-2 pointwise thresholds, (B) 10-2 pointwise thresholds, (C) 24-2 MD, and (D) 10-2 MD. The horizontal axis represents age in 5-year increments, and the vertical axis represents residual variability. Residual variability was calculated as the mean absolute residuals from jackknife regression lines fitted to each eye's longitudinal data for both OCT-VF and HFA. Each panel shows boxplots of residuals for each age group, along with linear regression lines for HFA (red) and OCT-VF (blue). Across age groups, OCT-VF generally exhibited lower residual variability than HFA. Both methods show increased variability with age. Across panels, HFA exhibited larger regression slopes than OCT-VF, suggesting a greater age-related increase in variability. The slopes of the regression lines (in dB/year) for HFA and OCT-VF are (A) 0.0117 vs. 0.0075, (B) 0.0143 vs. 0.0056, (C) 0.0077 vs. 0.0067, and (D) 0.0068 vs. 0.0043, respectively.

HFA = Humphrey Field Analyzer; OCT = optical coherence tomography; OCT-VF = OCT-based estimated visual field; MD = mean deviation.

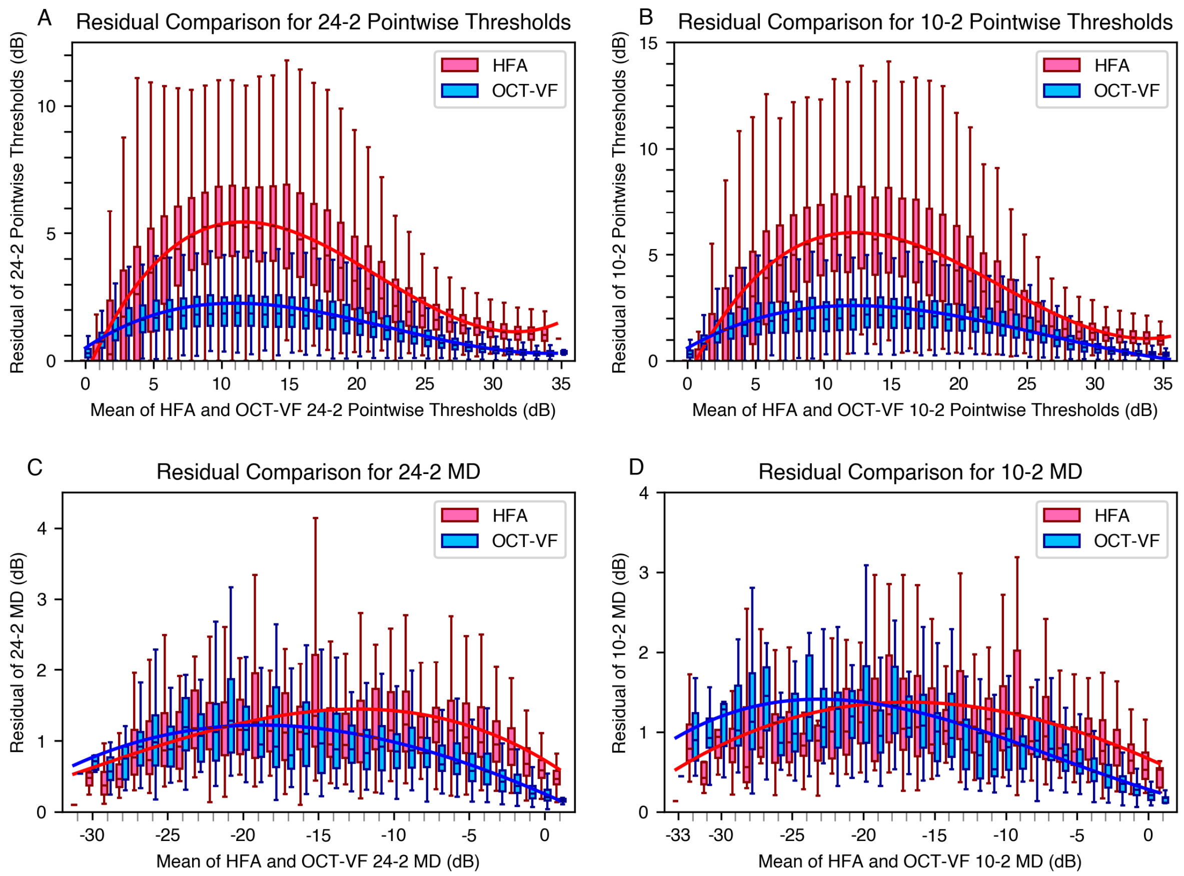

Figure S11. Comparison of residual variability between OCT-VF and HFA measurements across VF severity levels. (A) 24-2 thresholds (pointwise values), (B) 10-2 thresholds (pointwise values), (C) 24-2 MD, and (D) 10-2 MD. The horizontal axis represents VF severity, and the vertical axis represents residual variability. Residual variability was calculated as the mean absolute residuals from jackknife regression lines fitted to each eye's longitudinal data for both OCT-VF and HFA. Smaller residual variability indicates less fluctuation in repeated measurements. Each panel shows boxplots of residuals for each severity and cubic regression lines. For pointwise thresholds (A, B), OCT-VF generally exhibits lower variability than HFA across the dynamic range, with convergence observed near the 0 dB floor. For MD (C, D), OCT-VF shows lower variability than HFA in mild-to-severe cases, though the difference narrows and reverses in advanced disease. For each time point, the x-axis values represent the average of each measurement and its closest time-matched counterpart from the other modality.

HFA = Humphrey Field Analyzer; OCT = optical coherence tomography; VF = visual field; OCT-VF = OCT-based estimated visual field; MD = mean deviation.

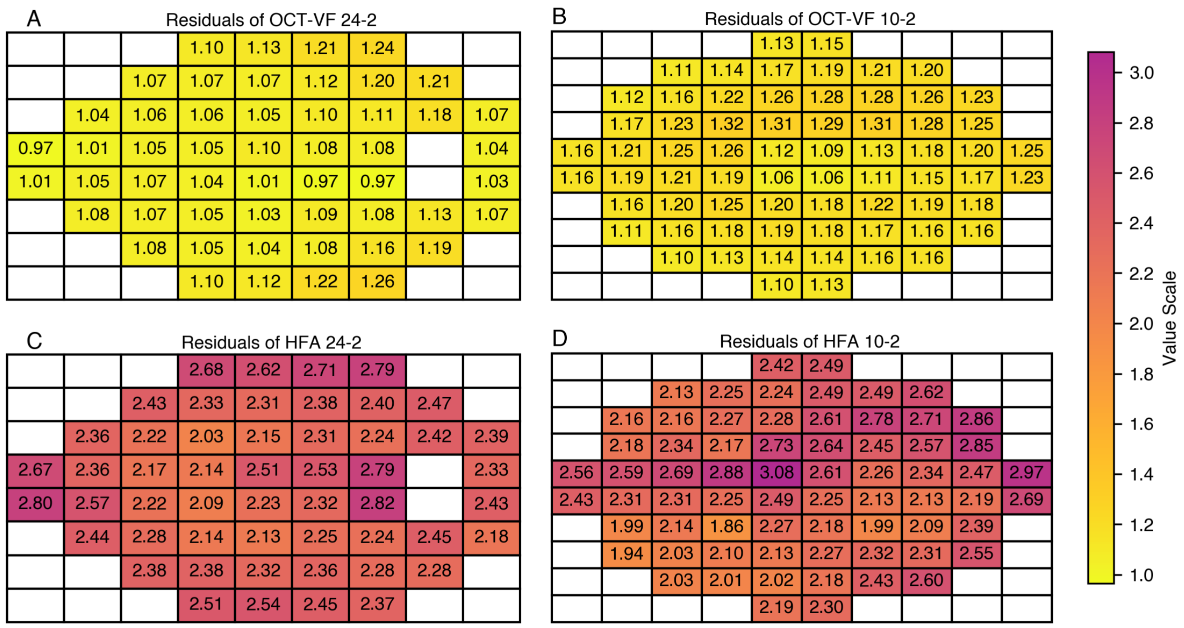

Figure S12. Heat maps of residual variability for each test point. (A) OCT-VF 24-2, (B) OCT-VF 10-2, (C) HFA 24-2, and (D) HFA 10-2. Residual variability was calculated as the mean absolute residuals from jackknife regression lines fitted to each eye's longitudinal data for both OCT-VF and HFA. Smaller residual variability indicates less fluctuation in repeated measurements. The color scale represents the magnitude of residual variability at each test point. OCT-VF shows consistently lower residual variability than HFA measurements across all test points, with values ranging from 0.97 to 1.32 dB for OCT-VF compared to 1.86 to 3.08 dB for HFA measurements. We horizontally flipped the left eye data and integrated them with the right eye data.

OCT = optical coherence tomography; OCT-VF = OCT-based estimated visual field; HFA = Humphrey Field Analyzer.

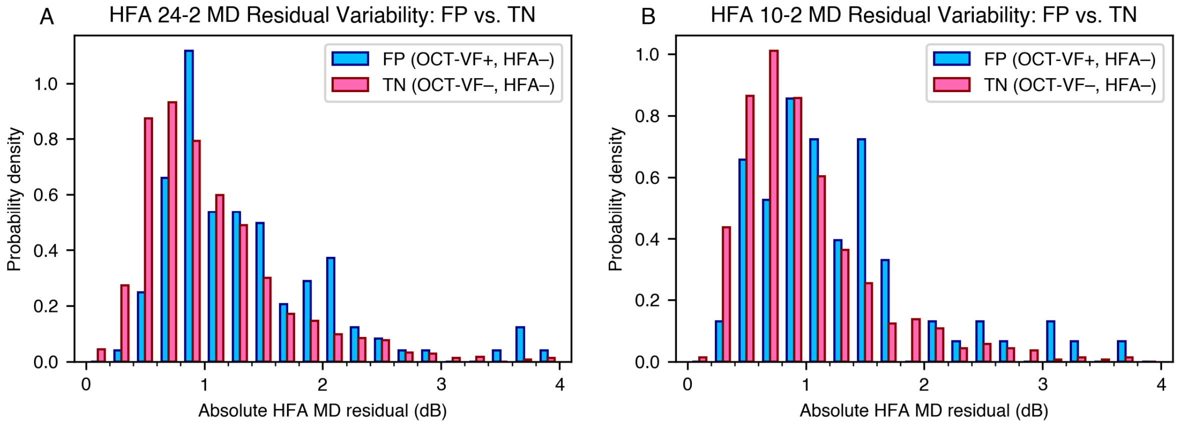

Figure S13. Distribution of absolute HFA MD residuals (residual variability) for false-positive (FP) and true-negative (TN) eyes. Residuals were calculated as deviations from linear trends using jackknife resampling, where absolute deviations from the regression line were averaged across jackknife iterations. Lower residual values indicate reduced measurement variability and better longitudinal consistency. False-positive (FP) and true-negative (TN) eyes were defined using HFA as the reference standard. Progression was identified when MD slope ≤ –0.5 dB/year and $P < 0.025$, and the same criteria were applied to OCT-VF. FP was defined as OCT-VF+, HFA–, and TN as OCT-VF–, HFA–; detailed confusion matrices are provided in Supplementary Table S6. Panels A (24-2) and B (10-2) show probability density histograms comparing HFA MD residuals between FP eyes (n = 125 for 24-2, n = 77 for 10-2; OCT-VF+, HFA–) and TN eyes (n = 1380 for 24-2, n = 698 for 10-2; OCT-VF–, HFA–). FP eyes exhibited significantly larger residual variability than TN eyes for both the 24-2 and 10-2 patterns ($P < 0.001$ for both; see Table S7).

HFA = Humphrey Field Analyzer; MD = mean deviation; FP = false-positive; TN = true-negative; OCT = optical coherence tomography; OCT-VF = OCT-based estimated visual field.

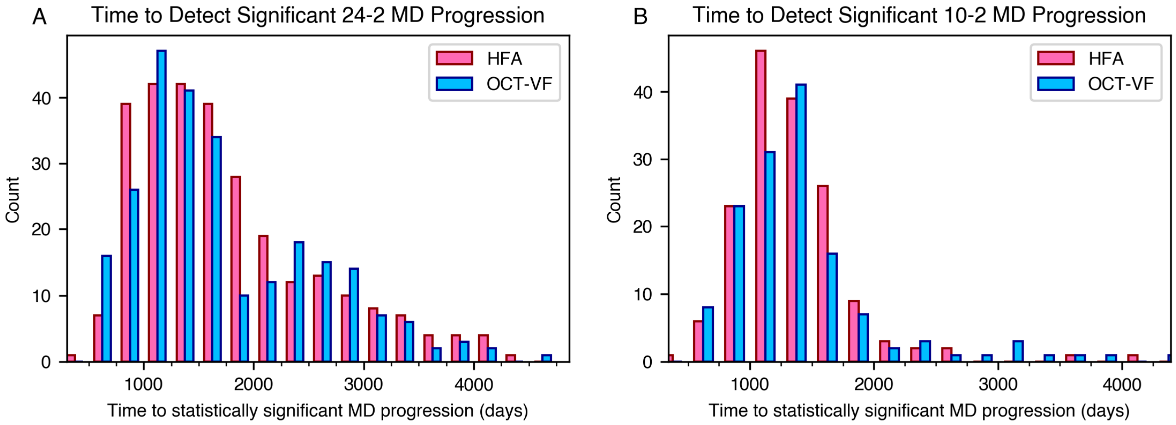

Figure S14. Time required to detect MD progression. (A) 24-2 and (B) 10-2 patterns. Bars show the number of eyes by the number of days required to reach the prespecified statistical criterion for significant negative MD slope (slope ≤ –0.5 dB/year with two-sided P < 0.025) for HFA (red) and OCT-VF (blue). Analyses were restricted to eyes showing significant progression by both modalities (24-2: *n* = 129, 10-2: *n* = 63). Although the distributions differ visually, no statistically significant differences were observed between modalities for either test pattern (see Table S9).
MD = mean deviation; HFA = Humphrey Field Analyzer; OCT = optical coherence tomography; OCT-VF = OCT-based estimated visual field.

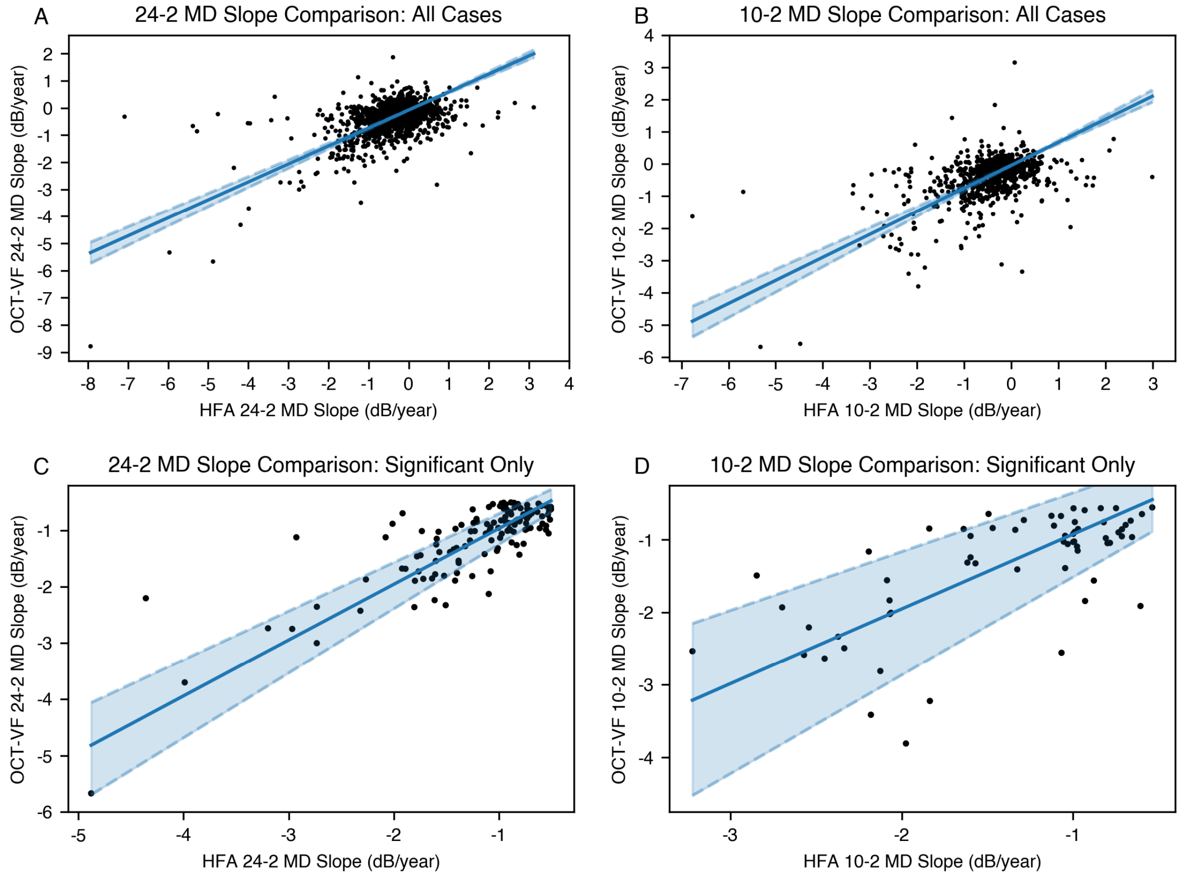

Figure S15. Scatterplots comparing MD slopes between HFA and OCT-VF. (A) 24-2 MD slopes for all eyes (n = 1785). Passing–Bablok regression slope: 0.663 (95% CI: 0.616 to 0.711), Pearson's r = 0.537, $P < 0.001$. (B) 10-2 MD slopes for all eyes (n = 934). Passing–Bablok regression slope: 0.716 (95% CI: 0.650 to 0.786), Pearson's r = 0.505, $P < 0.001$. (C) 24-2 MD slopes for eyes with significant progression in both OCT-VF and HFA (slope ≤ –0.5 dB/year and $P < 0.025$, n = 129). Passing–Bablok regression slope: 0.993 (95% CI: 0.866 to 1.146), Pearson's r = 0.822, $P < 0.001$. (D) 10-2 MD slopes for eyes with significant progression in both methods (n = 63). Passing–Bablok regression slope: 1.031 (95% CI: 0.812 to 1.353), Pearson's r = 0.665, $P < 0.001$. Each panel shows the MD slope for each eye as measured by HFA (x-axis) versus OCT-VF (y-axis). The solid line represents the Passing–Bablok regression line, and the light blue shaded area indicates its 95% confidence interval.
HFA = Humphrey Field Analyzer; OCT = optical coherence tomography; OCT-VF = OCT-based estimated visual field; MD = mean deviation; CI = confidence interval.

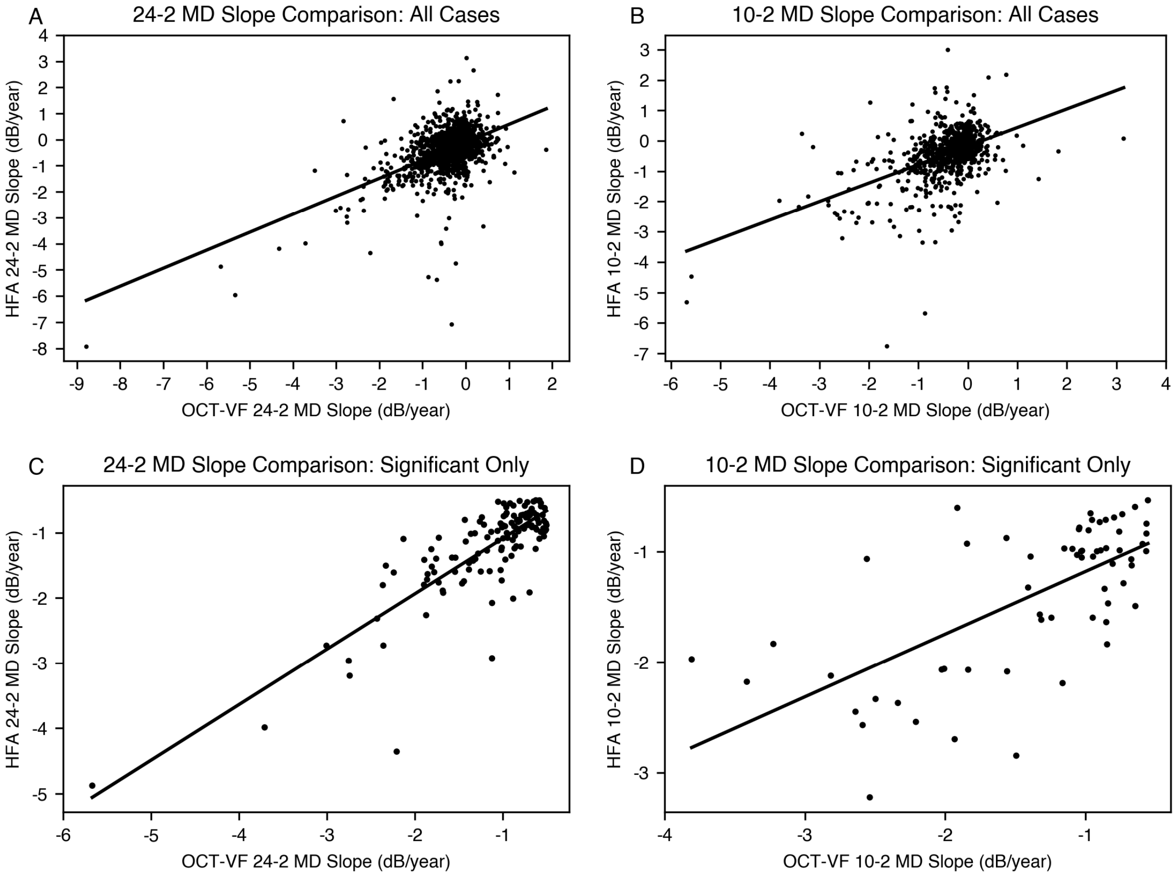

Figure S16. Scatterplots comparing MD slopes between HFA and OCT-VF with axes reversed. (A) 24-2 MD slopes for all eyes ($n = 1785$). Regression slope: 0.690. (B) 10-2 MD slopes for all eyes ($n = 934$). Regression slope: 0.610. (C) 24-2 MD slopes for eyes with significant progression in both OCT-VF and HFA (slope ≤ -0.5 dB/year and $P < 0.025$, $n = 129$). Regression slope: 0.851. (D) 10-2 MD slopes for eyes with significant progression in both methods ($n = 63$). Regression slope: 0.567. Each panel shows the MD slope for each eye as measured by OCT-VF (x-axis) versus HFA (y-axis). The solid line represents the ordinary least-squares regression line. These plots use the same observations as Figure S15 but with the axes reversed.

HFA = Humphrey Field Analyzer; OCT = optical coherence tomography; OCT-VF = OCT-based estimated visual field; MD = mean deviation.

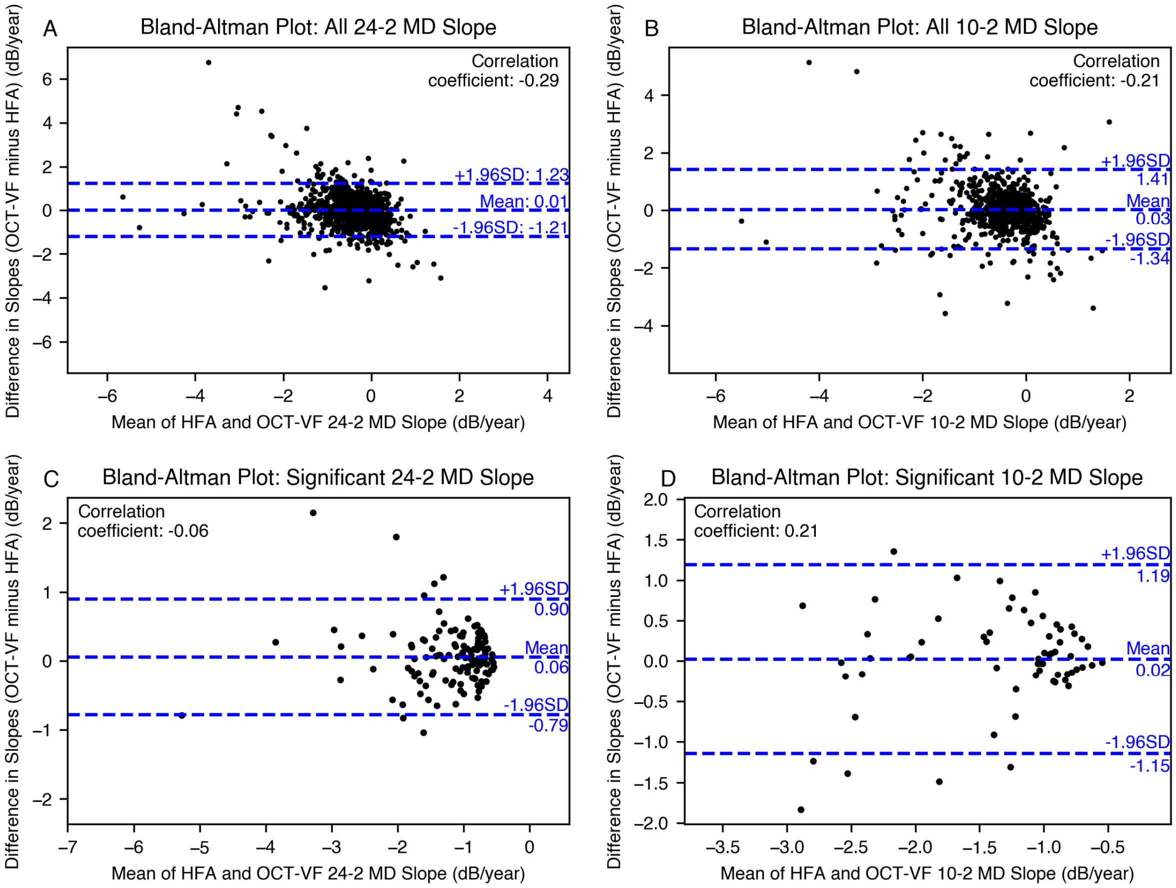

Figure S17. Bland-Altman plots comparing progression rates (MD slopes) between OCT-VF and HFA. (A) 24-2 MD slopes for all eyes (n = 1785), (B) 10-2 MD slopes for all eyes (n = 934), (C) 24-2 MD slopes for eyes with significant progression in both OCT-VF and HFA (slope ≤ –0.5 dB/year and P < 0.025, n = 129), and (D) 10-2 MD slopes for eyes with significant progression in both methods (n = 63). Each panel plots the difference in slopes (OCT-VF minus HFA) against the mean slope of the two methods. Mean differences ranged from 0.01 to 0.06 dB/year, with 95% limits of agreement shown as dashed lines. Correlation coefficients ranged from –0.29 to 0.21.

MD = mean deviation; HFA = Humphrey Field Analyzer; OCT = optical coherence tomography; OCT-VF = OCT-based estimated visual field.

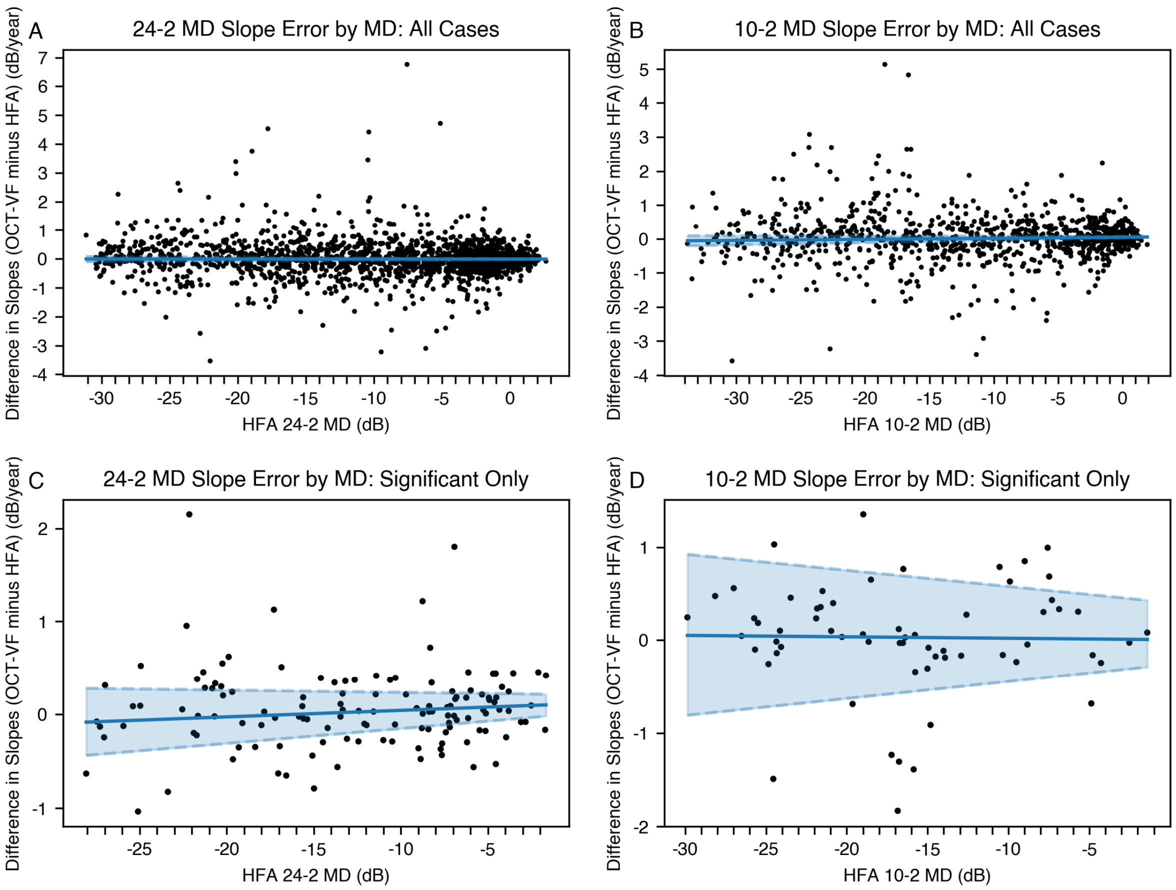

Figure S18. MD slope error versus MD. (A) 24-2 (all eyes, n = 1785), (B) 10-2 (all eyes, n = 934), (C) 24-2 (eyes significant in both modalities, n = 129), and (D) 10-2 (significant in both, n = 63). The y-axis shows the difference in MD slopes (OCT-VF − HFA; dB/year), and the x-axis shows the eye-level mean HFA MD (dB). The solid line represents the Passing–Bablok regression line, and the light blue shaded area indicates its 95% confidence interval. Estimated slopes (units = 1/year, i.e., change in slope error per 1-dB higher MD) were near zero in all panels: A −0.0001 [−0.0024, 0.0023], B 0.0031 [−0.0003, 0.0065], C 0.0070 [−0.0024, 0.0159], and D −0.0015 [−0.0174, 0.0183]. Because all CIs include zero, the OCT-VF–HFA slope difference shows no systematic dependence on MD over the observed range.

MD = mean deviation; OCT = optical coherence tomography; OCT-VF = OCT-based estimated visual field; HFA = Humphrey Field Analyzer; CI = confidence interval.

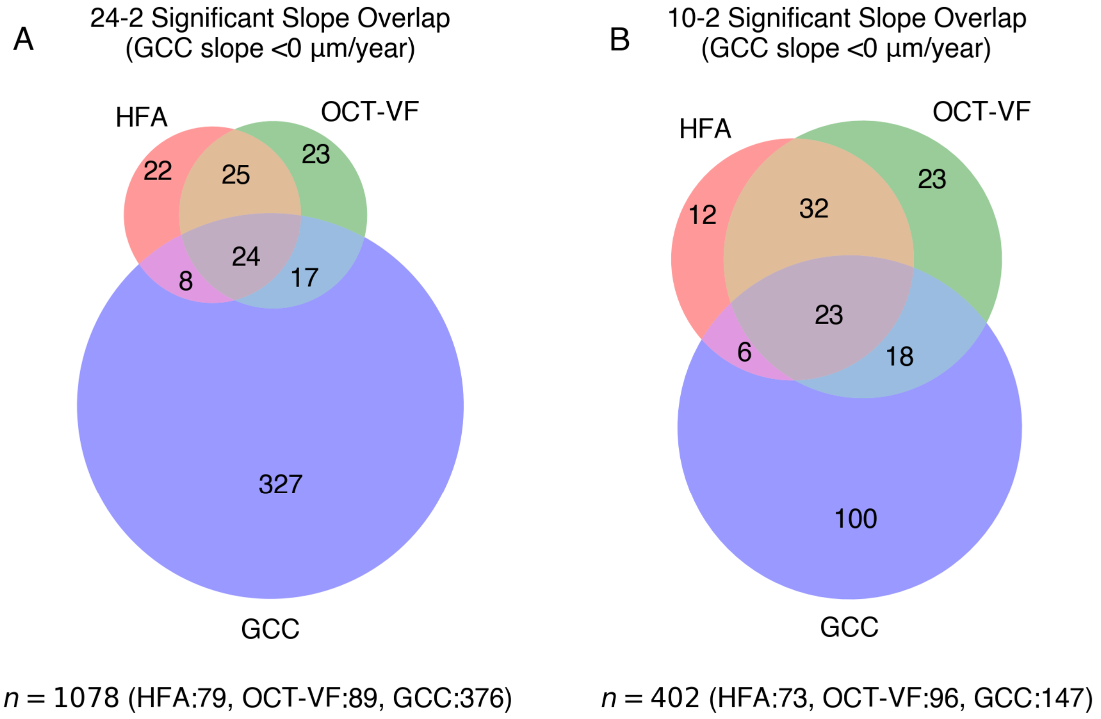

Figure S19. Overlap of eyes with significant progression by method (GCC dataset, GCC slope <0 µm/year). (A) 24-2 ($n = 1078$) and (B) 10-2 ($n = 402$). Venn diagrams show the counts of eyes classified as progressing by HFA (MD slope), OCT-VF (MD slope), and GCC (macular GCC thickness slope). Progression was defined, for HFA and OCT-VF, as MD slope ≤–0.5 dB/year with two-sided $P < 0.025$ (Bonferroni across 24-2 vs 10-2), and, for GCC, as thickness slope <0 µm/year with two-sided $P < 0.025$ (same correction). Numbers within each region indicate eyes meeting each criterion and their overlaps. This dataset corresponds to the single-center GCC cohort (Table S11 / Fig. S1).
HFA = Humphrey Field Analyzer; OCT = optical coherence tomography; OCT-VF = OCT-based estimated visual field; GCC = ganglion cell complex.

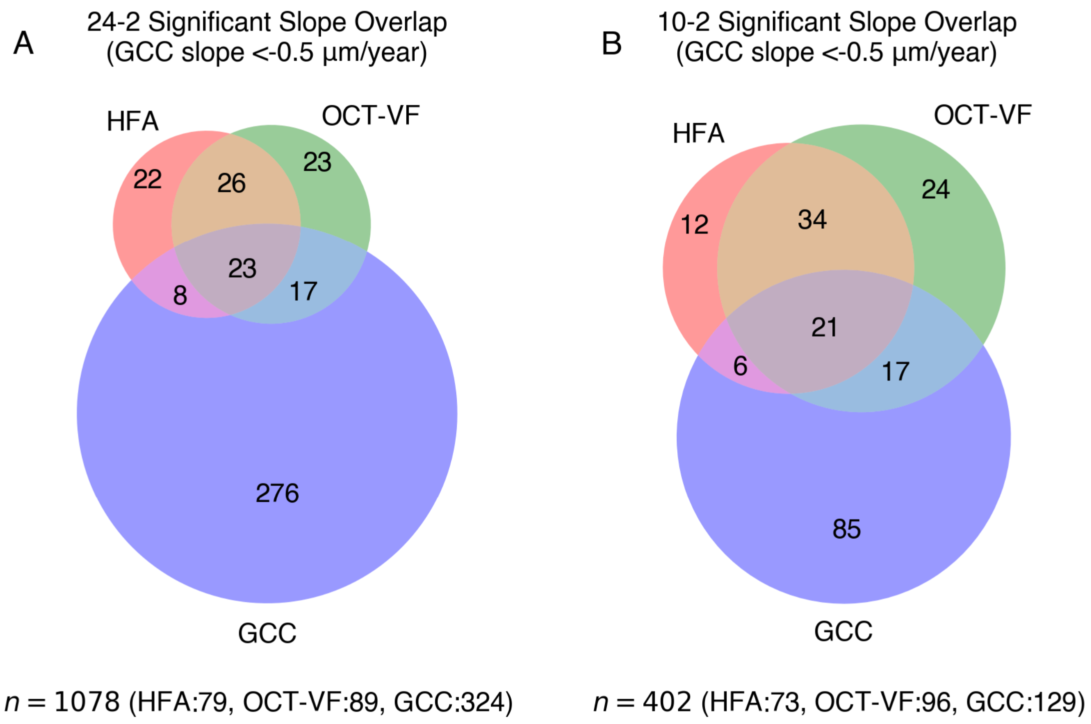

Figure S20. Overlap of eyes with significant progression by method (GCC dataset, GCC slope <–0.5 µm/year). (A) 24-2 (*n* = 1078) and (B) 10-2 (*n* = 402). Venn diagrams show the counts of eyes classified as progressing by HFA (MD slope), OCT-VF (MD slope), and GCC (macular GCC thickness slope). Progression was defined, for HFA and OCT-VF, as MD slope ≤–0.5 dB/year with two-sided $P < 0.025$ (Bonferroni across 24-2 vs 10-2), and, for GCC, as thickness slope <–0.5 µm/year with two-sided $P < 0.025$ (same correction). Numbers within each region indicate eyes meeting each criterion and their overlaps. This dataset corresponds to the single-center GCC cohort (Table S11 / Fig. S1).
HFA = Humphrey Field Analyzer; OCT = optical coherence tomography; OCT-VF = OCT-based estimated visual field; GCC = ganglion cell complex.

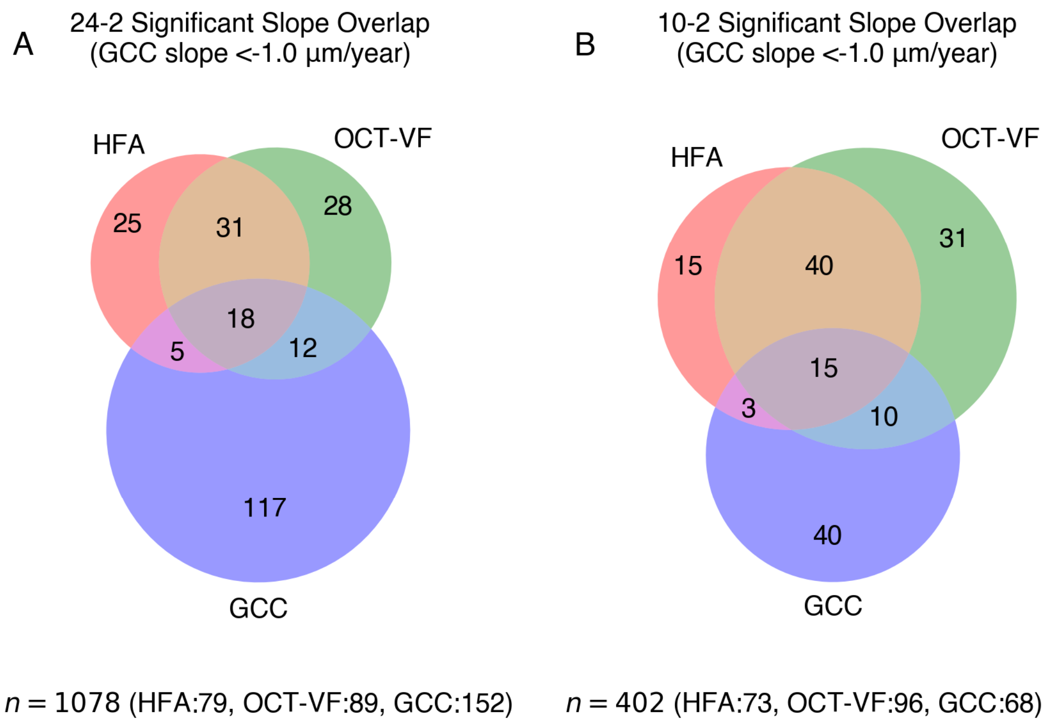

Figure S21. Overlap of eyes with significant progression by method (GCC dataset, GCC slope <–1.0 µm/year). (A) 24-2 (*n* = 1078) and (B) 10-2 (*n* = 402). Venn diagrams show the counts of eyes classified as progressing by HFA (MD slope), OCT-VF (MD slope), and GCC (macular GCC thickness slope). Progression was defined, for HFA and OCT-VF, as MD slope ≤–0.5 dB/year with two-sided $P < 0.025$ (Bonferroni across 24-2 vs 10-2), and, for GCC, as thickness slope <–1.0 µm/year with two-sided $P <$ 0.025 (same correction). Numbers within each region indicate eyes meeting each criterion and their overlaps. This dataset corresponds to the single-center GCC cohort (Table S11 / Fig. S1).
HFA = Humphrey Field Analyzer; OCT = optical coherence tomography; OCT-VF = OCT-based estimated visual field; GCC = ganglion cell complex.

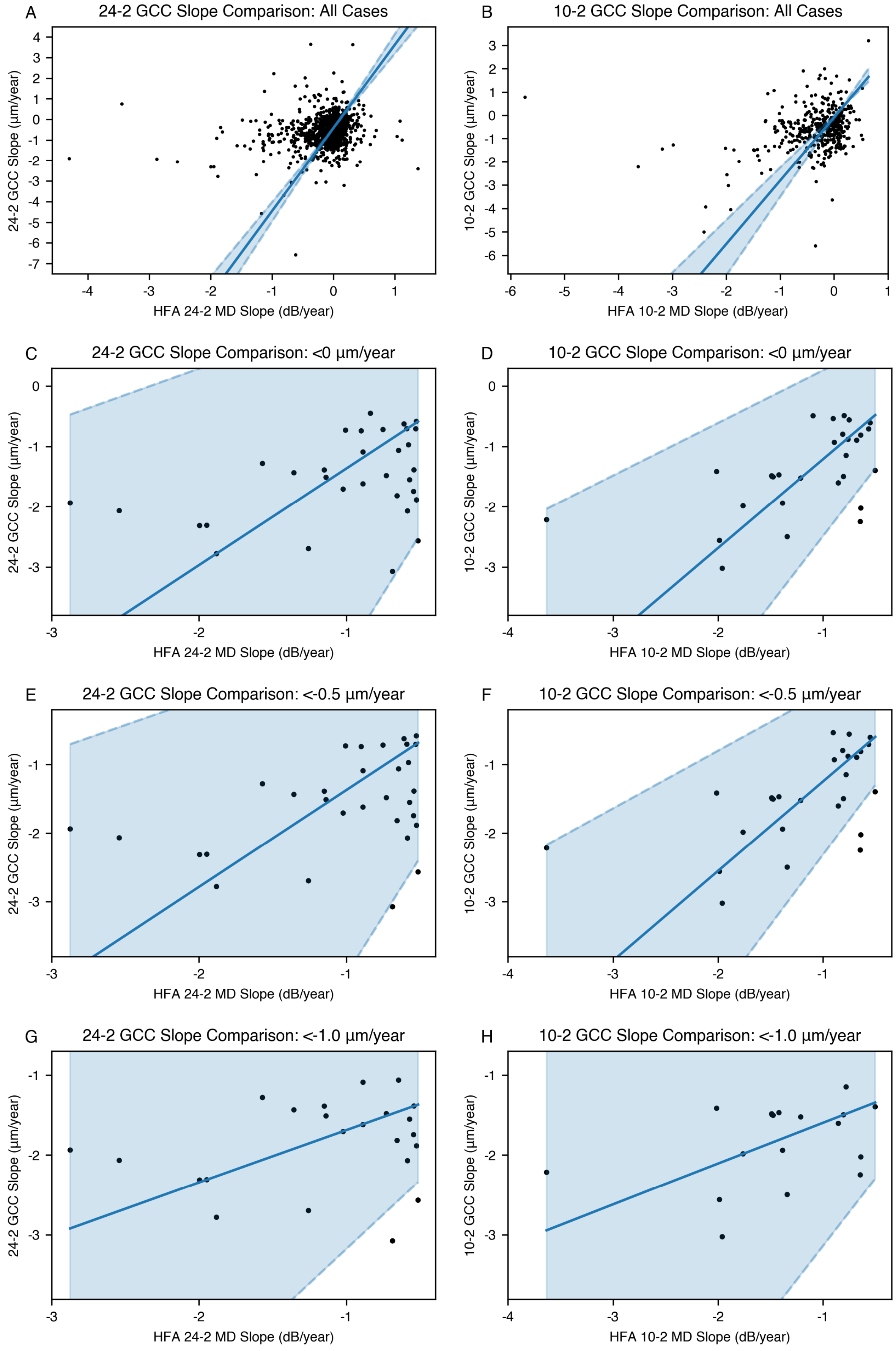

Figure S22. Association between HFA MD slope and macular GCC thickness slope. (A, B) All eyes in

the GCC dataset for 24-2 (A, n = 1078) and 10-2 (B, n = 402). (C–H) Eyes meeting concurrent progression by HFA (MD slope ≤ −0.5 dB/year, two-sided $P < 0.025$) and GCC at three thresholds: <0.0 µm/year (C, n = 32; D, n = 29), <−0.5 µm/year (E, n = 31; F, n = 27), and <−1.0 µm/year (G, n = 23; H, n = 18). The x-axis is HFA MD slope (dB/year) and the y-axis is GCC thickness slope (µm/year); points are per eye and the solid line is the Passing–Bablok fit. The light blue shaded area indicates its 95% confidence interval. Pearson's r by panel: A 0.218 ($P < 0.001$), B 0.290 ($P < 0.001$), C 0.385 ($P = 0.030$), D 0.565 ($P = 0.001$), E 0.385 ($P = 0.033$), F 0.575 ($P = 0.002$), G 0.225 ($P = 0.303$), H 0.372 ($P = 0.128$).
HFA = Humphrey Field Analyzer; MD = mean deviation; GCC = ganglion cell complex; CI = confidence interval.

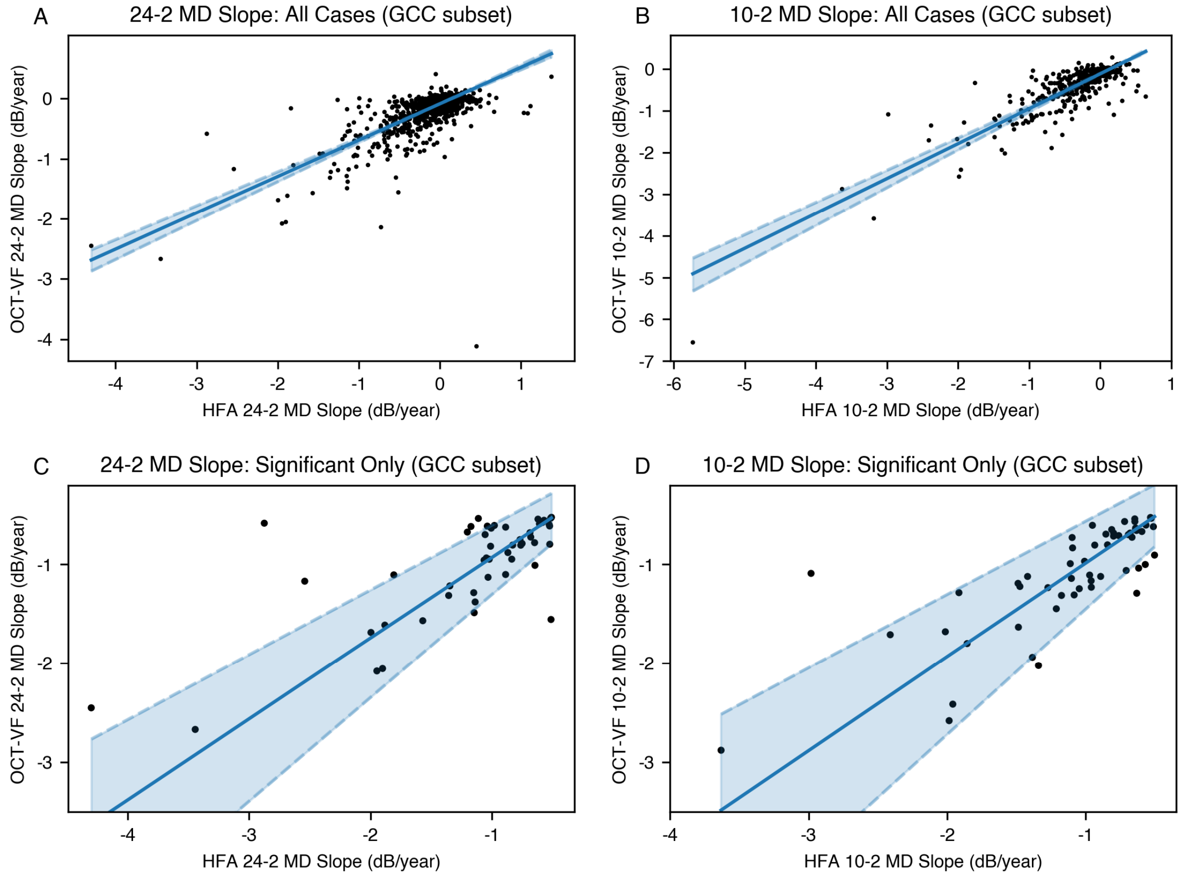

Figure S23. Association between HFA and OCT-VF MD slopes in the GCC analysis dataset. (A, B) All eyes with available GCC data for 24-2 (A, n = 1078) and 10-2 (B, n = 402). (C, D) Eyes with significant MD progression in both HFA and OCT-VF (slope ≤ −0.5 dB/year, two-sided $P < 0.025$) for 24-2 (C, $n = 49$) and 10-2 (D, $n = 55$). The x-axis shows HFA MD slope (dB/year) and the y-axis shows OCT-VF MD slope (dB/year); each point represents one eye, and the solid line denotes the Passing–Bablok fit with its 95% confidence interval shown in light blue. Passing–Bablok slopes (95% CI) and Pearson's correlation coefficients (r) were as follows: A, 0.605 (0.566–0.649), $r = 0.673$, $P < 0.001$; B, 0.837 (0.773–0.907), $r = 0.850$, $P < 0.001$; C, 0.816 (0.656–1.043), $r = 0.726$, $P < 0.001$; and D, 0.948 (0.743–1.268), $r = 0.772$, $P < 0.001$.

HFA = Humphrey Field Analyzer; OCT = optical coherence tomography; OCT-VF = OCT-based estimated visual field; MD = mean deviation; GCC = ganglion cell complex; CI = confidence interval.

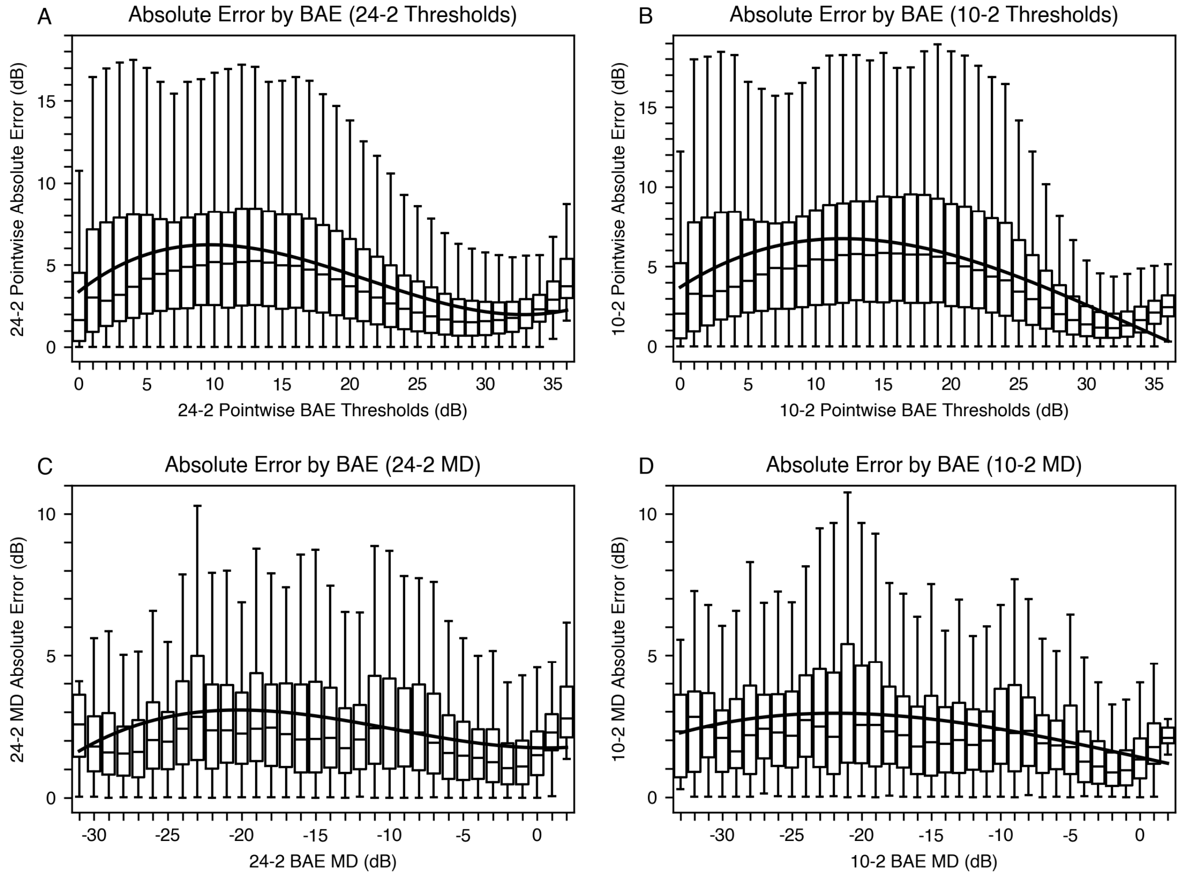

Figure S24. Relationships between VF severity and absolute errors relative to BAE. (A) 24-2 pointwise thresholds, (B) 10-2 pointwise thresholds, (C) 24-2 MD, and (D) 10-2 MD. The horizontal axis represents VF severity as determined by the BAE value, and the vertical axis shows the absolute error (dB) between the OCT-VF estimate and the corresponding BAE. Each panel displays boxplots of absolute errors for each BAE value, together with a cubic regression curve. BAE values were derived from linear regression over time for each eye. For pointwise thresholds, a separate regression line was fitted for each test location, and the predicted HFA value at each OCT scan date was used as the BAE threshold. For MD, a regression line was fitted using all longitudinal HFA measurements for each eye, and the predicted MD at each OCT scan date was used as the BAE MD. BAE values represent smoothed estimates derived from the longitudinal HFA series and are used as a reference for comparison rather than as independent measurements. OCT-VF values were generated for all available OCT scans in the longitudinal dataset, and each prediction was paired with the corresponding BAE value obtained at that same OCT date. Across all parameters, absolute error increased progressively from mild to moderately severe conditions, followed by a modest reduction in the most advanced range, resulting in a broadly convex (inverted-U–shaped) pattern.

BAE = best available estimate; MD = mean deviation; VF = visual field; OCT = optical coherence tomography; OCT-VF = OCT-based estimated visual field; HFA = Humphrey Field Analyzer.

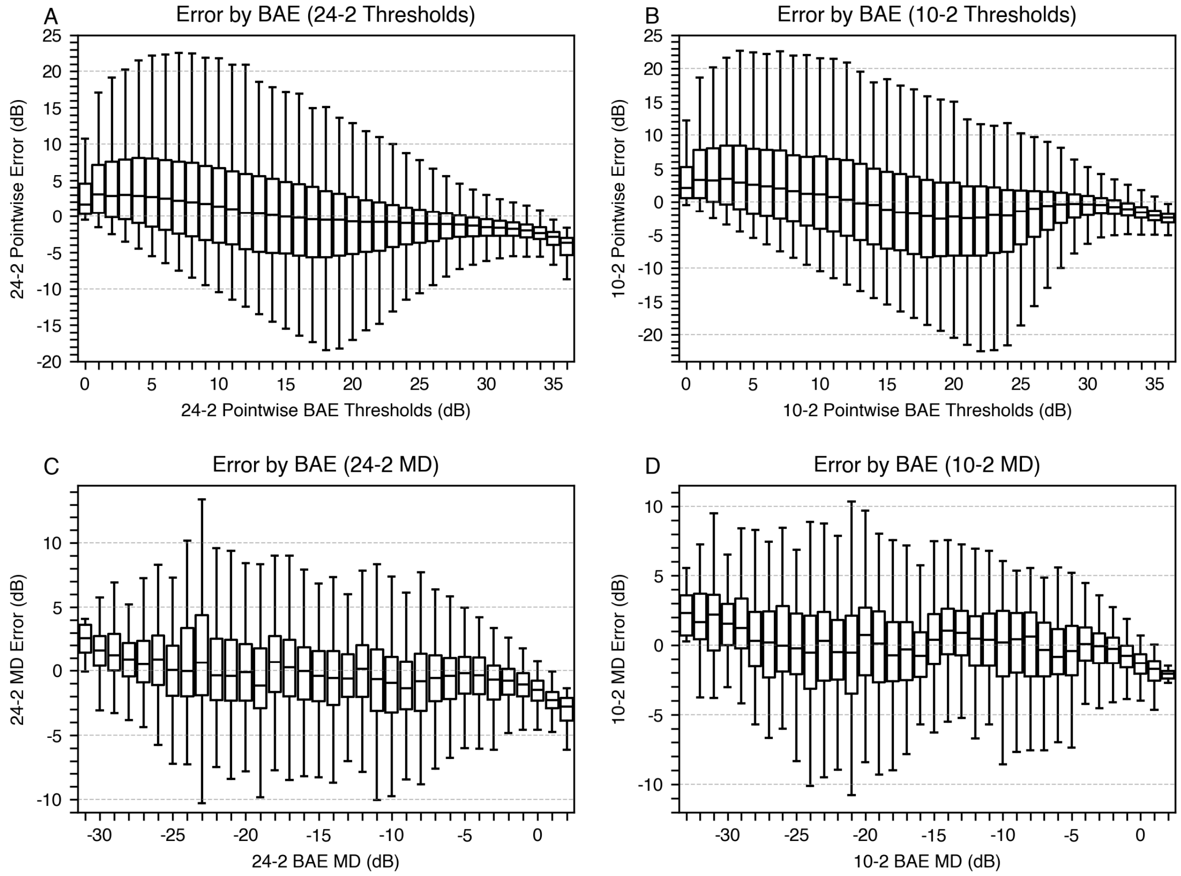

Figure S25. Relationship between VF severity and errors (OCT-VF minus BAE) relative to BAE. (A) 24-2 pointwise thresholds, (B) 10-2 pointwise thresholds, (C) 24-2 MD, and (D) 10-2 MD. The horizontal axis represents VF severity as determined by the BAE value, and the vertical axis shows the error (dB) between the OCT-VF estimate and the corresponding BAE (OCT-VF minus BAE). Each panel displays boxplots of errors for each BAE value. The plots show a tendency for OCT-VF to overestimate more severe (lower) thresholds and underestimate better (higher) thresholds, indicating a shrinkage toward the central tendency. BAE values were derived from linear regression over time for each eye. For pointwise thresholds, a separate regression line was fitted for each test location, and the predicted HFA value at each OCT scan date was used as the BAE threshold. For MD, a regression line was fitted using all longitudinal HFA measurements for each eye, and the predicted MD at each OCT scan date was used as the BAE MD. OCT-VF values were generated for all available OCT scans in the longitudinal dataset, and each prediction was paired with the corresponding BAE value obtained at that same OCT date.

BAE = best available estimate; MD = mean deviation; VF = visual field; OCT = optical coherence tomography; OCT-VF = OCT-based estimated visual field; HFA = Humphrey Field Analyzer.

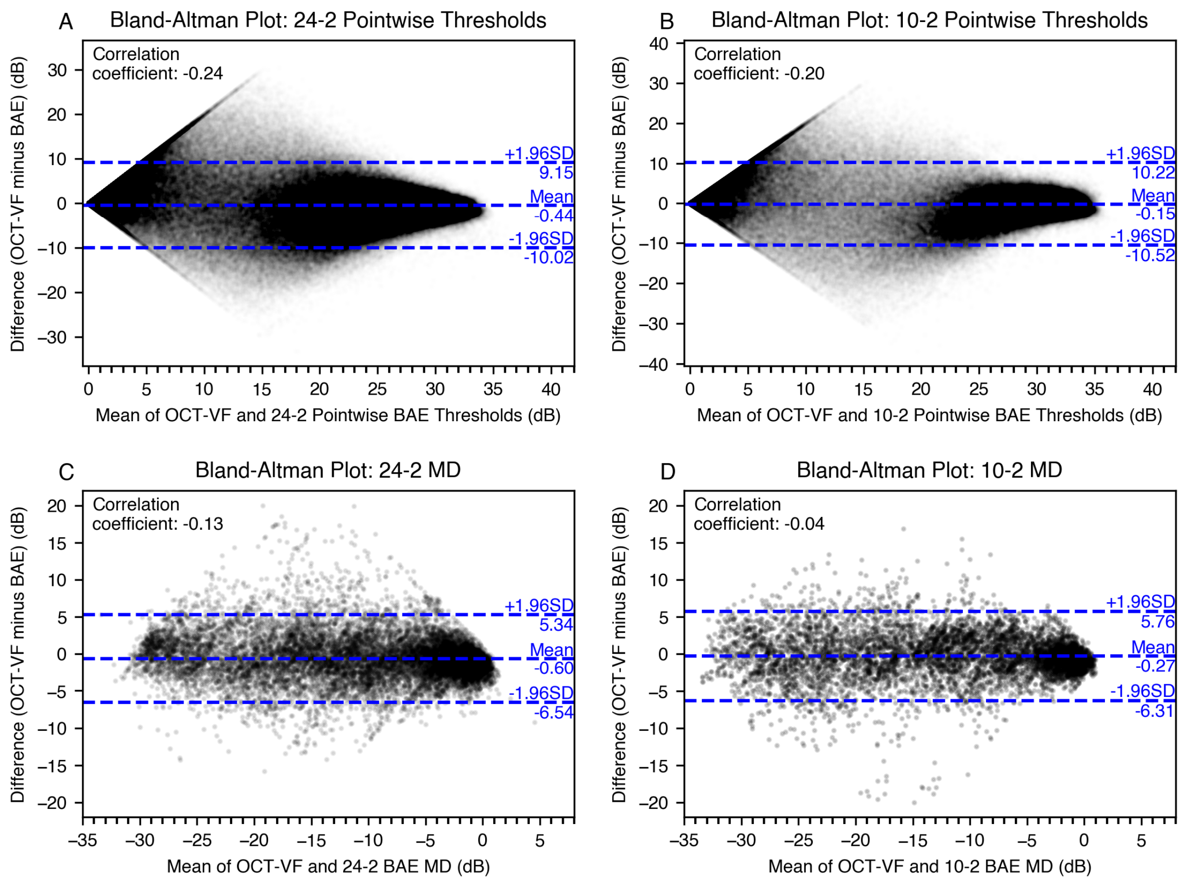

Figure S26. Bland-Altman plots for agreement between OCT-VF and BAE parameters. (A) 24-2 pointwise thresholds, (B) 10-2 pointwise thresholds, (C) 24-2 MD, and (D) 10-2 MD. The horizontal axis represents the mean of the OCT-VF and BAE values, and the vertical axis shows their difference. BAE values were derived from linear regression over time for each eye. For pointwise thresholds, a separate regression line was fitted for each test location, and the predicted HFA value at each OCT scan date was used as the BAE threshold. For MD, a regression line was fitted using all longitudinal HFA measurements for each eye, and the predicted MD at each OCT scan date was used as the BAE MD. OCT-VF values were generated for all available OCT scans in the longitudinal dataset, and each prediction was paired with the corresponding BAE value obtained on the same date. The central dashed line indicates the mean difference, and the upper and lower dashed lines represent the 95% limits of agreement (±1.96 SD). Mean differences ranged from –0.60 to –0.15 dB, and correlation coefficients ranged from –0.24 to –0.04. The 95% limits of agreement were –10.02 to 9.15 dB for 24-2 pointwise thresholds, –10.52 to 10.22 dB for 10-2 pointwise thresholds, –6.54 to 5.34 dB for 24-2 MD, and –6.31 to 5.76 dB for 10-2 MD.

VF = visual field; MD = mean deviation; OCT = optical coherence tomography; OCT-VF = OCT-based estimated visual field; HFA = Humphrey Field Analyzer; SD = standard deviation; BAE = best available estimate.

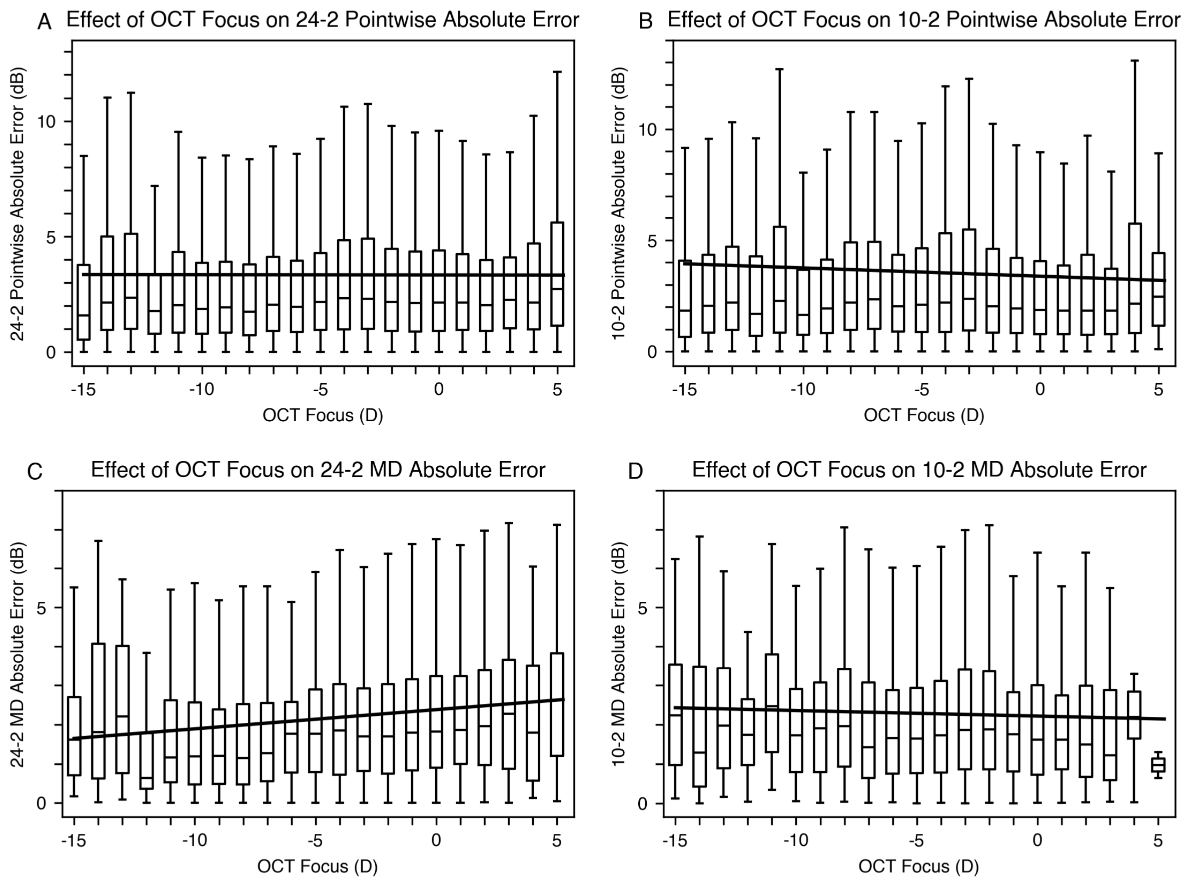

Figure S27. Effect of OCT focus (dioptric defocus) on absolute error relative to BAE. (A) 24-2 pointwise thresholds, (B) 10-2 pointwise thresholds, (C) 24-2 MD, and (D) 10-2 MD. The horizontal axis represents OCT focus values (closely corresponding to refractive error in diopters), and the vertical axis represents the absolute error (dB) between the OCT-VF estimate and the corresponding BAE. Each panel displays boxplots of absolute errors for each OCT focus value, with linear regression lines. Cluster bootstrap regression with patient-level resampling (B = 10,000) yielded slopes of –0.0001 dB/D (95% CI, –0.0292 to 0.0287) for 24-2 thresholds, –0.0315 dB/D (95% CI, –0.0734 to 0.0096) for 10-2 thresholds, 0.0481 dB/D (95% CI, 0.0160 to 0.0786) for 24-2 MD, and –0.0127 dB/D (95% CI, –0.0494 to 0.0252) for 10-2 MD. For pointwise thresholds and 10-2 MD, the 95% confidence intervals included zero, indicating no statistically detectable association between refractive error and model performance. In contrast, a small positive association was observed for 24-2 MD. BAE values were derived from linear regression over time for each eye. For pointwise thresholds, a separate regression line was fitted for each test location, and the predicted HFA value at each OCT scan date was used as the BAE threshold. For MD, a regression line was fitted using all longitudinal HFA measurements for each eye, and the predicted MD at each OCT scan date was used as the BAE MD. OCT-VF values were generated for all available OCT scans in the longitudinal dataset, and each prediction was paired with the corresponding BAE value obtained on the same date.

OCT = optical coherence tomography; VF = visual field; MD = mean deviation; D = diopter; MAE = mean absolute error.

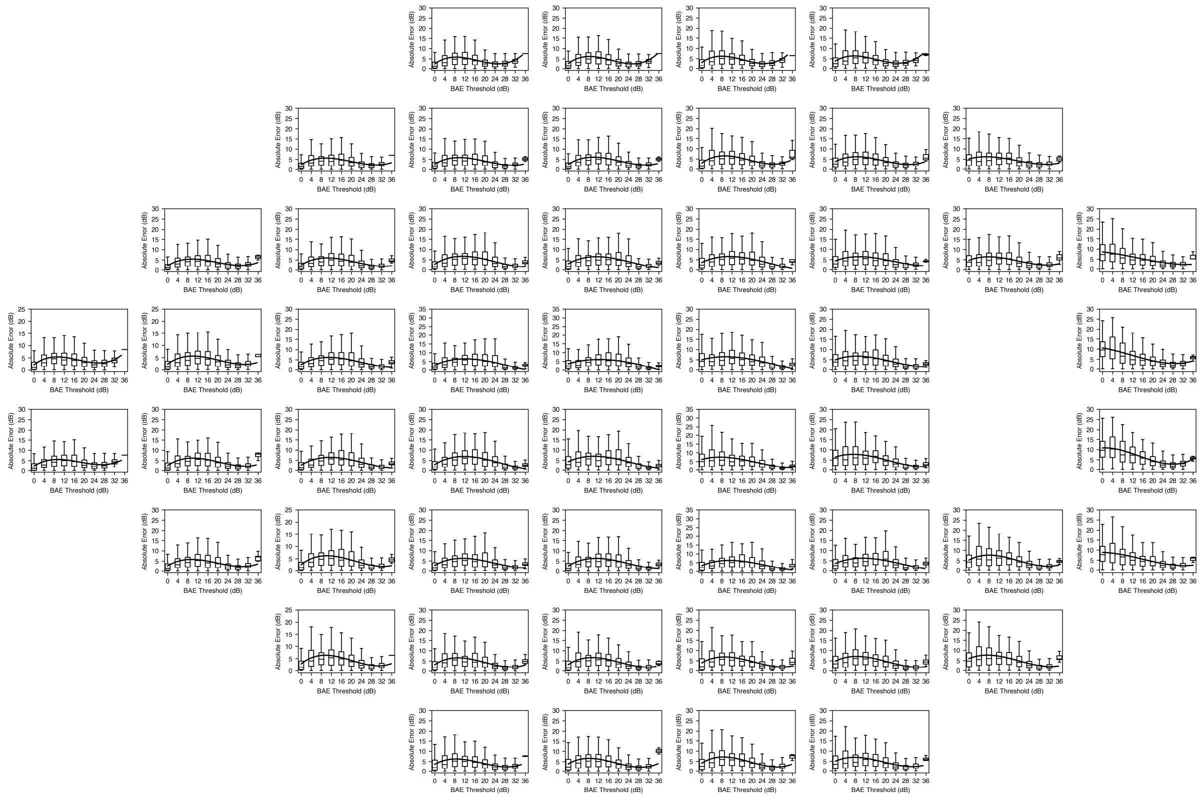

Figure S28. Spatial distribution of OCT-VF estimation accuracy across the 24-2 VF. The boxplots show absolute error for each test point, with cubic regression curves showing the relationship between BAE severity (horizontal axis) and absolute error (vertical axis). Because OCT imaging covered a 9 × 9 mm region centered on the macula—extending nasally to the optic disc—estimation accuracy exhibited clear spatial variation. In more advanced disease, nasal VF locations, where relevant retinal nerve fiber information was included within the OCT scan area, showed lower errors, whereas temporal VF locations, especially those farther from the scan coverage, showed higher errors. Data from left eyes were horizontally flipped and converted to right eye position for standardized analysis. BAE values were obtained from longitudinal linear regression for each eye: for each test location, a separate regression line was fitted using all HFA measurements, and the predicted value at each OCT scan date was used as the BAE threshold. OCT-VF predictions were generated for all available OCT scans in the dataset, and each prediction was paired with the corresponding BAE threshold measured on the same date.

BAE = best available estimate; OCT = optical coherence tomography; VF = visual field; OCT-VF = OCT-based estimated visual field.

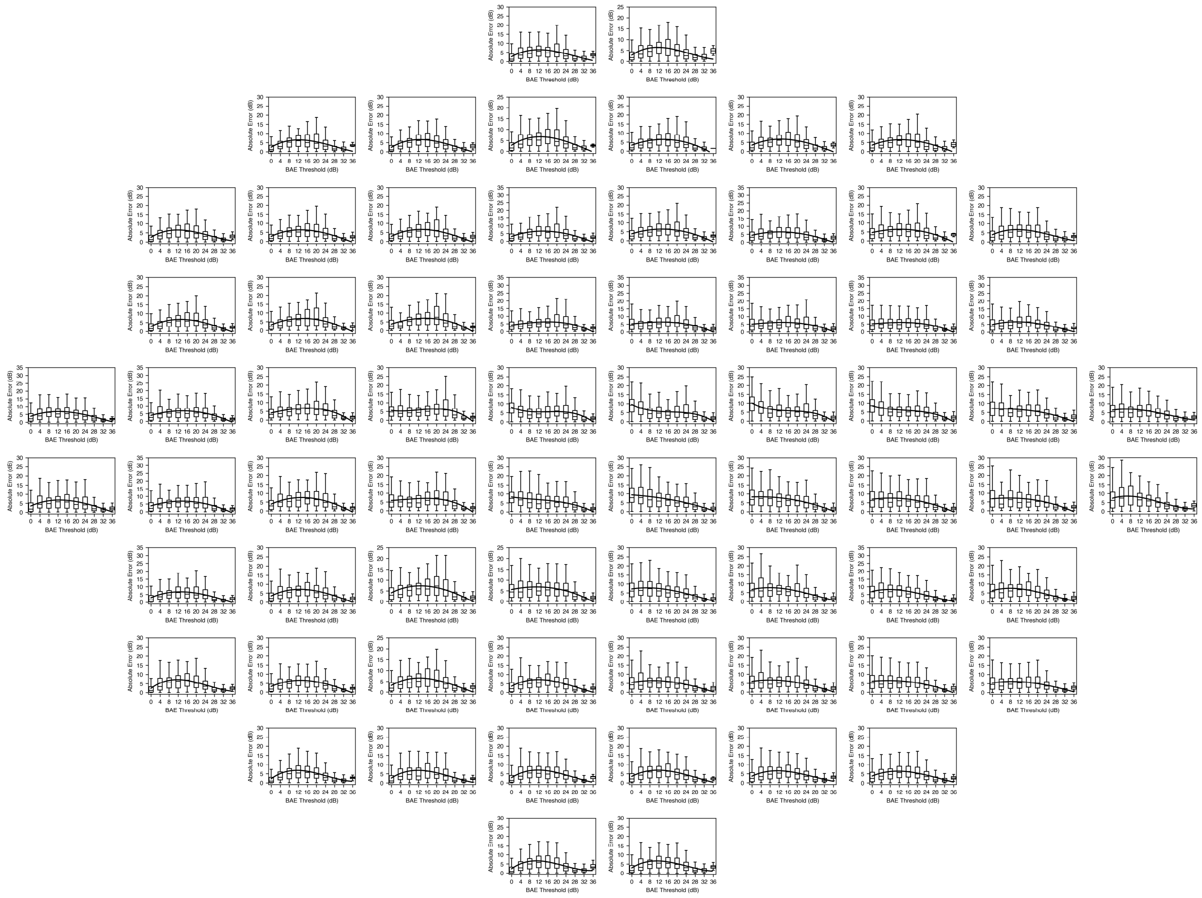

Figure S29. Spatial distribution of OCT-VF estimation accuracy across the 10-2 VF. Similar to Figure S28, these boxplots illustrate how absolute error varies with threshold values across the central VF, with cubic regression curves showing the relationship between BAE severity (horizontal axis) and absolute error (vertical axis). In more advanced disease, nasal VF locations—where retinal nerve fiber bundles contributing to central vision were well represented within the OCT scan area—tended to show slightly lower errors. In contrast, temporal VF locations closer to the optic disc exhibited slightly higher errors, likely because the relevant nerve fiber trajectories have shorter in-scan paths and provide less structural information for the model. Data from left eyes were horizontally flipped and converted to right eye position to ensure consistent spatial representation. BAE values were obtained from longitudinal linear regression for each eye: for each test location, a separate regression line was fitted using all HFA measurements, and the predicted value at each OCT scan date was used as the BAE threshold. OCT-VF predictions were generated for all available OCT scans in the dataset, and each prediction was paired with the corresponding BAE threshold measured on the same date.

BAE = best available estimate; OCT = optical coherence tomography; VF = visual field; OCT-VF = OCT-based estimated visual field.

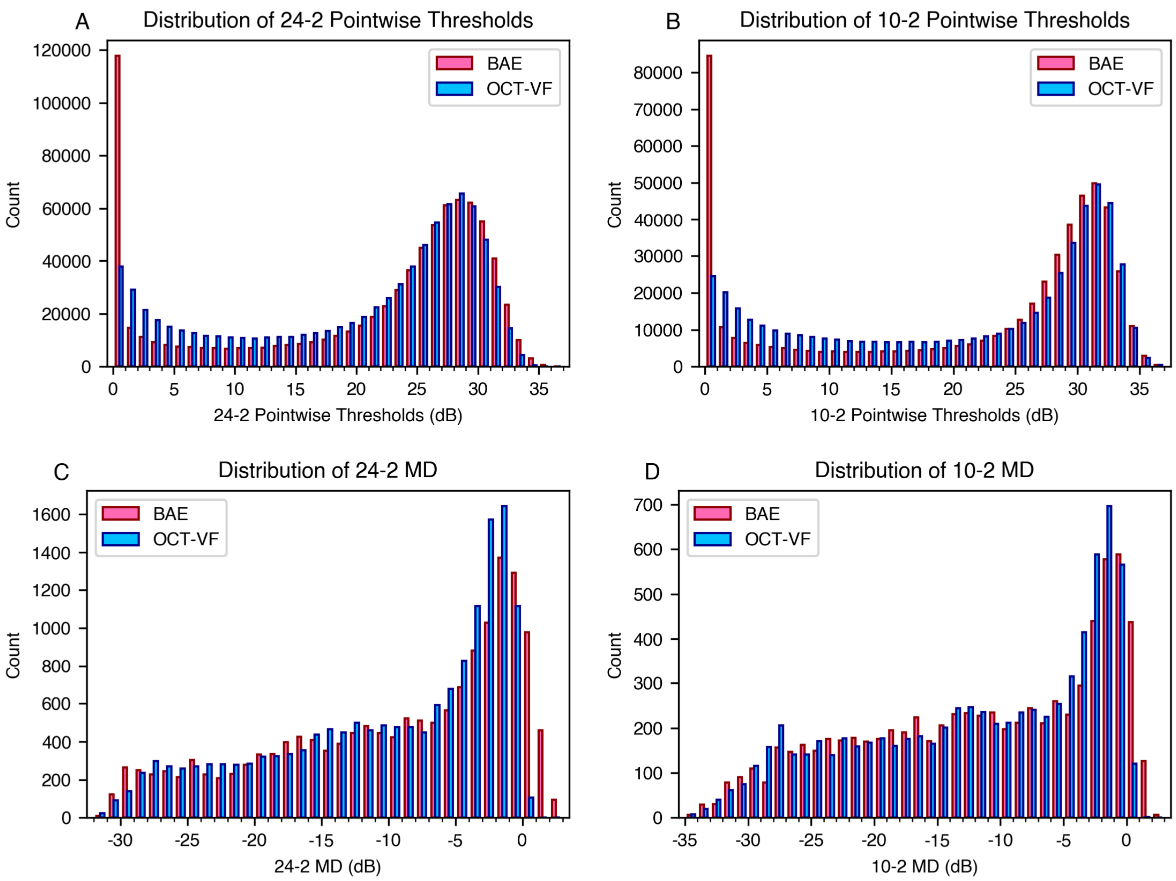

Figure S30. Population distribution by severity level using BAE and OCT-VF. (A, B) Distributions of pointwise thresholds for the 24-2 and 10-2 test patterns, respectively. (C, D) Distributions of MD for the 24-2 and 10-2 patterns. BAE values were derived from linear regression over time for each eye. For pointwise thresholds, a separate regression line was fitted for each test location, and the predicted HFA value at each OCT scan date was used as the BAE threshold. For MD, a regression line was fitted using all longitudinal HFA measurements for each eye, and the predicted MD at each OCT scan date was used as the BAE MD. OCT-VF values were generated for all available OCT scans in the longitudinal dataset, and each prediction was paired with the corresponding BAE value obtained at that same OCT date. In the threshold distributions (A, B), there is a notable discrepancy at 0 dB, with OCT-VF showing fewer measurements at this floor value compared to BAE.
MD = mean deviation; BAE = best available estimate; OCT = optical coherence tomography; OCT-VF = OCT-based estimated visual field; HFA = Humphrey Field Analyzer.

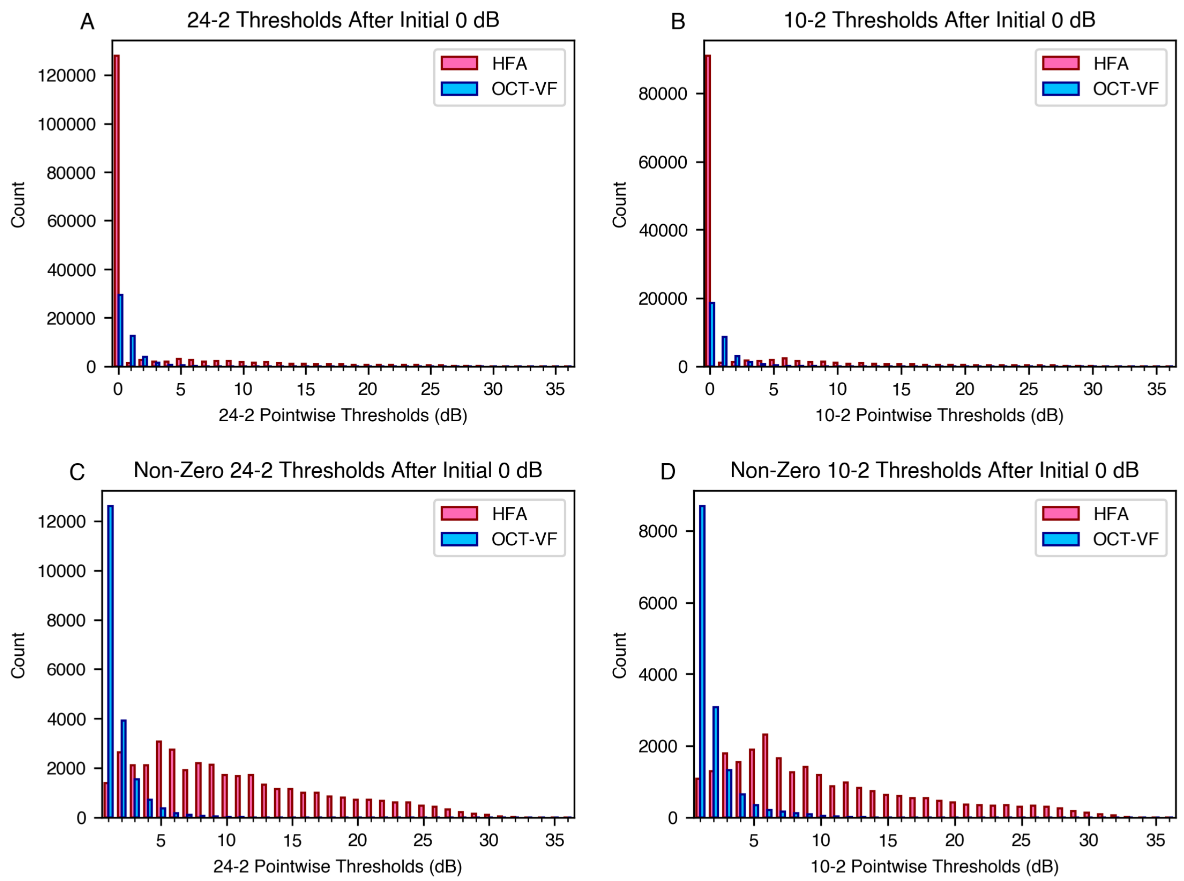

Figure S31. Longitudinal analysis of visual field pointwise thresholds following initial 0 dB measurements. (A, B) show distributions of all pointwise thresholds following initial 0 dB readings in 24-2 and 10-2 tests. HFA measurements remain at 0 dB in 77.1% (24-2) and 78.4% (10-2) of cases, compared to 60.0% (24-2) and 55.6% (10-2) for OCT-VF. (C, D) show distributions of non-zero thresholds after initial 0 dB readings in 24-2 and 10-2 tests. HFA measurements showed substantial recovery (24-2: 10.5 ± 7.1 dB, 10-2: 10.4 ± 7.4 dB), while OCT-VF concentrated at lower values (24-2: 1.7 ± 1.6 dB, 10-2: 2.0 ± 1.8 dB).

HFA = Humphrey Field Analyzer; OCT = optical coherence tomography; OCT-VF = OCT-based estimated visual field.

Table S1. Comparison of MAE for Forecasting the Last HFA across Methods and Endpoints

**Metric**	**Prediction Method**	**MAE ± SD (dB)**	**ΔMAE vs. HFA-RF (95% CI)**	**Raw *P* value**	**Adjusted *P* value**
24-2 Pointwise Threshold	OCT-RF	4.02 ± 2.38	1.34 (1.24, 1.44)	< 0.001	< 0.001
	AOS	2.56 ± 1.74	–0.11 (–0.16, –0.07)	< 0.001	< 0.001
	AOS-AVG	2.45 ± 1.65	–0.23 (–0.26, –0.21)	< 0.001	< 0.001
	HFA-RF	2.68 ± 1.74			
10-2 Pointwise Threshold	OCT-RF	4.04 ± 2.36	1.39 (1.23, 1.53)	< 0.001	< 0.001
	AOS	2.55 ± 1.55	–0.10 (–0.16, –0.04)	0.002	0.002
	AOS-AVG	2.44 ± 1.48	–0.22 (–0.26, –0.19)	< 0.001	< 0.001
	HFA-RF	2.65 ± 1.60			
24-2 MD	OCT-RF	2.65 ± 2.61	1.24 (1.11, 1.36)	< 0.001	< 0.001
	AOS	1.51 ± 1.95	0.09 (0.03, 0.16)	0.006	0.006
	AOS-AVG	1.32 ± 1.80	–0.10 (–0.14, –0.06)	< 0.001	< 0.001
	HFA-RF	1.42 ± 1.86			
10-2 MD	OCT-RF	2.52 ± 2.48	1.19 (1.01, 1.37)	< 0.001	< 0.001
	AOS	1.42 ± 1.65	0.09 (0.00, 0.18)	0.053	0.053
	AOS-AVG	1.25 ± 1.49	–0.08 (–0.13, –0.03)	0.001	0.002
	HFA-RF	1.33 ± 1.57			

HFA = Humphrey Field Analyzer; OCT = optical coherence tomography; OCT-VF = OCT-based estimated visual field; MD = mean deviation; MAE = mean absolute error; CI = confidence interval; OCT-RF = OCT-VF regression forecast; AOS = HFA-anchored OCT-VF slope forecast; HFA-RF = HFA regression forecast.

OCT-RF (all OCT-VF tests prior to the last HFA fitted with a linear trend and extrapolated to the last HFA timepoint); AOS (mean of all HFA tests prior to the last HFA used as the intercept anchor, with the OCT-VF slope prior to the last HFA used for extrapolation); AOS-AVG (HFA-anchored intercept defined by the mean of prior HFA measurements combined with the average of OCT-VF–derived and HFA–derived slopes estimated prior to the last HFA); HFA-RF (linear trend using all HFA tests prior to the last HFA extrapolated to the last HFA). Lower values indicate better predictions. ΔMAE denotes (method – HFA-RF) with patient-level paired bootstrap 95% CIs (B = 10,000). Adjusted P values were computed using the Holm step-down procedure within each endpoint. For reference, Bonferroni-corrected significance was defined as $P < 0.0125$. Raw *P* values are unadjusted bootstrap *P* values. Sample sizes: for 24-2 endpoints, $n = 1785$; for 10-2 endpoints, $n = 934$ (identical for pointwise and MD). Values were rounded to two decimals (*P* values to three decimals). Values for HFA forecasting conditions are identical to those shown in Table 3 and are repeated here for clarity of method-wise comparisons.

Table S2. Decomposition of Squared Error (dB²) for Forecasting the Last Pointwise HFA

**Pattern**	**Eye-offset**	**Location-fixed**	**Remainder**	**Total**
24-2	10.9 [9.3, 12.7] (30.4%)	11.2 [10.5, 11.9] (31.2%)	13.8 [13.0, 14.8] (38.4%)	35.9 [33.7, 38.3]
10-2	9.7 [7.9, 11.9] (25.4%)	13.1 [11.7, 14.4] (34.3%)	15.4 [14.2, 16.7] (40.3%)	38.2 [35.2, 41.3]

HFA = Humphrey Field Analyzer; OCT = optical coherence tomography; OCT-VF = OCT-based estimated visual field; MD = mean deviation; AOS = HFA-anchored OCT-VF slope forecast. Values are bootstrap means with 95% confidence intervals (patient-level resampling, B = 10,000). Percentages indicate each component's share of the total MSE. Eye-offset: error remaining before MD-centering, representing eye-level intercept bias (e.g., pressing/conservative responses). Location-fixed: error after MD-centering and before AOS anchoring, capturing location-specific systematic bias. Remainder: error after AOS anchoring (HFA-anchored intercept + OCT-VF slope), reflecting residual variance arising from HFA measurement variability at low sensitivities and OCT-VF slope estimation error.

Table S3. Residual Variability Comparison: OCT-VF vs. HFA

Variability Metric	OCT-VF Mean ± SD (dB)*	HFA Mean ± SD (dB)*	Difference† (95% CI, dB)	*P* value	Working Correlation‡
24-2 Pointwise Threshold	1.09 ± 0.92	2.39 ± 2.21	–1.31 (–1.35, –1.26)	< 0.001	0.39
10-2 Pointwise Threshold	1.19 ± 1.10	2.37 ± 2.48	–1.18 (–1.24, –1.11)	< 0.001	0.38
24-2 MD	0.79 ± 0.67	1.13 ± 0.81	–0.35 (–0.38, -0.31)	< 0.001	0.37
10-2 MD	0.87 ± 0.77	1.07 ± 0.84	–0.20 (–0.26, –0.15)	< 0.001	0.41

MD = mean deviation; OCT = optical coherence tomography; OCT-VF = OCT-based estimated visual field; HFA = Humphrey Field Analyzer; CI = confidence interval.

*The values for OCT-VF and HFA represent the residual variability calculated using jackknife resampling, measuring absolute deviations from the regression line for each method; lower values indicate less variability and better measurement consistency.

†The Difference column represents the difference between OCT-VF and HFA residual variability (OCT-VF minus HFA) calculated using generalized estimating equations (GEE), adjusting for follow-up duration, the number of longitudinal examinations per eye, age, clustering by eye and patient, and measurement point location for threshold analyses only. Negative values indicate lower variability in OCT-VF.

‡Working correlation represents the correlation between measurements from the same eye in the GEE model.

Table S4. Permutation-based False-positive Rates for MD Progression Detection.

Modality	Target	*n*	False-Positive Rate (mean ± SD)
HFA 24-2	MD	1785	0.00727 ± 0.00201
OCT-VF 24-2	MD	1785	0.00560 ± 0.00170
HFA 10-2	MD	934	0.00750 ± 0.00279
OCT-VF 10-2	MD	934	0.00589 ± 0.00250

MD = mean deviation; HFA = Humphrey Field Analyzer; OCT = optical coherence tomography; OCT-VF = OCT-based estimated visual field; SD = standard deviation.

Within-eye permutation tests were performed to estimate the false-positive rate of progression detection under the null hypothesis of no temporal structure. For each eye, the order of longitudinal measurements was randomly shuffled 1,000 times, slopes and two-sided P values were recalculated, and progression was defined as slope ≤−0.5 dB/year with $P < 0.025$. The false-positive rate represents the proportion of permuted series that met these criteria despite the temporal order being randomized.

Table S5. Performance of OCT-VF for Detecting MD Progression (Reference Standard: HFA)

**Test Pattern**	**Sensitivity**	**Specificity**	**Accuracy**	**Cohen's κ**	***n***
24-2	0.461	0.917	0.845	0.393	1785
10-2	0.396	0.901	0.815	0.312	934

MD = mean deviation; OCT-VF = OCT-based estimated visual field; HFA = Humphrey Field Analyzer; SITA = Swedish Interactive Threshold Algorithm.
Reference standard: HFA progression detection (slope ≤–0.5 dB/year, $P < 0.025$).

Table S6. Confusion Matrices for OCT-VF Detection of MD Progression (Reference Standard: HFA)

Test Pattern	True Positive	False Negative	False Positive	True Negative	Total
24-2	129	151	125	1380	1785
10-2	63	96	77	698	934

MD = mean deviation; OCT = optical coherence tomography; OCT-VF = OCT-based estimated visual field; HFA = Humphrey Field Analyzer; SITA = Swedish Interactive Threshold Algorithm. Counts are eyes. The table compares OCT-VF progression calls with HFA as the reference standard for the 24-2 and 10-2 test patterns. For both HFA and OCT-VF, progression was defined as MD slope ≤–0.5 dB/year with two-sided $P < 0.025$.

Table S7. Comparison of HFA MD Residual Variability (Absolute Residuals) between FP and TN Eyes.

Condition	FP (mean ± SD)*	TN (mean ± SD)*	*P* value
24-2	1.51 ± 1.12 (n = 125)	1.08 ± 0.76 (n = 1380)	< 0.001
10-2	1.30 ± 0.86 (n = 77)	1.05 ± 0.88 (n = 698)	< 0.001

HFA = Humphrey Field Analyzer; MD = mean deviation; FP = false-positive; TN = true-negative; OCT = optical coherence tomography; OCT-VF = OCT-based estimated visual field.

*The values for FP and TN represent the residual variability calculated using jackknife resampling, where absolute deviations from the regression line were averaged across jackknife iterations. Lower residual values indicate reduced measurement variability and better longitudinal consistency. FP and TN eyes were defined using HFA-based progression criteria (MD slope ≤–0.5 dB/year and $P < 0.025$), with OCT-VF progression evaluated using identical thresholds. FP represented OCT-VF+, HFA–, whereas TN represented OCT-VF–, HFA– (see Table S6 for full confusion matrices). For both 24-2 and 10-2, FP eyes showed significantly greater residual variability than TN eyes (all $P < 0.001$, Mann–Whitney U).

Table S8. Comparison of HFA MD Slopes between False-positive and True-negative Eyes.

Condition	FP (mean ± SD, dB/year)*	TN (mean ± SD, dB/year)*	*P* value
24-2	–0.412 ± 0.661 (n = 125)	–0.147 ± 0.565 (n = 1380)	< 0.001
10-2	–0.437 ± 0.478 (n = 77)	–0.183 ± 0.613 (n = 698)	< 0.001

HFA = Humphrey Field Analyzer; MD = mean deviation; FP = false-positive; TN = true-negative; OCT = optical coherence tomography; OCT-VF = OCT-based estimated visual field.

*Values represent the estimated HFA MD slopes obtained from longitudinal linear regression. FP and TN eyes were defined using the same progression criteria as in Table S7 (MD slope ≤–0.5 dB/year and $P < 0.025$ with HFA as the reference standard; OCT-VF progression was assessed using identical thresholds). FP corresponded to OCT-VF+, HFA–, and TN to OCT-VF–, HFA– (see Table S6 for full confusion matrices). For both 24-2 and 10-2, FP eyes exhibited significantly more negative MD slopes than TN eyes (all $P < 0.001$, Mann–Whitney U).

Table S9. Time to Detect MD Progression (Days) for Eyes Significant in Both Modalities

Pattern	HFA (mean ± SD, days)	OCT-VF (mean ± SD, days)	Δ (OCT-VF – HFA) mean (95% CI, days)	*P* value	*n*
24-2	1755 ± 820	1721 ± 818	−88 (−224, 39)	0.190	129
10-2	1349 ± 472	1407 ± 632	33 (−70, 137)	0.535	63

MD = mean deviation; OCT-VF = OCT-based estimated visual field; HFA = Humphrey Field Analyzer; CI = confidence interval.
This table summarizes, for the 24-2 (n = 129) and 10-2 (n = 63) patterns, the minimum observation period per eye required to reach the prespecified statistical criterion for a negative MD slope (slope ≤–0.5 dB/year with two-sided $P < 0.025$) for HFA and OCT-VF. Values are bootstrap means with 95% confidence intervals derived from patient-level paired resampling (B = 10,000) and are rounded to the nearest day; "mean ± SD" reflects dispersion across eye-level observations. The contrast Δ (OCT-VF – HFA) represents the bootstrap-estimated mean difference in time to reach the progression criterion. Because patient-level paired resampling accounts for within-patient correlation, Δ may differ from the arithmetic difference of the reported means. Positive values indicate earlier detection by HFA. No statistically significant differences were observed between modalities for either the 24-2 or 10-2 patterns.

Table S10. Comparison of MD Progression Slopes Between OCT-VF and HFA

**Analysis Group**	**OCT-VF MD Slope (Mean ± SD, dB/year)**	**HFA MD Slope (Mean ± SD, dB/year)**	**Δ MD Slope (OCT-VF – HFA, 95% CI, dB/year)**	***P* value**	***n***
24-2 all eyes	–0.316 ± 0.552	–0.325 ± 0.708	0.010 (–0.023, 0.043)	0.557	1785
10-2 all eyes	–0.359 ± 0.632	–0.393 ± 0.764	0.034 (–0.017, 0.083)	0.185	934
24-2 significant progressors*	–1.17 ± 0.71	–1.23 ± 0.73	0.059 (–0.015, 0.134)	0.117	129
10-2 significant progressors*	–1.36 ± 0.77	–1.39 ± 0.66	0.024 (–0.129, 0.169)	0.755	63

MD = mean deviation; OCT-VF = OCT-based estimated visual field; HFA = Humphrey Field Analyzer; SD = standard deviation; CI = confidence interval.

MD slopes were calculated from linear regression over time for each eye. Differences in mean MD slopes between OCT-VF and HFA were evaluated using patient-wise bootstrap resampling (10,000 iterations) to estimate the 95% confidence intervals (CIs) and two-sided *P* values. Δ MD slope represents the mean difference (OCT-VF – HFA) in progression rate. No statistically significant differences in MD slopes were observed between OCT-VF and HFA across analysis groups. Accordingly, the hypothesis that OCT-VF systematically underestimates progression rates was not supported.

*Significant progressors are defined as eyes with $P < 0.025$ and slope ≤-0.5 dB/year in both OCT-VF and HFA methods.

Table S11. Characteristics of the Macular GCC Analysis Dataset (Paired OCT and HFA Records).

**Characteristics**	**OCT (24-2)**	**HFA (24-2)**	**OCT (10-2)**	**HFA 10-2**
Number of patients	639	639	256	256
Number of eyes	1078	1078	402	402
Age (years)	63.1 ± 11.8	63.1 ± 11.8	66.3 ± 10.7	66.3 ± 10.7
Mean MD (dB)	–3.28 ± 4.28*	–3.12 ± 4.77	–6.08 ± 5.67*	–5.88 ± 5.82
Number of tests	8.83 ± 2.75	8.83 ± 2.75	8.88 ± 3.10	8.88 ± 3.10
Follow-up duration (days)	3160 ± 940	3159 ± 938	2909 ± 943	2912 ± 950

GCC = ganglion cell complex; OCT = optical coherence tomography; OCT-VF = OCT-based estimated visual field; HFA = Humphrey Field Analyzer; MD = mean deviation.

Columns "OCT (24-2)" and "OCT (10-2)" refer to OCT-VF and GCC values derived from the same OCT acquisitions; the corresponding HFA columns are the temporally matched visual-field records from the same eyes and patients. Values are means ± standard deviations. Differences in follow-up duration reflect that OCT and HFA examinations were not always performed on identical dates, despite the temporal matching criteria (≤90-day differences). This GCC analysis dataset was obtained from a single center. Details of dataset construction are provided in the Supplementary Methods.

*MD values for OCT-VF are estimates derived from the OCT-VF models.

Table S12. Permutation-based False-positive Rates for Progression Detection (GCC Analysis Dataset)

**Modality**	**Target**	***n* eyes**	**False-Positive Rate (mean ± SD)**
HFA 24-2	MD	1078	0.00244 ± 0.00148
OCT-VF 24-2	MD	1078	0.00153 ± 0.00120
GCC 24-2 (<0.0 µm/year)	GCC	1078	0.0137 ± 0.00353
GCC 24-2 (<–0.5 µm/year)	GCC	1078	0.01064 ± 0.00306
GCC 24-2 (<–1.0 µm/year)	GCC	1078	0.00473 ± 0.00201
HFA 10-2	MD	402	0.00445 ± 0.00321
OCT-VF 10-2	MD	402	0.00456 ± 0.00339
GCC 10-2 (<0.0 µm/year)	GCC	402	0.0126 ± 0.00540
GCC 10-2 (<–0.5 µm/year)	GCC	402	0.01084 ± 0.00504
GCC 10-2 (<–1.0 µm/year)	GCC	402	0.00617 ± 0.00390

GCC = ganglion cell complex; HFA = Humphrey Field Analyzer; OCT = optical coherence tomography; OCT-VF = OCT-based estimated visual field; MD = mean deviation; SD = standard deviation.

Within-eye permutation tests were performed to estimate the false-positive rate of progression detection under the null hypothesis of no temporal structure in the GCC analysis dataset. For each eye, the temporal order of all longitudinal measurements was randomly shuffled 1,000 times, and slopes and two-sided P values were recalculated using simple linear regression. Progression criteria matched the main analyses for each modality: for HFA and OCT-VF, progression was defined as an MD slope ≤–0.5 dB/year with $P < 0.025$; for GCC, progression was evaluated using three slope thresholds (<0.0, <–0.5, and <–1.0 µm/year) with $P < 0.025$.

Table S13. Confusion Matrices for Detecting MD Progression using HFA as the Reference (GCC Analysis Dataset)

Dataset	Test Pattern	True Positive	False Negative	False Positive	True Negative	Total
OCT-VF	24-2	49	30	40	959	1078
GCC (<0.0 µm/year)	24-2	32	47	344	655	1078
GCC (<–0.5 µm/year)	24-2	31	48	293	706	1078
GCC (<–1.0 µm/year)	24-2	23	56	129	870	1078
OCT-VF	10-2	55	18	41	288	402
GCC (<0.0 µm/year)	10-2	29	44	118	211	402
GCC (<–0.5 µm/year)	10-2	27	46	102	227	402
GCC (<–1.0 µm/year)	10-2	18	55	50	279	402

MD = mean deviation; OCT = optical coherence tomography; OCT-VF = OCT-based estimated visual field; GCC = ganglion cell complex; HFA = Humphrey Field Analyzer.

Counts are eyes. Each confusion matrix compares a method's MD-progression calls with HFA as the reference standard. For HFA and OCT-VF, progression was defined as MD slope ≤–0.5 dB/year with two-sided $P < 0.025$; for GCC, progression was defined by the stated GCC thickness slope threshold (<0.0, <–0.5, or <–1.0 µm/year, all with two-sided $P < 0.025$). This dataset corresponds to the single-center GCC cohort (Table S11 / Fig. S1).

Table S14. Performance Metrics for MD Progression Detection: OCT-VF vs. GCC (GCC Analysis Dataset)

Method	Sensitivity	Specificity	Accuracy	Cohen's κ	Δ κ vs. OCT-VF (95% CI)
OCT-VF 24-2	0.620	0.960	0.935	0.548	
GCC 24-2 (<0.0 µm/year)	0.405	0.656	0.637	0.022	0.524 (0.423, 0.618) *
GCC 24-2 (<–0.5 µm/year)	0.392	0.707	0.684	0.041	0.505 (0.403, 0.603) *
GCC 24-2 (<–1.0 µm/year)	0.291	0.871	0.828	0.114	0.433 (0.323, 0.540) *
OCT-VF 10-2	0.753	0.875	0.853	0.560	
GCC 10-2 (<0.0 µm/year)	0.397	0.641	0.597	0.028	0.530 (0.397, 0.660) *
GCC 10-2 (<–0.5 µm/year)	0.370	0.690	0.632	0.046	0.512 (0.372, 0.647) *
GCC 10-2 (<–1.0 µm/year)	0.247	0.848	0.739	0.097	0.461 (0.317, 0.598) *

MD = mean deviation; OCT = optical coherence tomography; OCT-VF = OCT-based estimated visual field; GCC = ganglion cell complex; HFA = Humphrey Field Analyzer; CI = confidence interval.
Performance metrics are computed using HFA as the reference standard. For HFA and OCT-VF, progression was defined as MD slope ≤–0.5 dB/year with two-sided $P < 0.025$; for GCC, progression was defined by the stated GCC thickness-slope threshold (<0.0, <–0.5, or <–1.0 µm/year), each with two-sided $P < 0.025$. This dataset corresponds to the single-center GCC cohort (Table S11 / Fig. S1). Δ Cohen's κ shows the difference (OCT-VF minus GCC); values are bootstrap means with 95% CIs from patient-level paired resampling (B = 10,000).
*Two-sided *P* values for Δ from the paired bootstrap; all shown comparisons are $P < 0.001$.

Table S15. Correlations between Structural/functional Slopes and HFA MD Slope in the GCC Analysis Dataset (All Eyes)

Test pattern	Metric	*n*	Correlation (OCT-VF, HFA)[*]	Correlation (GCC, HFA)[*]	Δ Correlation (95% CI)[†]
24-2	Pearson's *r*	1078	0.673 ($P < 0.001$)	0.218 ($P < 0.001$)	0.457 (0.295, 0.593), $P < 0.001$
	Spearman's ρ	1078	0.581 ($P < 0.001$)	0.266 ($P < 0.001$)	0.314 (0.245, 0.386), $P < 0.001$
10-2	Pearson's *r*	402	0.850 ($P < 0.001$)	0.290 ($P < 0.001$)	0.549 (0.351, 0.772), $P < 0.001$
	Spearman's ρ	402	0.731 ($P < 0.001$)	0.234 ($P < 0.001$)	0.497 (0.388, 0.613), $P < 0.001$

MD = mean deviation; OCT = optical coherence tomography; OCT-VF = OCT-based estimated visual field; HFA = Humphrey Field Analyzer; GCC = ganglion cell complex; CI = confidence interval. Correlation analyses were performed in the GCC analysis dataset, a single-center dataset containing temporally matched HFA, OCT-VF, and macular GCC measurements. All eyes were included (i.e., analyses were conducted before applying any significance criteria), using one MD slope value per eye for HFA and OCT-VF, and one GCC thickness slope per eye.

[*]For each test pattern (24-2 and 10-2), Pearson's correlation coefficient (*r*) and Spearman's rank correlation coefficient (ρ) were calculated between OCT-VF MD slope and HFA MD slope, and between GCC slope and HFA MD slope.

[†]To directly compare OCT-VF with GCC, the difference in correlation strength was defined as:

Δ Correlation = *r* (OCT-VF, HFA) – *r* (GCC, HFA) for Pearson's *r*,

and

Δ Correlation = ρ (OCT-VF, HFA) – ρ (GCC, HFA) for Spearman's ρ,

and evaluated using patient-level cluster bootstrap resampling (10,000 iterations) to account for within-patient clustering. Positive Δ values indicate that OCT-VF slopes align more closely with HFA MD slopes than GCC slopes do. Two-sided *P* values were derived from the bootstrap distribution, and all reported *P* values < 0.001 denote strong statistical significance.

Table S16. Severity-stratified Correlations between Progression Rates and HFA MD Slopes (GCC Analysis Dataset)

**Test pattern**	**Severity group**[*]	***n***	***r* (OCT-VF, HFA)**	***r* (GCC, HFA)**	**Δ *r* (95% CI)**[†]
24-2	MD ≥−6 dB	896	0.536	0.246	0.289 (0.193, 0.398)
	−12≤ MD <−6 dB	107	0.674	0.253	0.408 (0.192, 0.596)
	MD <−12 dB	75	0.462	0.146	0.346 (0.211, 0.880)
10-2	MD ≥−6 dB	254	0.732	0.263	0.469 (0.353, 0.592)
	−12≤ MD <−6 dB	85	0.847	0.540	0.305 (0.106, 0.526)
	MD <−12 dB	63	0.866	0.189	0.611 (0.128, 1.000)

MD = mean deviation; OCT = optical coherence tomography; OCT-VF = OCT-based estimated visual field; HFA = Humphrey Field Analyzer; GCC = ganglion cell complex; CI = confidence interval.
Correlation analyses were performed in the GCC analysis dataset, a single-center dataset containing temporally matched HFA, OCT-VF, and macular GCC measurements. For each eye, one MD slope value (for HFA and OCT-VF) and one GCC thickness slope were calculated from longitudinal measurements, irrespective of whether progression criteria were met.
[*]Eyes stratified by mean HFA MD across all measurements. Pearson's r was calculated within each stratum using one slope per eye (MD slopes for HFA and OCT-VF; thickness slope for GCC).
[†]Δ *r* = *r* (OCT-VF, HFA) − *r* (GCC, HFA), evaluated using patient-level cluster bootstrap resampling (B = 10,000). Positive Δ *r* indicates stronger correlation for OCT-VF. All 95% CIs excluded zero (indicating $P < 0.05$).

Table S17. OCT-VF and BAE Parameter Correlations

Parameter	MAE (dB)	ME (dB)
24-2 Pointwise Thresholds	3.33 ± 3.60	–0.44 ± 4.89
10-2 Pointwise Thresholds	3.47 ± 4.00	–0.15 ± 5.29
24-2 MD	2.27 ± 2.09	–0.60 ± 3.03
10-2 MD	2.25 ± 2.12	–0.27 ± 3.08

OCT = optical coherence tomography; OCT-VF = OCT-based estimated visual field; BAE = best available estimate; MAE = mean absolute error; ME = mean error (OCT-VF minus BAE); MD = mean deviation.

MAE and ME were calculated between OCT-VF and the BAE for each parameter. BAE values were obtained per eye and per test location (and for MD) by fitting a simple linear regression to the HFA time series and evaluating the fitted value at the date of the OCT scan. The values are presented as the means ± standard deviations.

Table S18. OCT-VF and BAE Parameter Correlations (GCC Analysis Dataset)

Parameter	MAE (dB)	ME (dB)
24-2 Pointwise Thresholds	2.05 ± 2.53	–0.19 ± 3.25
10-2 Pointwise Thresholds	2.79 ± 3.50	–0.11 ± 4.47
24-2 MD	1.39 ± 1.34	–0.13 ± 1.93
10-2 MD	1.78 ± 1.88	–0.14 ± 2.58

OCT = optical coherence tomography; OCT-VF = OCT-based estimated visual field; BAE = best available estimate; MAE = mean absolute error; ME = mean error (OCT-VF minus BAE); MD = mean deviation.

MAE and ME were calculated between OCT-VF and the BAE for each parameter. BAE values were obtained per eye and per test location (and for MD) by fitting a simple linear regression to the HFA time series and evaluating the fitted value at the date of the OCT scan. The values are presented as the means ± standard deviations. This dataset corresponds to the single-center GCC cohort (Table S11 / Fig. S1).
